# Phase I study of the recombinant humanized anti-HER2 monoclonal antibody–MMAE conjugate RC48-ADC in patients with HER2-positive advanced solid tumors

**DOI:** 10.1007/s10120-021-01168-7

**Published:** 2021-05-04

**Authors:** Yingying Xu, Yakun Wang, Jifang Gong, Xiaotian Zhang, Zhi Peng, Xinan Sheng, Chenyu Mao, Qingxia Fan, Yuxian Bai, Yi Ba, Da Jiang, Fen Yang, Changsong Qi, Jian Li, Xicheng Wang, Jun Zhou, Ming Lu, Yanshuo Cao, Jiajia Yuan, Dan Liu, Zhenghang Wang, Jianmin Fang, Lin Shen

**Affiliations:** 1 Department of Gastrointestinal Oncology, Key Laboratory of Carcinogenesis and Translational Research, (Ministry of Education/Beijing), Peking University Cancer Hospital and Institute, Hai-Dian District, Fu-Cheng Road 52, Beijing, 100142 China; 2 Department of Early Drug Development Center, Peking University Cancer Hospital and Institute, Beijing, China; 3 Department of Medical Oncology, The First Affiliated Hospital of Zhejiang University, Hangzhou, China; 4 Department of Oncology, The First Affiliated Hospital of Zhengzhou University, Zhengzhou, Henan China; 5 Gastrointestinal Oncology Department, Harbin Medical University Cancer Hospital, Harbin , Heilongjiang China; 6 Key Laboratory of Cancer Prevention and Therapy, Tianjin Medical University Cancer Institute and Hospital, National Clinical Research Center for Cancer, Tianjin’s Clinical Research Center for Cancer, Tianjin, China; 7 Department of Medical Oncology, the Fourth Hospital of Hebei Medical University, Shijiazhuang, Hebei China; 8 Key Laboratory of Carcinogenesis and Translational Research (Ministry of Education), National Drug Clinical Trial Center, Peking University Cancer Hospital and Institute, Beijing, China; 9 School of Life Science and Technology, Tongji University, Shanghai, China; 10 Department of Renal Cancer and Melanoma, Peking University Cancer Hospital and Institute, Beijing, China

**Keywords:** RC48-ADC, HER2, Solid tumors, Gastric cancer

## Abstract

**Purpose:**

RC48 contains the novel humanized anti-HER2 antibody hertuzumab conjugated to MMAE via a cleavable linker. A phase I study was initiated to evaluate the toxicity, MTD, PK, and antitumor activity of RC48 in patients with HER2-overexpressing locally advanced or metastatic solid carcinomas, particularly gastric cancer.

**Patients and methods:**

This was a 2-part phase I study. Successive cohorts of patients received escalating doses of RC48 (0.1 mg/kg, 0.5 mg/kg, 1.0 mg/kg, 2.0 mg/kg, 2.5 mg/kg, and 3.0 mg/kg). Dose expansion proceeded at the dose of 2.0 mg/kg Q2W. The efficacy and safety set included all patients who received at least one dose of RC48.

**Results:**

Fifty-seven patients were enrolled, the MTD was unavailable due to termination of 3.0 mg/kg cohort; 2.5 mg/kg Q2W was declared the RP2D. RC48 was well tolerated, the most frequent grade 3 or worse TRAEs included neutropenia (19.3%), leukopenia (17.5%), hypoesthesia (14.0%), and increased conjugated blood bilirubin (8.8%). Four deaths occurred during the whole study, three of which were believed to be related to RC48. Overall, ORR and DCR were 21.0% (12/57) and 49.1% (28/57). Notably, patients who were HER2 IHC2+/FISH- responded similarly to those who were IHC2+/FISH+ and IHC3+, with ORRs of 35.7% (5/14), 20% (2/10), and 13.6% (3/22), respectively. In patients who were pretreated with HER2-targeted drugs, RC48 also showed promising efficacy, with ORR of 15.0% (3/20) and DCR of 45.0% (9/20).

**Conclusion:**

RC48 was well tolerated and showed promising antitumor activity in HER2-positive solid tumors, including gastric cancer with HER2 IHC 2+/FISH- status.

**Clinical trial information:**

NCT02881190.

**Supplementary Information:**

The online version contains supplementary material available at 10.1007/s10120-021-01168-7.

## Introduction

Approximately 20% of metastatic gastric cancer patients have HER2 overexpression or amplification. Although trastuzumab in combination with chemotherapy has become the standard of care in first-line treatment of HER2-positive metastatic gastric cancer [1], until recently, the treatment options after progression on trastuzumab have been limited. For patients who have progressed on trastuzumab, continuous use of trastuzumab (trastuzumab beyond progression, TBP) failed to improve PFS in patients with HER2-positive advanced G/GEJ cancer. Re-biopsy analysis revealed that HER2-positivity of tumor tissues obtained from 16 patients before the study entry was lost in 69% [2], and another study in Japan showed that loss of HER2 was identified in 60.6% of patients [3]. In addition, several other agents have failed to show efficacy for HER2-positive gastric cancer refractory to trastuzumab, including pertuzumab, lapatinib, and T-DM1 [4–8]. HER2-positive advanced gastric cancer has been found not only to share some of mechanisms of resistance with breast cancer, but also to manifest specific mechanisms of resistance to trastuzumab, including tumor heterogeneity in HER2 positivity, loss of HER2 protein expression, alteration in HER2 downstream signaling, and activation of bypass pathways [9]. Therefore, the development of new HER2-targeted therapeutic approaches should consider the challenges posed by high levels of heterogeneity and complex mechanisms of resistance.

Currently, novel agents and combinations are being actively investigated in HER2-positive gastric cancers. Antibody–drug conjugates (ADC), comprised of an antibody against the antigen of interest, a linker, and a payload cytotoxic agent, are designed for specific delivery of cytotoxic agents to malignant cells [10–12]. RC48 contains the novel humanized anti-HER2 antibody hertuzumab conjugated to monomethyl auristatin E (MMAE) via a cleavable linker. Compared to trastuzumab, hertuzumab has a higher affinity for HER2 and more potent antibody-dependent cell-mediated cytotoxicity (ADCC) activity in vitro. The binding specificity of this drug for HER2 was not affected by conjugation to MMAE. Furthermore, the internalization of hertuzumab-vcMMAE in HER2-positive gastric cancer cells was verified. Although the conjugation of hertuzumab to MMAE decreased the ADCC effect, the overall cytotoxicity dramatically increased in HER2-positive gastric cancer cells [13]. In vitro, RC48 has exerted much stronger antitumor activity compared to T-DM1, an FDA-approved ADC drug, in HER2 positive breast and gastric cancer cells, also in the trastuzumab- and lapatinib-resistant xenograft tumor models, suggesting its potential as an improved therapy for HER2-positive cancers [14]. More importantly, preclinical experiments demonstrated significant anti-tumor activity via a bystander effect where HER2 overexpressing cells are recognized by RC48, but nearby HER2-negative cells in co-culture also underwent apoptosis. This bystander effect appears unique to RC48 as such anti-tumor activity in preclinical experiments was not observed with T-DM1, which mainly depends on the presence of a cleavable linker-a dipeptide valine–citrulline (vc) linker [15, 16]. Thus, RC48 could be a promising agent whose mechanism of action may overcome resistance caused by the intratumoral heterogeneity of HER2 overexpression and outgrowth of HER2-negative clones.

We initiated a dose escalation and expansion phase I study of single-agent of RC48. First, we aimed to assess the safety, tolerability, and PK of this drug in patients with HER2-positive solid tumors, particularly in HER2-positive gastric cancer. Second, based on the greater antitumor activity and bystander effect observed in preclinical models, RC48 is considered useful for treating tumors with heterogeneous antigen expression. Therefore, we aimed to investigate the clinical efficacy of RC48 in gastric cancer with low HER2 expression, including IHC 2+/FISH−.

## Methods

### Study design

This was a phase I, open-label, multicenter, interventional two-part study, which included dose escalation and dose expansion parts. Patients were enrolled from six study sites in China.

According to the “Guiding Principles for Clinical Trial Techniques of Anti-Tumor Drugs” and “Technical Guidelines for Non-Clinical Studies of Cytotoxic Anti-Cancer Drugs” issued by China, the initial dose of Phase I clinical trials was estimated as follows: the MTD obtained from rat multiple-dose toxicological study was 12 mg/kg, with the equivalent dose for human was 1.92 mg/kg and the safety factor was 10. The calculation of 1/10 gave the initial dose of 0.192 mg/kg. As a related animal, crab-eating macaque was given multiple doses (Q2W × 7) during toxicological study, providing an MTD of 10 mg/kg. The human equivalent dose was 3.2 mg/kg and the calculation of 1/6 gave the initial dose of 0.53 mg/kg. According to the FDA's “Estimating the Maximum Safe Starting Dose in Initial Clinical Trials for Therapeutics in Adult Healthy Volunteers”, the starting dose was calculated to be 0.096 mg/kg, which was approximately equal to 0.1 mg/kg.

The following dose escalations were proposed: 0.1 mg/kg, 0.5 mg/kg, 1.0 mg/kg, 1.5 mg/kg, 2.0 mg/kg, 2.5 mg/kg, 3.0 mg/kg, 3.5 mg/kg, and 4.0 mg/kg. To reduce the patient's exposure to an ineffective dosage, one subject was enrolled in the first-dose group (0.1 mg/kg) and the second-dose group (0.5 mg/kg), respectively. Starting with the third-dose group (1.0 mg/kg), the subjects were enrolled using the traditional 3 + 3 model. Based on the mean half-life (*t*_1/2_) and PK results of ADC and free MMAE of the two subjects enrolled in the 0.1 mg/kg and 0.5 mg/kg single-dose group, the Q2W was selected as dosing frequency for further evaluation. Considering combining RC48 with other drugs in future trials, particularly immune checkpoint inhibitors, most of which were given Q3W, the investigation of Q3W dosing of RC48 was designed to add convenience for patients, which was a preliminary investigation with only three patients administered with 2.0 mg/kg Q3W in this trial.

Dose-limiting toxicity (DLT) was defined as the following toxic reactions observed in the DLT evaluation window (observation period of days 1–21 after the first dose) that were believed to be related to RC48 treatment by the investigators, wherein the grading was based on the 5-level system of the CTCAE v4.0: grade 4 neutropenia lasting more than 3 days after G-CSF treatment; grade 4 neutropenia recurring after recovery to a normal level through G-CSF treatment; neutropenic fever (defined as an absolute neutrophil count [ANC] < 1000/mm^3^ accompanied by a fever higher than 38.3 °C or a fever higher than 38 °C that persists for more than 1 h); grade 3 neutropenia with confirmed infections; grade 3 thrombocytopenia with a bleeding tendency; grade 4 thrombocytopenia; a non-hematologic toxicity of grade 3 or higher except for nausea, vomiting, and hair loss after supportive care; renal toxicity ≥ grade 3; neurotoxicity ≥ grade 2, with no response before the next medication; cardiac toxicity ≥ grade 2; and for patients with hepatic transaminases reaching grade 2 at baseline, hepatic transaminases increasing to a level ≥ 10 × ULN was considered a DLT.

If DLT appeared in subjects, treatment with RC48 would first be discontinued, and adverse reactions would be actively addressed until the toxicity returns to ≤ Grade 1. Thereafter, medication should proceed at a decreased dose level (specific doses will be determined by the investigators). The MTD was defined as dosage below the dose with which DLT occurred in two or more out of six patients during the DLT evaluation window.

The study was performed in accordance with the Declaration of Helsinki and the International Conference on Harmonisation Guidelines for Good Clinical Practice. Approval by an independent ethics committee or institutional review board was obtained before study initiation.

### Patients

Patients with incurable, locally advanced or metastatic solid cancers were eligible for inclusion if their tumors showed HER2 protein overexpression by IHC (3+ or 2+), regardless of whether FISH was positive or negative. However, FISH results were necessary in the dose expansion section of the study for efficacy analysis. Eligible patients were refractory to standard treatment, had no standard treatment, or for whom standard treatment was intolerable or unavailable. Trastuzumab combined with chemotherapy has been considered the standard regimen in first-line treatment. However, trastuzumab is not covered by medical insurance in some areas of China, and some patients could not afford it. On the other hand, there is no standard of care established for HER2-positive gastric cancer in later lines of treatment. Therefore, these patients were allowed to participate in this clinical trial to determine potential benefits.

Eligible patients were with an Eastern Cooperation Oncology Group (ECOG) performance status of 0 or 1, had a left ventricular ejection fraction (LVEF) of at least 50%. There were measurable or evaluable lesions according to RECIST 1.1, and adequate bone marrow, liver, and kidney function (absolute neutrophils count[ANC] ≥ 1.5*10^9^/L; platelet ≥ 100*10^9^/L; serum total bilirubin ≤ 1.5 times the upper limit of normal value[ULN]; for those without liver metastasis, ALT AST or ALP ≤ 2.5 ULN, for those with liver metastasis, ALT AST or ALP ≤ 5 ULN; serum creatinine was normal; INR ≤ 1.5 ULN, APTT ≤ 1.5 ULN).

Key exclusion criteria included serious complications, such as active gastrointestinal bleeding, intestinal obstruction, or history of acute myocardial infarction, congestive heart failure (NYHA) ≥ Grade 2. Patients who had uncontrolled primary or metastatic brain tumors were also excluded. Patients with the following treatments were permitted to be enrolled with adequate washout period: major surgery (≥ 4 weeks), radiotherapy (≥ 2 weeks), chemotherapy (including antibody drug therapy [≥ 3 weeks]).

Full inclusion and exclusion criteria are listed in the study protocol. Written informed consent was obtained from all patients before study enrollment.

### Pharmacokinetics (PK)

Serial blood samples for the PK characterization of total antibody (TA), binding antibody (BA), and free MMAE (FM) were collected during cycles 1 and 4 at the following timepoints: predose, and 0.25, 1 (end of infusion), 1.5, 12, 24, 48, 72, 120, 168, 240, and 360 h after the start of infusion. The TA and BA concentrations in serum were measured using a validated mesoscale discovery (MSD) method and liquid chromatography-tandem mass spectrometry (LC–MS/MS) for detection of FM. Several additional blood samples were chosen to detect the conjugated MMAE (CM) using the same LC–MS/MS method from three patients who received multiple doses of RC48 at 2.5 mg/kg.

PK parameters such as maximum observed serum concentration (*C*_max_) and time to *C*_max_ (*T*_max_) were directly obtained from the measured plasma concentration. Other main PK parameters were calculated from the plasma concentration–time data using a noncompartmental analysis (NCA) method with WinNonlin 6.3 (Pharsight Corp., Mountain View, CA, USA) and included elimination half-life (*t*_1/2_), the area under the plasma concentration–time curve from zero to the last time (AUC_0−*t*_) and from zero to infinity (AUC_0−Inf_), apparent total body clearance (CL/F), and apparent volume of distribution (*V*_d_/*F*).

### Safety and efficacy assessment

The primary end point of this study is to evaluate the safety and most tolerated dose (MTD) of RC48 in HER2-positive advanced solid tumors and to explore the recommended phase 2 dose (RP2D) in future studies. Secondary outcomes included assessment of pharmacokinetic (PK) characteristics and preliminary clinical efficacy of RC48 in patients with advanced HER2-positive solid tumors.

All subjects were observed for any adverse events that occurred during the clinical study according to NCI-CTC AE (4.0), including clinical symptoms, signs of life, and abnormal in laboratory tests. Radiologic assessment was evaluated at baseline and every 2 cycles (for Q2w, 2 doses as a cycle) or 3 cycles (for Q3w, 1 dose as a cycle) of treatment. Tumors were assessed by CT or MRI of the chest, abdomen, and pelvis. RECIST v1.1 criteria were used to evaluate the objective response rate (ORR), disease control rate (DCR). Initial response evaluation as complete response (CR) or partial response(PR) need to be confirmed after 4 weeks.

### Statistical analysis

Descriptive statistics were mainly used because of the small sample size. Descriptive statistics for pharmacokinetic parameters (mean, standard deviation, coefficient of variation, minimum, median, and maximum) were provided for the list of drugs tested. The incidence of all adverse events (AE) was listed by treatment group. The adverse events were recorded using the terms of the investigator and a statistical description of MedDRA. All statistical analyses were performed using SAS®9.3.

## Results

### Enrollment, characteristics, and determination of MTD

From December 22, 2015, to June 27, 2019, 68 patients were screened. Fifty-seven patients met the inclusion criteria and were sequentially enrolled into six dose groups. The most common tumor type among enrolled patients was gastric cancer. All 57 patients had measurable lesions at baseline. Baseline characteristics are summarized in Table 1.

**Table 1:** Baseline patient demographics and clinical characteristics (*N* = 57)

Characteristics		0.1 mg/kg *N* = 1(%)	0.5 mg/kg *N* = 1(%)	1.0 mg/kg Q2W *N* = 3(%)	2.0 mg/kg Q2W *N* = 35(%)	2.0 mg/kg Q3W *N* = 3(%)	2.5 mg/kg Q2W *N* = 11(%)	3.0 mg/kg Q2W *N* = 3(%)	Total *N* = 57(%)
Age, years									
Range		66– 66	65 –65	58 –60	39–73	46–75	42–72	28–54	28–75
Median		66.0	65.0	60.0	59.0	72.0	59.0	49.0	59.0
Sex									
Male		1 (100.0)	1 (100.0)	1 (33.3)	28 (80)	3 (100)	8 (72.7)	2 (66.7)	44 (77.2)
Female		0 (0)	0 (0)	2 (66.7)	7 (20)	0 (0)	3 (27.3)	1 (33.3)	13 (22.8)
ECOG PS									
0		0 (0)	0 (0)	0 (0)	16 (45.7)	1 (33.3)	7 (63.6)	0 (0)	24 (42.1)
1		1 (100.0)	1 (100.0)	3 (100.0)	19 (54.3)	2 (66.6)	4 (36.4)	3 (100.0)	33 (57.9)
Time since initial diagnosis, years									
Range		1–1	1–1	3–7	0.2–5.1	1.0–1.6	0.7–3.5	0.9–3.8	0.2–7
Median		1.00	1.00	6.00	1.30	1.2	1.20	1.60	1.30
Cancer types									
Gastric cancer		1 (100.0)	1 (100.0)	2 (66.7)	26 (74.3)	3 (100)	11 (100.0)	3 (100.0)	47 (82.5)
Urothelial cancer		0 (0)	0 (0)	0 (0)	4 (11.4)	0 (0)	0 (0)	0 (0)	4 (7.0)
Others^a^		0 (0)	0 (0)	1 (33.3)	5 (14.3)	0 (0)	0 (0)	0 (0)	6 (10.5)
HER2 status									
IHC3+	0 (0)		0 (0)	2 (66.7)	13 (37.1)	0 (0)	4 (36.4)	0 (0)	19 (33.3)
IHC3+ FISH+	0 (0)		0 (0)	0 (0)	5 (14.3)	0 (0)	3 (27.3)	0 (0)	8 (14.0)
IHC3 + FISH−	0 (0)		0 (0)	0 (0)	0 (0)	0 (0)	0 (0)	0 (0)	0 (0)
IHC2+	0 (0)		1 (100.0)	0 (0)	2 (5.7)	0 (0)	0 (0)	0 (0)	3 (5.3)
IHC2+ FISH+	1 (100.0)		0 (0)	1 (33.3)	4 (11.4)	2 (66.6)	2 (18.2)	0 (0)	10 (17.5)
IHC2+ FISH−	0 (0)		0 (0)	0 (0)	11 (31.4)	1 (33.3)	2 (18.2)	3 (100.0)	17 (29.8)
Previous anti-HER2 therapy									
Trastuzumab	0 (0)		1 (100.0)	1 (33.3)	11 (31.4)	1 (33.3)	6 (54.5)	0 (0)	20 (35.1)
Pertuzumab	0 (0)		0 (0)	0 (0)	1 (2.8)	0 (0)	2 (18.2)	0 (0)	3 (5.3)
Pyrotinib	0 (0)		1 (100.0)	0 (0)	1 (2.8)	0 (0)	0 (0)	0 (0)	2 (3.5)
Afatinib	0 (0)		0 (0)	0 (0)	1 (2.8)	0 (0)	0 (0)	0 (0)	1 (1.8)
No	1 (100.0)		0 (0)	2 (66.7)	23 (65.7)	2 (66.6)	5 (45.5)	3 (100.0)	36 (63.2)
Numbers of previous cancer regimens									
1	1 (100.0)		0 (0)	1 (33.3)	12 (34.3)	3 (100)	5 (45.5)	2 (66.7)	24 (42.1)
2	0 (0)		1 (100.0)	0 (0)	13 (37.1)	0 (0)	4 (36.4)	0 (0)	18 (31.6)
≥ 3	0 (0)		0 (0)	2 (66.7)	10 (28.6)	0 (0)	2 (18.2)	1 (33.3)	15 (26.3)

^a^There were 6 patients with cancer types of others, including 1 patient with breast cancer, 1 patient with ampullary cancer, 3 patients with colorectal cancer, 1 patient with gallbladder cancer

**Fig. 1:**
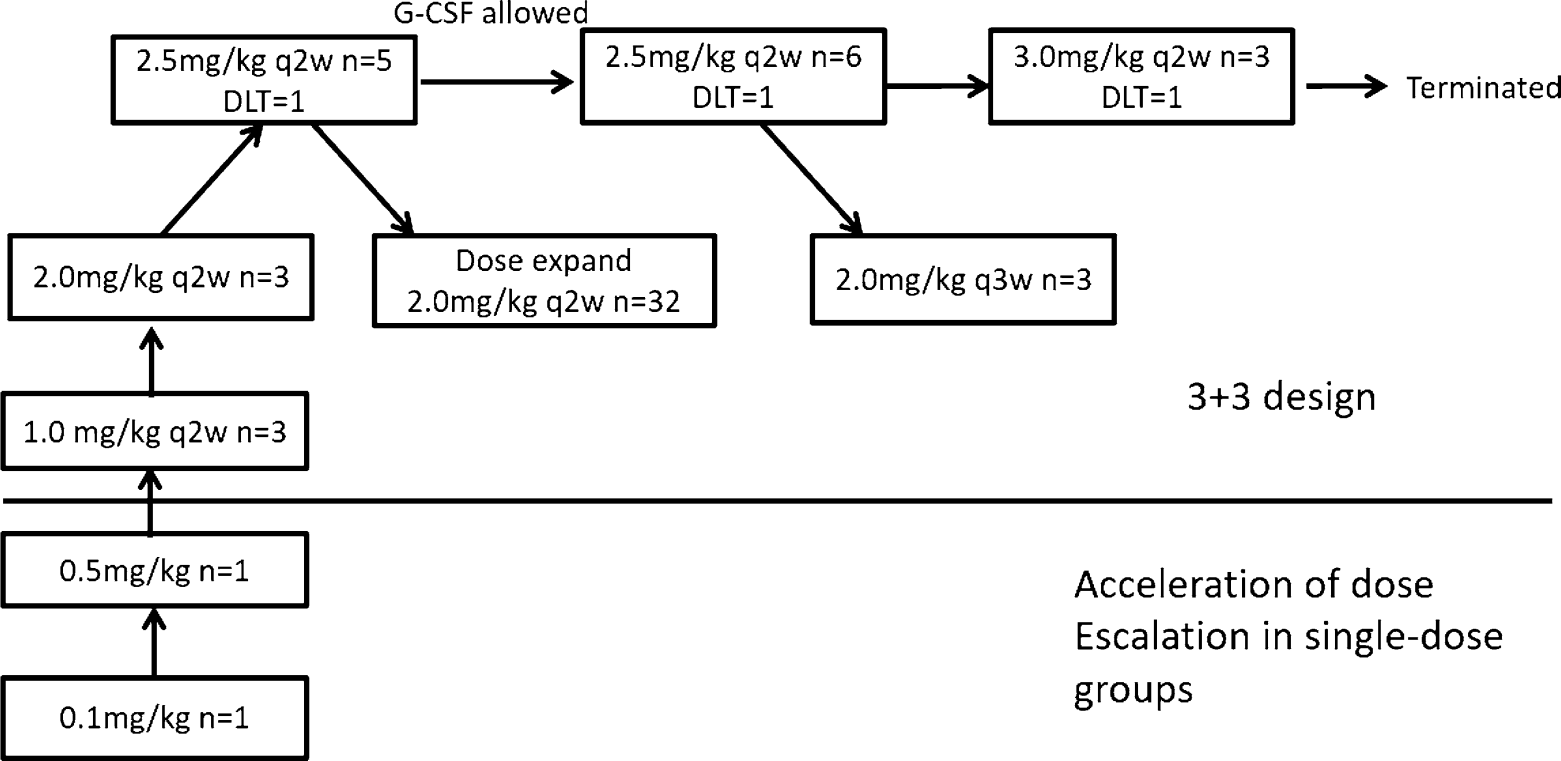
The process of dose escalation and expansion

Each of the 0.1 mg/kg and 0.5 mg/kg cohorts enrolled one patient, with no observed drug-related adverse events of Grade > 2. From 1.0 mg/kg dose cohort 3 + 3 dose escalation was used. No DLT occurred in 1.0 mg/kg and 2.0 mg/kg cohort. Three patients were enrolled in 2.5 mg/kg group and 1 DLT occurred due to Grade 4 neutropenia. After discussion with investigators and with approval by ethics committee, G-CSF was allowed from the 2.5 mg/kg group in order to observe safety of long-term therapy. After three patients were treated at the dose of 3.0 mg/kg, one DLT occurred. This patient died 13 days after the first dose of RC48. The cause of death was considered to be septic shock by investigators, possibly related to marrow toxicity of RC48. Considering there was no trend of improving efficacy after adding the dose to 3.0 mg/kg dose compared with 2.0 mg/kg and 2.5 mg/kg cohorts, and continuing enrolling patients in 3.0 mg/kg group may significantly increase the risk of subjects, after discussion the investigators decided to terminate the enrollment of the 3.0 mg/kg cohort and reported to institutional review board. The MTD was unavailable due to the termination of the 3.0 mg/kg cohort and 2.5 mg/kg was declared the RP2D. After discussion about PK, adverse events and efficacy of RC48 in the dose escalation part, 2.0 mg/kg Q2W was chosen as the dose used in the expansion part. Finally, the expansion cohort was expanded to a total of 38 patients. The process of dose escalation and expansion is summarized in Fig. 1.

### Treatment-related toxicities

In the adverse event (AE) analysis, patients who received at least one dose of RC48 were analyzed altogether (*N* = 57).

A total of 94.7% (54/57) of patients had AEs in the first 21 days after treatment. Three (5.3%) patients had DLT-related AEs, including neutropenia (*N* = 2) and thrombocytopenia (*N* = 1). DLT was observed in only the high-dose cohorts (2.5 mg/kg and 3.0 mg/kg) (Table S1), indicating DLT of RC48 was dose-dependent.

Types and incidence of related AEs in each cohort were listed in Table 2. As reported, the most common treatment-related adverse events (TRAEs) were blood and lymphatic system disorders, gastrointestinal disorders, and nerve toxicities, which were mostly classified as Grade 1 or 2. The most frequent Grade 3 or worse TRAEs included neutropenia (19.3%), leukopenia(17.5%), hypoesthesia (14.0%), and conjugated blood bilirubin increased(8.8%). Notably, patients had similar tolerability in 2.0 mg/kg and 2.5 mg/kg cohort.

**Table 2:** Common adverse events related to study drug in each cohort (*N* = 57)

	0.1 mg/kg *N* = 1		0.5 mg/kg *N* = 1		1.0 mg/kg Q2W *N* = 3		2.0 mg/kg Q2W *N* = 35		2.0 mg/kg Q3W *N* = 3		2.5 mg/kg Q2W *N* = 11		3.0 mg/kg Q2W *N* = 3		Total *N* = 57
	Grade 1/2	Grade 3/4	Grade 1/2	Grade 3/4	Grade 1/2	Grade 3/4	Grade 1/2	Grade 3/4	Grade 1/2	Grade 3/4	Grade 1/2	Grade 3/4	Grade 1/2	Grade 3/4	All grades
White blood cell decreased	0	0	0	0	1	0	18	1	3	0	4	7	1	2	37
Neutrophil count decreased	1	0	0	0	1	0	12	2	1	1	5	6	1	2	32
Anemia	1	0	1	0	2	0	14	1	3	0	4	1	2	1	30
Platelet count decreased	0	0	0	0	0	0	4	2	1	0	4	0	1	1	13
AST increased	0	0	0	0	2	0	8	3	2	0	7	0	1	1	24
ALT increased	0	0	0	0	1	0	8	2	1	0	7	0	2	0	21
Blood bilirubin increased	0	0	0	0	2	0	8	0	1	0	3	0	0	1	15
Conjugated bilirubin increased	0	0	0	0	1	1	5	3	2	0	6	0	1	1	20
Unconjugated bilirubin increased	0	0	0	0	0	0	3	0	1	0	1	0	1	0	6
Hypaesthesia	0	0	0	0	0	0	12	4	2	1	3	3	0	0	25
Fatigue	0	0	1	0	0	0	18	1	2	0	7	2	1	0	32
Fever	0	0	0	0	0	0	5	0	1	0	5	0	2	0	13
Hair loss	0	0	0	0	0	0	14	0	2	0	9	0	2	0	27
Pruritus	0	0	0	0	0	0	6	0	0	0	4	0	1	0	11
Skin rash	0	0	0	0	1	0	5	0	1	0	2	0	0	0	9
Nausea	0	0	0	0	0	0	12	0	1	0	6	0	0	0	19
Diarrhea	1	0	0	0	0	0	7	0	2	0	4	0	0	0	14
Vomiting	0	0	0	0	0	0	6	1	0	0	3	0	1	0	11
Loss of appetite	0	0	0	0	0	0	10	0	1	0	3	0	2	0	16
Arthralgia	0	0	0	0	0	0	4	0	0	0	2	0	0	0	6
Proteinuria	0	0	0	0	0	0	4	1	0	0	0	0	0	0	5
Dizziness	0	0	0	0	0	0	1	0	0	0	0	0	0	0	1
Abdominal pain	0	0	0	0	0	0	0	0	1	0	0	0	0	0	1

Treatment-related serious adverse events (SAEs) occurred in 17 patients (29.8%): all cases occurred in 2.0 mg/kg and 2.5 mg/kg cohort. The most common treatment-related SAE was fever (in 5.3% patients), incomplete ileus (3.5%), elevated ALT (3.5%), elevated AST (3.5%), and death (5.3%).

Four deaths occurred during the whole study. One death was due to biliary tract infection, and two deaths were due to febrile neutropenia. Another patient developed vomiting of Grade 3 during the third cycle of treatment and died one day after hospitalization in another hospital. This death was considered related to tumor progression instead of the study drug by the investigator. Three of the four deaths were in the 2.0 mg/kg cohort, and one was in the 3.0 mg/kg cohort (febrile neutropenia).

TRAEs resulting in dose reduction or interruption occurred in the 2.0 mg/kg (7.9% or 47.4%), 2.5 mg/kg (36.4% or 45.5%), and 3.0 mg/kg (33.3% or 66.7%) cohorts. Common AEs included neutropenia, leukopenia, hypoesthesia, fatigue, and elevated ALT/AST. Permanent treatment discontinuation due to TRAEs occurred in 31.6% and 9.1% of patients in the 2.0 mg/kg and 2.5 mg/kg cohorts, respectively. The most commonly reported AEs leading to permanent treatment discontinuation were hypoesthesia, fatigue, and death.

### Anti-tumor activity

The efficacy of RC48 was evaluated in FAS (full analysis set). Therefore, patients who received at least one dose of RC48 were analyzed altogether (*N* = 57). In all the 57 patients, including 47 gastric cancer patients, 4 urothelial cancer patients, and 6 other cancer patients. 21.1% (12/57) patients had an PR, 10 of which were confirmed. No complete responses were observed. DCR of the evaluable patients was 49.1% (28/57). Detailed efficacy was listed in Table 3.

**Table 3:** Clinical activity of RC48 in patients based on the RECIST 1.1 criteria (FAS)

	0.1 mg/kg *N* = 1	0.5 mg/kg *N* = 1	1.0 mg/kg Q2W *N* = 3	2.0 mg/kg Q2W *N* = 35	2.0 mg/kg Q3W *N* = 3	2.5 mg/kg Q2W *N* = 11	3.0 mg/kg Q2W *N* = 3	Total *N* = 57
CR	0	0	0	0	0	0	0	0
PR	0	0	0	6	2	2	0	10
Unconfirmed PR	0	0	0	0	0	1	1	2
SD	1	0	0	11	1	3	0	16
PD	0	1	2	11	0	5	1	20
Unevaluable	0	0	1	7	0	0	1	9
ORR	0	0	0	17.1%	66.6%	27.3%	33.3%	21.1%
DCR	100%	0	0	48.6%	100%	64.5%	33.3%	49.1%
mPFS (m)	5.7 (–)	1.4 (–)	1.2 (1.0–1.4)	3.6 (1.9–7.5)	16 (9.8–27.1)	1.9 (1.1–7.9)	NA (1.5-)	3.5 (1.9–5.3)

Antitumor activity of RC48 was dose-dependent. No response was observed in the low-dose group (0.1, 0.5, and 1.0 mg/kg). 17.1% PR occurred in 2.0 mg/kg Q2W cohort and 27.3% in 2.5 mg/kg Q2W cohort.

**Fig. 2:**
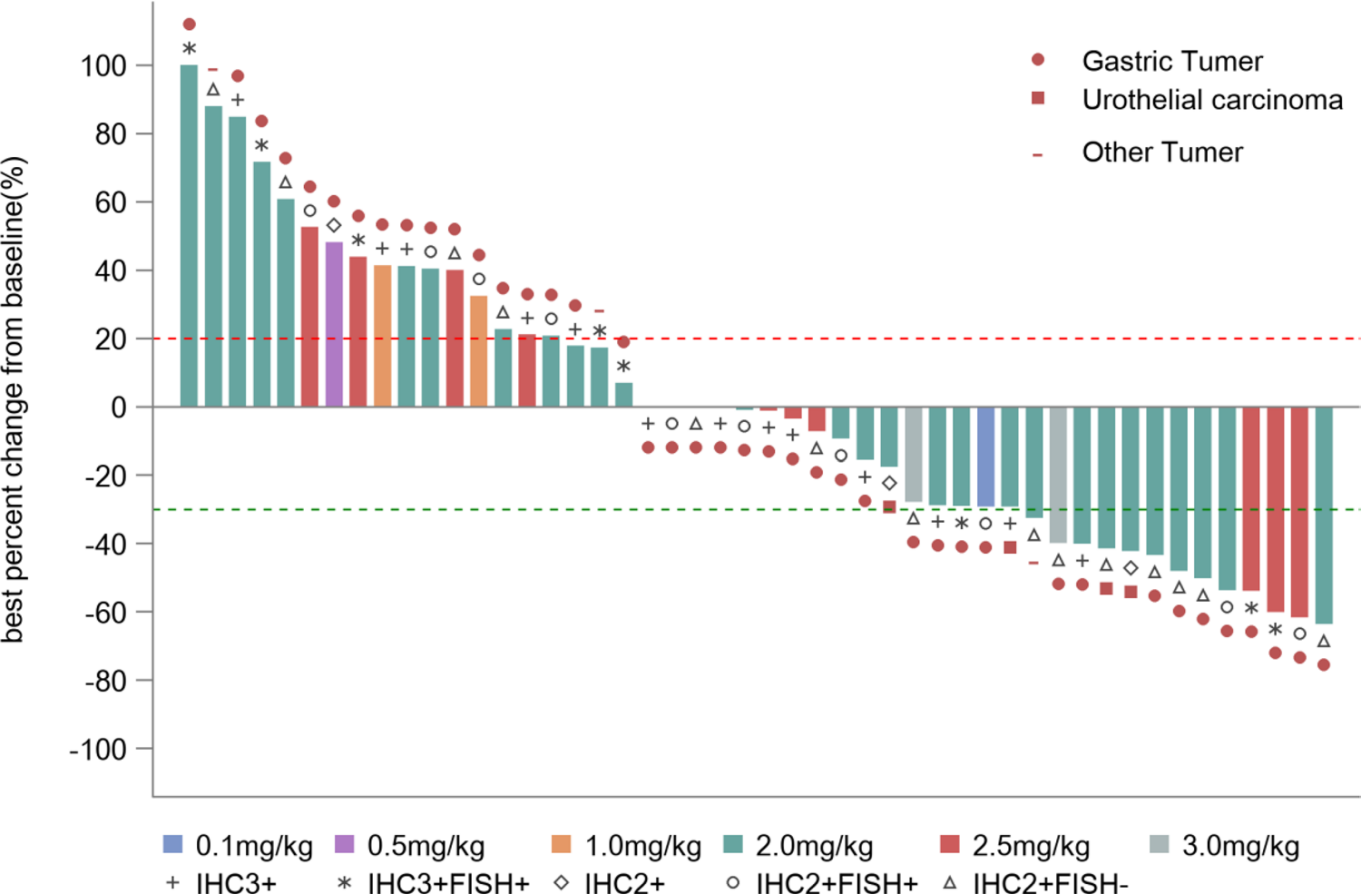
Best percentage change to date from baseline in sum of diameters in target lesions. Maximal change of tumor size from baseline assessed by investigator per RECIST v1.1 from all patients with baseline and at least one post-treatment radiographic evaluation (*N* = 48)

Median PFS of 57 patients was 3.5 m (95% CI: 1.9–5.3). The efficacy and response duration of all patients who were available for the assessment of response (*N* = 48) are shown in Fig. 2 and Figure S2.

In the analysis of the gastric cancer subgroup, the ORR was 21.3% (10/47), confirmed ORR was 17.0% (8/47), and the DCR was 46.8% (22/47). In the 2.0 mg/kg and 2.5 mg/kg cohorts, the ORR was 20.7% (6/29) and 27.3% (3/11), respectively. HER2 IHC 2+/FISH− gastric cancer patients were considered as a group that cannot benefit from trastuzumab in previous studies [1]. In this study, the ORRs in gastric cancer patients with HER2 IHC 2+/FISH−, IHC 2+/FISH+, and IHC 3+ statuses were 35.7% (5/14), 20% (2/10), and 13.6% (3/22), respectively. One gastric cancer patient with HER2 IHC2+/FISH (unknown) progressed after the first evaluation. 27 patients enrolled in this study have not received HER2-targeting therapy before. ORR and DCR was 25.9% (7/27) and 48.1% (13/27) in this population, respectively. Also, in patients who were pretreated with HER2-targeted drugs, RC48 also showed promising efficacy, with an ORR of 15.0% (3/20) and a DCR of 45.0% (9/20) (Table S2).

In addition, there were two PR and two stable disease (SD) patients among the four urothelial patients, with a confirmed ORR of 50% and a DCR of 100%. There was no PR in the six patients of other cancer types.

### Immunogenicity and PK assessments

The mean serum concentration of TA, BA, FM and CM vs. time curves of patients who received RC48 at different dose levels are shown in Figure S1. The corresponding PK parameters of TA, BA and FM are presented in Table 4. Both TA and BA analytes reached their maximum-observed serum concentration (*C*_max_) almost at the end of the RC48 infusion. *C*_max_ and AUC_0−inf_ of TA were approximately 1- to 1.5-fold and 1- to 1.8-fold higher than those of BA within the dose ranging from 0.1 to 3.0 mg/kg (mean *C*_max_: 7.0E2–7.8E4 ng/mL and 7.0E2–5.2E4 ng/mL for TA and BA, respectively; mean AUC_0–inf_: 8.5E3–2.3E6 h*ng/mL and 6.7E3–1.2E6 h*ng/mL for TA and BA, respectively). After reaching their *C*_max_, concentrations of the two analytes declined in a multi-exponential manner. TA and BA analytes showed similar decline profile in concentration (Figure S1). As a result, they exhibited approximately same half-lives while the generally shorter clearance were observed for TA (Table 4). In addition, the corresponding systemic exposures and half-lives showed positive correlation with the dose-escalation from 0.1 to 3 mg/kg.

**Table 4:** Pharmacokinetic parameters for total antibody (TA), binding antibody (BA) and free MMAE(FA) at different dose levels

		Single dose					Multiple dose
PK parameters	0.1 mg/kg N = 1	0.5 mg/kg N = 1	1.0 mg/kg N = 3	2.0 mg/kg N = 6	2.5 mg/kg N = 11	3.0 mg/kg N = 3	2.5 mg/kg N = 11
*C*_max_ (ng/mL)							
TA	697	4.2E3	1.3E4 (4.0E3)	4.5E4 (6.2E4)	5.6E4 (2.4E4)	7.8E4 (4.3E4)	4.1E4 (1.1E4)
BA	677	9.5E3	1.3E4 (2.1E3)	3.8E4 (3.6E4)	5.3E4 (2.6E4)	5.2E4 (1.8E4)	3.7E4 (6164)
FM	NA	1	2 (1)	5 (4)	6 (2)	13 (4)	6 (4)
AUC0−*t* (h*ng/mL)							
TA	7.0E3	1.2E5	2.6E5 (4.4E4)	1.3E6 (4.1E5)	1.9E6 (5.6E5)	2.3E6 (6.1E5)	1.9E6 (6.3E5)
BA	6.0E3	1.3E5	2.0E5 (6.0E4)	7.3E5 (2.6E6)	1.1E6 (2.7E5)	1.2E6 (2.9E5)	1.0E6 (9.5E4)
FM	NA	118	356 (233)	746 (521)	912 (440)	2.4E3 (672)	865 (251)
AUC0−inf (h*ng/mL)							
TA	8.5E3	1.2E5	2.6E5 (4.3E4)	1.4E6 (4.2E5)	1.9E6 (5.8E5)	2.3E6 (6.2E5)	2.0E6 (4.9E5)
BA	6.7E3	1.4E5	2.1E5 (6.1E4)	7.4E5 (2.6E5)	1.1E6 (2.6E5)	1.2E6 (2.9E5)	1.2E6 (2.3E5)
FM	NA	133	376 (237)	850 (664)	990 (551)	2.0E3 (206)	896 (210)
*T*_max_ (h)							
TA	0.58	0.65	1.52 (1.03–1.52)	1.48 (1.02–1.53)	1.08 (0.2–1.53)	1.5 (1.07–1.55)	1.14 (1.03–1.5)
BA	0.58	0.65	1.52 (1.03–1.52)	1.10 (1.02–1.47)	1.08 (0.2–1.53)	1.5 (1.07–1.55)	1.48 (1.17–1.53)
FM	NA	47.98	48.25 (48.15–72)	48.06 (23.95–71.67)	71.67 (47.18–119.6)	47.18 (46.87–119.07)	47.15 (22.6–71.6)
*t*1/2z (h)							
TA	9.6	12.5	15.9 (2.5)	33.9 (8.6)	36.8 (9.2)	42.2 (0.4)	44.1 (12.4)
BA	7.2	22.0	16.2 (9.5)	33.2 (11.6)	45.7 (17.6)	45.5 (8.3)	103.1 (93.7)
FM	NA	44.8	63.8 (12.7)	68.6 (46.8)	61.3 (18.0)	38.3 (15.5)	81.3 (27.7)
*V*_z_ (mL/kg)							
TA	163.5	73.6	87.0 (10.9)	76.0 (23.9)	72.4 (14.7)	82.4 (19.9)	86.8 (46.1)
BA	154.7	115.2	108.8 (54.7)	150.3 (81.9)	158.5 (80.4)	170.7 (65.9)	340.8 (299.6)
FM	NA	2.4E5	4.0E5 (3.7E5)	3.1E5 (2.0E5)	2.6E5 (1.1E5)	8.0E4 (2.5E4)	4.1E5 (2.5E5)
CL_z_ (mL/h/kg)							
TA	11.8	4.1	3.8 (0.6)	1.6 (0.5)	1.4 (0.4)	1.4 (0.3)	1.3 (0.3)
BA	14.9	3.6	5.2 (1.7)	3.2 (1.6)	2.3 (0.5)	2.6 (0.7)	2.4 (0.2)
FM	NA	3762.4	4.4E3 (4.2E3)	3.5E3 (2.0E3)	3.2E3 (1.4E3)	1.5E3 (151.4)	3.3E3 (1.1E3)

*TA* toal antibody, *BA* binding antibody, *FM* free MMAE

Serum concentrations of FM were much lower than those of TA and BA following single dose at all does levels. Mean *C*_max_ and AUC_0−*t*_ of FA were 1–13 ng/mL and 118–2.4E3 h*ng/mL across the six dose levels, respectively, which were more than 4000- and 1000-fold lower than those of TA and BA, respectively (Table 4). There was an apparent delay for FA to reach its *C*_max_, with median *T*_max_ of approximately 2–3 day post-infusion. The mean terminal half-lives of FA were largely similar to those of TA and BA, with mean values ranging from 38.3 to 68.6 h across the six dose levels. For FM analyte, *C*_max_ and AUC_0−inf_ displayed similar dose-dependent manner within the dose range of 0.1–3.0 mg/kg. It is important to note that the significant increased systemic exposures (*C*_max_ was 13.5 ng/mL and AUC_0−inf_ was 2.0E3 h*ng/mL) and decreased half-life (38.3 h) of FM at 3.0 mg/kg dose level were observed, compared with those (5.8 ng/mL, 990 h*ng/mL and 61.3 h for *C*_max_ and AUC_0−*t*_, respectively) at 2.5 mg/kg dose levels. Additionally, the systemic exposures of FM was much lower than those of CM (Figure S1).

After multiple administration (Q2W) of RC48 at 2.5 mg/kg dose level, significantly elevated half-lives of TA(44.12 h), BA(103.05 h), and FM (81.26 h) were obtained, compared with those (36.75 h, 45.68 h, 61.26 h, respectively) of three analytes after single dose of RC48 at the same dose level. For both TA and BA, *C*_max_ decreased by 30% and AUC_0-t_ remained stable after multiple dose compared to those after single dose. For FM analyte, there were no remarkably changes for both *C*_max_ and AUC_0−inf_ between single and multiple doses (Table 4).

Anti-drug antibody (ADA) was assessed in 56 patients(one patient had no post-treatment serum sample available). Of these 56 patients, 11(19.6%) had ADA response, 8 of the 11 were from 2.0 mg/kg dose cohort. Comparison of PK parameters between ADA positive and negative patients revealed that impact on PK only occurred in the single-dose cohort. *C*_max_, AUC_0−*t*_, and AUC_0−∞_ of free MMAE in ADA-negative and positive patients were 5.81 ± 4.21 vs. 2.86 ± 0.83 (ng/mL), 820.83 ± 548.25 vs. 415.11 ± 125.33 (h*ng/mL), and 947.99 ± 701.28 vs. 430.94 ± 121.89 (h*ng/mL), respectively. Details about ADA and PK are shown in supplementary resources.

## Discussion

Due to complex heterogeneity of HER2 expression in gastric cancer, there is no standard of care established for HER2 positive gastric cancer that has progressed after first-line treatment of trastuzumab. Antibody–drug conjugate (ADC) is one of the emerging treatment strategies in HER2-positive tumors in recent years. In May 2020, DS-8201, a HER2-directed antibody and topoisomerase inhibitor conjugate, received Breakthrough Therapy Designation (BTD) from the U.S. FDA for the treatment of patients with HER2 positive unresectable or metastatic gastric or GEJ adenocarcinoma who have received two or more prior regimens including trastuzumab based on the results of DESTINY-Gastric01 clinical trial [17]. In our current phase I study, RC48 also demonstrated a manageable safety profile and promising antitumor activity with HER2-low expression (IHC 2+/FISH−) gastric cancer patients also benefiting from the treatment. If the efficacy is confirmed in subsequent trials, RC48 might benefit more patients with HER2-positive gastric cancer.

One of the purposes of the study was to conduct integrated analysis to characterize the PK and explore the exposure–response relationship of RC48. The PK characteristic of TA and BA showed that dose-proportional increases both in exposures and half-lives at doses of 0.1–3.0 mg/kg. The pharmacokinetic profiles of two analytes were similar, which may indicate that the ADC drug would be stable in serum. Furthermore, no evidences of accumulation in serum for TA and BA were observed after repeated dosing of ADC drug at 2.5 mg/kg Q2W.

The PK characteristic of FM was also studied. The significant lower *C*_max_ and AUC_0−inf_ of FM compared to those of CM demonstrated that the majority of MMAE was conjugated with antibody. It was supposed that FA was released slowly from the ADC drug after the process of cellular uptake, lysosomal internalization and degradation. The apparent delay of FA in reaching its *C*_max_ compared with CM also confirms the suppose as time is needed for the whole process. In addition, accumulation of FA in serum was not observed after multiple doses of ADC drug at 2.5 mg/kg Q2W.

The safety profile of RC48 was consistent with what would be expected of a HER2-targeted antibody–drug conjugate that uses monomethyl auristatin E as its payload [10]. The most common classes of treatment-related adverse events were gastrointestinal disorders, hematological toxicities, and nerve toxicities. The incidences of ≥ grade 3 AEs (59.6% vs. 60%), SAEs (29.8% vs. 29%), and death (7.0% vs. 4.0%) were similar to those of the similar drug T-DM1 [6]. Safety data indicated that patients with advanced gastric cancer had similar tolerability to 2.0 mg/kg Q2W and 2.5 mg/kg Q2W. Additionally, clinical efficacy increased in a dose-dependent manner. On the basis of the balance between PK, activity and safety, 2.5 mg/kg Q2W was chosen as the RP2D for further investigation. However, dose expansion was completed only in the 2.0 mg/kg Q2W cohort in this phase I study, and the sample size in the 2.5 mg/kg Q2W cohort was rather small (*N* = 11); thus, the efficacy of RC48 in the 2.5 mg/kg Q2W dose needs further validation in phase II trials.

Based on previous studies, interstitial lung disease, also referred to as pneumonitis, is a rare but shared adverse event in several anti-HER2 drugs, including trastuzumab(9.9%), lapatinib (0.2%), T-DM1 (0.5%), DS-8201 (9.0%), and trastuzumab duocarmazine (7.7%) [17, 18]. However, no treatment-related lung damage was reported in our present phase I trial of RC48. Additionally, no pulmonary toxicities occurred in phase II trials [19]. For this adverse event, RC48 seems to have advantage over other anti-HER2 drugs, including DS-8201. Since treatment-related lung damage is a known complication of HER2-targeted antibody and cytotoxic chemotherapy, and the two drug elements in ADC were given concurrently, it is difficult to be sure of the offensive drug. The antibody used in RC48 (hertuzumab) has a higher affinity than trastuzumab. Also, the payload cytotoxic agent (MMAE) was different from DS-8201. However, specific mechanism remains unclear and it is difficult to draw a firm conclusion due to the limited sample size. The pulmonary toxicities of RC48 will continue to be further observed in the future studies. Another notable adverse event was the high incidence of treatment-related death. All of the three treatment-related deaths were considered to be due to neutropenia and severe infection, indicating hematological toxicity of RC48 should be monitored closely and controlled timely in clinical practice.

One of the significant findings of this study is that RC48 might be effective in HER2 low expressed gastric cancers. In current clinical practice, these patients are classified and treated according to guidelines for HER2 negative gastric cancer. Researchers have found that over 40–60% percent of all gastric cancers express low levels of HER2 as IHC 2+/FISH− [20], but no HER2-targeted therapies are currently approved for this population. Notably, in the current study, HER2 IHC2+/FISH− patients responded similarly to IHC2+/FISH+ and IHC3+ patients, with 72.7%, 60.0%, and 52.6% patients obtained tumor shrinkage, respectively. It makes RC48 an attractive agent for further study in HER2 positive gastric cancer, with the potential to expand the targeted population of interest due to activity in even low HER2 expressing tumors.

This promising efficacy may be due to the unique characteristics of RC48 that differentiates it from other HER2-targeted drugs. These characteristics include Hertuzumab which had a higher affinity to HER2 than trastuzumab, and the VC linker which was designed for optimal stability in human plasma and efficient cleavage by human cathepsin B [13]. Therefore, unlike T-DM1 with minimal bystander effect on nearby cells due to poor membrane permeability, RC48 has a bystander effect which can reverse T-DM1 resistance by acting on populations of cells not overexpressing HER2 [16, 21]. Despite the failure of T-DM1 in gastric cancer, several other antibody–drug conjugates are under investigation, varying in antibody and linker payload [10, 22, 23]. There are currently six HER2 directed ADCs in varying stages of clinical development in patients with HER2 overexpressing gastrointestinal malignancies [12]. These agents are being developed with the goal of improving the activity or the safety of HER2 targeted agents and also to enlarge the targeted population by developing agents that are effective in those with lower levels of HER2 expression. For instance, DS8201, an antibody–drug conjugate comprised of a humanized antibody against HER2, a novel enzyme-cleavable linker, and a topoisomerase I inhibitor payload also showed efficacy in pre-treated metastatic HER2-positive patients, including HER2 low expressed cancers, indicating such a “bystander” effect may even be substantial enough to induce responses in HER2 ICH 1+ tumors [22, 24]. Similar to DS8201, RC48 can thus be a promising agent whose mechanisms of action may overcome resistance accounted for intratumoral heterogeneity of HER2 overexpression and outgrowth of HER2-negative clones.

The researchers also saw signs of efficacy in the patients with other cancer types: PR occurred in 50% of patients with urothelial cancer, and DCR reached 100%. Phase II clinical trial is currently ongoing, and preliminary result has demonstrated a clinically meaningful ORR of 60.5% in pretreated HER-2 positive metastatic urothelial cancer patients including those who underwent failure to the immunotherapy [25].

There are some limitations to this study. On the one hand, pretreatment biopsy was not mandatory when patients were enrolled. However, differences in HER2 expression before and after trastuzumab were reported in HER2-positive gastric cancer and it is related with efficacy of trastuzumab in cross-line therapy [2, 3].Therefore, it is imperative to update HER2 status just before treatment. On the other hand, HER2 status was defined based on detection methods of IHC and FISH. With the development of next-generation sequencing and liquid biopsy, circulating tumor DNA sequencing has provided novel insights into HER2 gene alterations, and overcame heterogeneity of HER2 expression to some degree [26]. Thus, further optimization of detection methods might be required to identify patients that could benefit from treatment with RC48.

In summary, in this phase I study, RC48 showed preliminary anti-tumor activity and a manageable safety profile in patients with advanced HER2-positive gastric cancer, including those with HER2 2+/FISH−, and those pre-treated with HER2-targeted drugs. Findings from this study remain to be confirmed in further investigation. Phase II studies have been initiated to confirm these promising findings in HER2-positive gastric cancer (NCT03556345) and urothelial cancer patients (NCT03809013).

## Conclusion

RC48 is associated with mild toxicity and substantial clinical activity in HER2 positive solid tumors, particularly offering potential efficacy in HER2 low expression gastric cancers. Phase II trials in patients with advanced HER2-positive gastric cancer and urothelial cancer have been initiated to confirm these promising findings.

## Supplementary Information

Below is the link to the electronic supplementary material.

Supplementary file1 (PDF 157 KB)

Supplementary file2 (PDF 143 KB)

Supplementary file3 (PDF 1193 KB)

Supplementary file4 (PDF 374 KB)

Supplementary file5 (PDF 601 KB)

Supplementary file6 (PDF 105 KB)

Supplementary file7 (PDF 132 KB)

Supplementary file8 (DOCX 287 KB)
