# Supplementary material for: Phase I study of the recombinant humanized anti-HER2 monoclonal antibody–MMAE conjugate RC48-ADC in patients with HER2-positive advanced solid tumors
Source: Gastric Cancer. 2021 May 4;24(4):913–25. doi: 10.1007/s10120-021-01168-7 (PMC8205919; doi:10.1007/s10120-021-01168-7)

Figure S1. Mean logarithmic concentration vs. time plot of total antibody (TA, A), binding antidody (BA, B), free MMAE (FM, C) and conjugated MMAE(CM, D) following infusion of RC48 at different dose level.

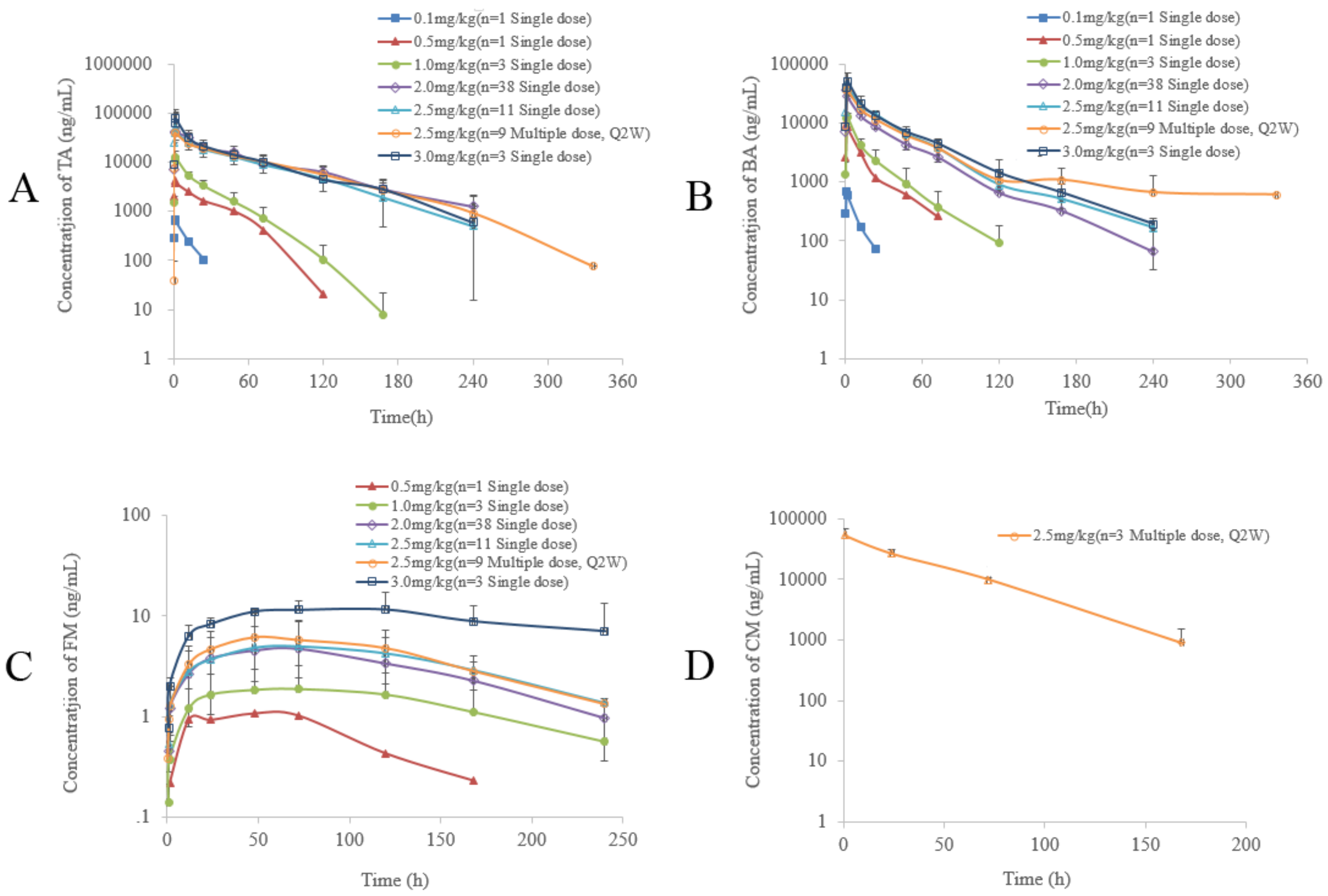

Supplement: Supplementary file 1 — Supplementary file1 (PDF 157 KB) [file 10120_2021_1168_MOESM1_ESM.pdf]
