# Supplementary figures and images for: Phase I study of the recombinant humanized anti-HER2 monoclonal antibody–MMAE conjugate RC48-ADC in patients with HER2-positive advanced solid tumors
Source: Gastric Cancer. 2021 May 4;24(4):913–25. doi: 10.1007/s10120-021-01168-7 (PMC8205919; doi:10.1007/s10120-021-01168-7)

Figure S2. Duration of treatment by patient

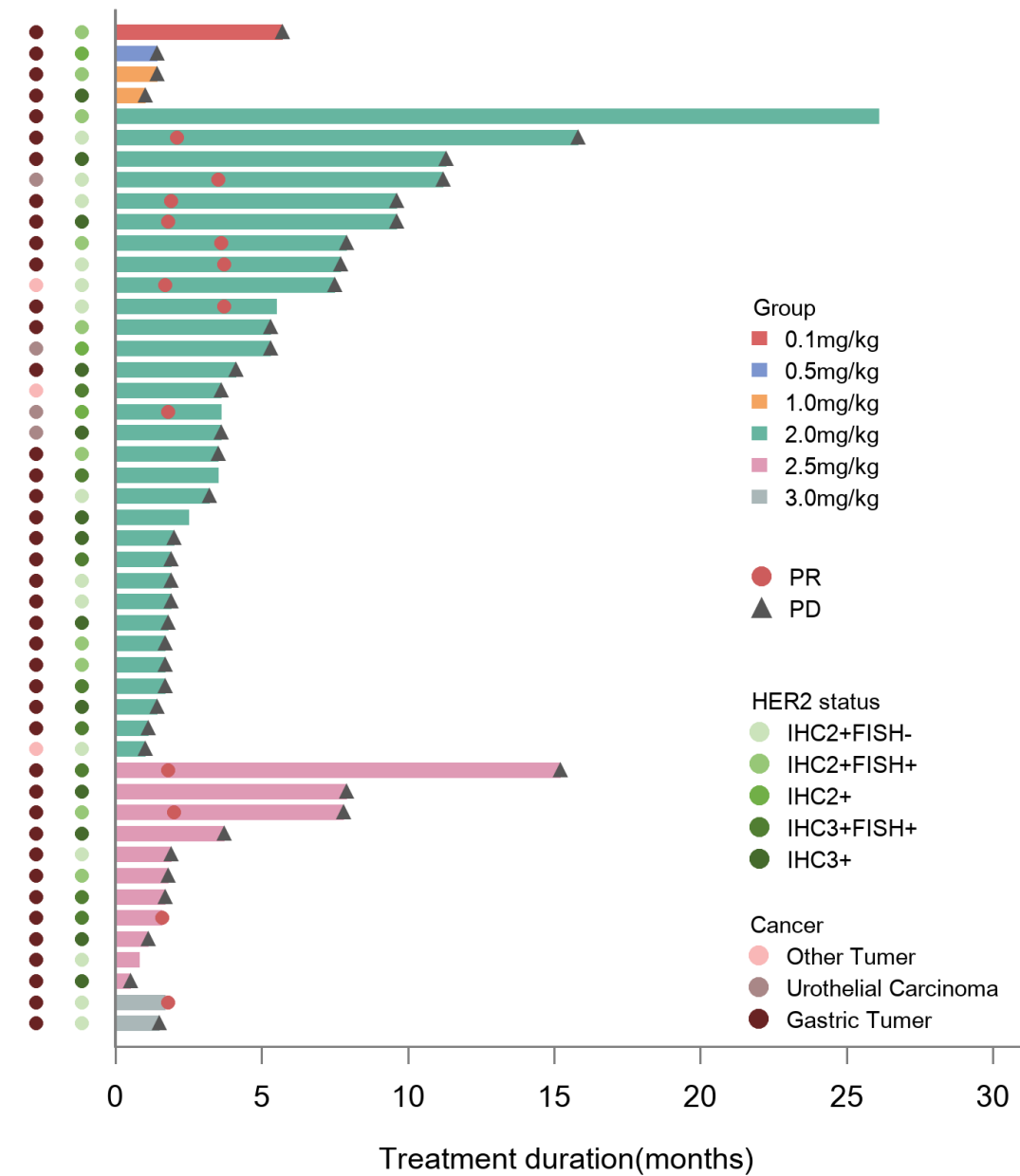

Supplement: Supplementary file 2 — Supplementary file2 (PDF 143 KB) [file 10120_2021_1168_MOESM2_ESM.pdf]
