# Supplementary material for: Phase I study of the recombinant humanized anti-HER2 monoclonal antibody–MMAE conjugate RC48-ADC in patients with HER2-positive advanced solid tumors
Source: Gastric Cancer. 2021 May 4;24(4):913–25. doi: 10.1007/s10120-021-01168-7 (PMC8205919; doi:10.1007/s10120-021-01168-7)
Supplement: Supplementary file 3 — Supplementary file3 (PDF 1193 KB) [file 10120_2021_1168_MOESM3_ESM.pdf]

北京肿瘤医院医学伦理委员会

伦理审查意见

意见号	2015YW42		
项目名称	注射用重组人源化抗 HER2 单抗-MMAE 偶联剂(简称:RC48-ADC)治疗 HER2 阳性晚期恶性实体肿瘤的安全性，耐受性和药代动力学的开放、剂量递增的 I 期临床研究（方案编号：C002 CANCER）		
项目负责人	沈琳		
项目负责人所在科室	北京肿瘤医院消化肿瘤内科		
申办方	烟台荣昌生物工程有限公司		
审查类别	药物	审查方式	会议审查
审查日期	2015. 10. 26	审查地点	科研楼四楼会议室
评审委员	郝纯毅 赵军 吴晓江 谢彦 王天峰 张艳华 白月玲 纪磊 姜炳强 文贤子 贾淑芹 韩淑燕 张雷 高静		
审查文件	详见目录清单		

审查意见

依据《中华人民共和国执业医师法》、《医疗机构管理条例》、《药物临床试验质量管理规范》，《涉及人体的生物医学研究伦理审查办法（试行）》、《药物临床试验伦理审查工作指导原则》、世界医学会《赫尔辛基宣言》、世界卫生组织《生物医学研究审查伦理委员会操作指南》、国际医学科学组织委员会《涉及人的生物医学研究国际伦理准则》等法律、法规和国际准则，伦理委员会于 2015 年 10 月 26 日对上述研究方案、知情同意书及有关内容进行了认真讨论并投票表决，结果如下：参会人数 14 人，投票人数 14 人，通过 0 票，修改后通过 14 票，修改后再议 0 票，未通过 0 票。审查结果决定为**修改后通过**。伦理委员会认为知情同意书应当做进一步修改。

知情同意书修改意见：

1. 在药物不良反应中使用 HER2 单抗可能带来的副作用未告知，建议补充；
2. 方案中列举的研究可能发生的“骨髓与免疫抑制、肝脏毒性、消化道损伤、生殖毒性、皮肤毒性”在知情研究风险中未告知，建议补充；
3. 在获益部分，I 期临床研究首先应强调不获益，建议补充；
4. 受试者职责中建议告知受试者：哪些为禁用、可酌情使用、需谨慎使用的药物，并归纳需要详细告知医生您正在使用的药物，且强调在研究期间用药应当征得研究者同意；
5. 费用和补偿需分别列项，不要把补偿写在费用项，建议修改；

6. “如果得到可能影响受试者继续参加试验的信息，受试者或其合法代理人将及时得到通报，或需要签署新的知情同意书”未告知，建议补充；
7. 如受试者是 18 周岁以上具有完全民事行为能力的自然人，签字页中建议删除法定代理人。法定代理人有专门的法律规定。如果需要家属签字，可以写“委托代理人”，同时需要委托书，授权范围有较为严格要求，建议由受试者本人签字，不会写字的受试者由家属代签的，注明代签人，患者摁手印；
8. 建议购买保险。

请提交《复审申请》，回复上述修改意见，按审查意见修改后的文件、方案/知情同意书/招募广告请注明新的版本号和版本日期，并以阴影和/或下划线方式标注修改部分，报伦理委员会审查。逾期 3 个月未提交复审者，将作为自动撤销本项目的审查申请。

附：目录清单：

1. 伦理审查申请表
2. CFDA 临床试验批件(批件号：2015L02291；日期：2015 年 9 月 22 日)
3. 药检报告
4. 临床试验方案(版本号：2.0；版本日期：2015 年 9 月 21 日)
5. 知情同意书(版本号：1.0；版本日期：2015 年 9 月 21 日)
6. 病例报告表(版本号：1.0；版本日期：2015 年 9 月 27 日)
7. 研究者手册(版本号：1.0；版本日期：2014 年 8 月 25 日)
8. 本中心主要研究者简历、GCP 证书复印件
9. 申办者资质证明
10. GMP 声明

北京肿瘤医院医学伦理委员会

主任委员（签名）：[illegible]

日期：2015.10.26

北京肿瘤医院医学伦理委员会
签到表及保密协议

Ethics Committee of Beijing Cancer Hospital
Attendance List & Confidential Agreement

作为北京肿瘤医院医学伦理委员会成员，我将对我所审阅的有关资料以及伦理委员会会议的内容保密。

As an Ethics Committee member of Beijing Cancer Hospital, I agree to hold the information of the protocol above and its relevant materials as a confidential manner.

职务	姓名	签名	日期
伦理委员会副主任 Signature	郝纯毅	郝纯毅	2015.10.26
伦理委员会副主任 Signature	吴晓江	吴晓江	2015.10.26
伦理委员会副主任 Signature	王天峰	王天峰	2015.10.26
伦理委员会副主任 Signature	赵军	赵军	2015.10.26
伦理委员会成员 Signature	谢彦	谢彦	2015-10-26
伦理委员会成员 Signature	张艳华	张艳华	2015.10.26
伦理委员会成员 Signature	白月玲	白月玲	2015.10.26
伦理委员会成员 Signature	文贤子	文贤子	2015.10.26
伦理委员会成员 Signature	贾淑芹	贾淑芹	2015.10.26
伦理委员会成员 Signature	韩淑燕	韩淑燕	2015.10.26

伦理委员会成员 Signature	高静	高静	2015.10.26
伦理委员会成员 Signature	张雷	[illegible]	2015.10.26
伦理委员会成员 Signature	纪磊	[illegible]	2015.10.26
伦理委员会成员 Signature	姜炳强	[illegible]	2015.10.26

北京肿瘤医院医学伦理委员会
名单及组成情况
Ethics Committee of Beijing Cancer Hospital
Member List

Name<br>姓名	Gender<br>性别	Unit/Title<br>工作单位和技术职称	Title in the EC<br>委员会职务
Hao Chunyi<br>郝纯毅	Male<br>男	Dept. of Hepatic Biliary&Pancreatic surgery, Beijing Cancer Hospital/ Professor<br>本院肝胆胰外科主任医师	Vice Chairman<br>副主任
Zhao Jun<br>赵军	Male<br>男	Dept. of Thoracic Oncology, Beijing Cancer Hospital/ Associate Professor<br>本院胸部肿瘤内科副主任医师	Member<br>委员
WangTianfeng<br>王天峰	Male<br>男	Dept. of Breast Cancer Center, Beijing Cancer Hospital/Associate Professor<br>本院乳腺中心副主任医师	Member<br>委员
Wu Xiaojiang<br>吴晓江	Male<br>男	Dept of Gastrointestinal Surgery, Beijing Cancer Hospital/Associate Professor<br>本院胃肠外科副主任医师	Member<br>委员
Xie Yan<br>谢彦	Female<br>女	Dept. of Lymphoma, Beijing Cancer Hospital/ Associate Professor<br>本院淋巴肿瘤内科副主任医师	Member<br>委员
Bai Yueling<br>白月玲	Female<br>女	Dept. of Nursing, Beijing Cancer Hospital/Associate Professor Of Nursing<br>本院护理部副主任护师	Member<br>委员
Zhang Yanhua<br>张艳华	Female<br>女	Dept of Pharmacy, Beijing Cancer Hospital/Chief Pharmacist<br>本院药剂科主任药师	Member<br>委员
Han Shuyan<br>韩淑燕	Female<br>女	Dept. of Integration of Chinese and Western Medicine, Beijing Cancer Hospital/ Associate Professor<br>本院中西医结合暨老年肿瘤科副研究员	Member<br>委员
ZhangLei<br>张雷	Male<br>男	Dept. of Epidemiological Research, Beijing Cancer Hospital/Associate Professor<br>本院流行病学研究室助理研究员	Member<br>委员
WenXianZi<br>文贤子	Female<br>女	Dept. of Gastric cancer laboratory, Beijing Cancer Hospital/Associate Professor<br>本院胃癌实验室副研究员	Member<br>委员
Gao Jing<br>高静	Female<br>女	Dept. of GI Oncology, Beijing Cancer Hospital/ Associate Professor<br>本院消化肿瘤内科副教授	Member<br>委员
JiaShuQin<br>贾淑芹	Female<br>女	Laboratory of Molecular oncology, Beijing Cancer Hospital/Associate Professor<br>本院分子肿瘤学研究室副研究员	Member<br>委员

Ji Lei<br>纪磊	Female<br>女	Beijing Hua Wei Law Office/<br>Lawyer<br>北京华卫律师事务所律师	Member<br>委员
JiangBingqiang<br>姜炳强	Male<br>男	Air Force Command College/Teacher<br>空军指挥学院教员	Member<br>委员

声明：北京肿瘤医院伦理委员会的组成符合ICH国际规范和中国GCP规范，并遵守上述规范行使其职责和权利。

Statement:

The Ethics Committee of Beijing Cancer Hospital is constituted and functions in accordance with the "ICH-GCP "and "China GCP Guidelines" explaned in the "Drug Administration Regulations of People's Republic of China".
