# Supplementary material for: Phase I study of the recombinant humanized anti-HER2 monoclonal antibody–MMAE conjugate RC48-ADC in patients with HER2-positive advanced solid tumors
Source: Gastric Cancer. 2021 May 4;24(4):913–25. doi: 10.1007/s10120-021-01168-7 (PMC8205919; doi:10.1007/s10120-021-01168-7)
Supplement: Supplementary file 4 — Supplementary file4 (PDF 374 KB) [file 10120_2021_1168_MOESM4_ESM.pdf]

北京肿瘤医院医学伦理委员会

伦理审查批件

批件号	2015YW42		
项目名称	注射用重组人源化抗HER2单抗-MMAE偶联剂(简称:RC48-ADC)治疗HER2阳性晚期恶性实体肿瘤的安全性，耐受性和药代动力学的开放、剂量递增的I期临床研究（方案编号：C002 CANCER)		
项目负责人	沈琳		
项目负责人科室	北京肿瘤医院消化肿瘤内科		
申办方	烟台荣昌生物工程有限公司		
审查类别	药物	审查方式	会议审查
审查日期	2015.10.26	审查结果	修改后通过
复审审查日期	2015.11.12	审查方式	快速审查
审查文件	详见目录清单		

审查意见

依据《中华人民共和国执业医师法》、《医疗机构管理条例》、《药物临床试验质量管理规范》,《涉及人体的生物医学研究伦理审查办法（试行)》、《药物临床试验伦理审查工作指导原则》、世界医学会《赫尔辛基宣言 》、世界卫生组织《生物医学研究审查伦理委员会操作指南》、国际医学科学组织委员会《涉及人的生物医学研究国际伦理准则》等法律、法规和国际准则,伦理委员会于2015年11月12日对修改后的知情同意书及相关内容进行了审阅，认为基本符合伦理要求，可以开始临床研究。

请遵循伦理委员会批准的方案开展研究，保护受试者的健康与权益。

1. 研究过程中若变更项目负责人，对研究方案、知情同意书、病例报告表、招募材料等的任何修改，请提交修正案审查申请；
2. 发生严重不良事件，请在获知后的24小时内提交严重不良事件报告；
3. 研究者没有遵从方案开展研究，可能对受试者的权益/健康、以及研究的科学性造成不良影响，请提交违规事件报告；
4. 申请人暂停或提前终止临床研究，请及时提交暂停/中止研究报告；
5. 研究结束时，请提交结题报告。
6. 本批件自批准之日起半年内有效，请至少在批件失效日期前1个月内提交研究进展报告。

附：目录清单：

1. 伦理审查申请表
2. CFDA临床试验批件(批件号：2015L02291；日期：2015年9月22日)
3. 药检报告
4. 临床试验方案(版本号：2.0；版本日期：2015年9月21日)
5. 知情同意书（版本号：2.0； 版本日期：2015年11月4日 ）
6. 病例报告表(版本号：1.0；版本日期：2015年9月27日)

7. 研究者手册(版本号：1.0；版本日期：2014年8月25日)
8. 本中心主要研究者简历、GCP证书复印件
9. 申办者资质证明
10. GMP声明

批准日期	2015.11.12	批件失效期	2016.05.12
主任签字	[signature]	签字日期	2015.11.12

北京肿瘤医院医学伦理委员会

地址：北京市海淀区阜成路52号
邮编：100142
电话：0086-10-88196391

No. 52 Fu-cheng Road, Haidian District, Beijing
Beijing 100142, P.R. China
Tel: 0086-10-88196391
