# Supplementary material for: Phase I study of the recombinant humanized anti-HER2 monoclonal antibody–MMAE conjugate RC48-ADC in patients with HER2-positive advanced solid tumors
Source: Gastric Cancer. 2021 May 4;24(4):913–25. doi: 10.1007/s10120-021-01168-7 (PMC8205919; doi:10.1007/s10120-021-01168-7)
Supplement: Supplementary file 5 — Supplementary file5 (PDF 601 KB) [file 10120_2021_1168_MOESM5_ESM.pdf]

# Certification for medical or scientific Journal editor from Ethics Committee of Beijing Cancer Hospital

To editor of ___Gastric Cancer___

This clinical research report titled **"Phase I study of the recombinant humanized anti-HER2 monoclonal antibody-MMAE conjugate RC48-ADC in patients with HER2-positive advanced solid tumors"** (Ethics number: 2015YW42) was submitted by Professor Lin Shen. The investigation project and its Informed Consent Form have been examined and certified by Ethics Committee of Beijing Cancer Hospital on 12 (day) 11 (month) 2015 (year).

Ethics Committee of Beijing Cancer Hospital

7 (day) 12 (month) 2020 (year)

扫描全能王 创建
