# Supplementary material for: Phase I study of the recombinant humanized anti-HER2 monoclonal antibody–MMAE conjugate RC48-ADC in patients with HER2-positive advanced solid tumors
Source: Gastric Cancer. 2021 May 4;24(4):913–25. doi: 10.1007/s10120-021-01168-7 (PMC8205919; doi:10.1007/s10120-021-01168-7)
Supplement: Supplementary file 6 — Supplementary file6 (PDF 105 KB) [file 10120_2021_1168_MOESM6_ESM.pdf]

Supplementary table 1. Analysis on the incidence of AE and DLT in DLT observation period (day1-21)

	0.1mg/kg<br>N=1	0.5mg/kg<br>N=1	1.0mg/kg Q2W<br>N=3	2.0mg/kg Q2W<br>N=35	2.0mg/kg Q3W<br>N=3	2.5mg/kg Q2W<br>N=11	3.0mg/kg Q2W<br>N=3	Total<br>N=57
AE	1(100.0)	1(100.0)	3(100.0)	32(91.4)	3(100)	11(100.0)	3(100.0)	54(94.7)
DLT	0(0)	0(0)	0(0)	0(0)	0(0)	2(18.2)	1(33.3)	3(12.0)
