# Supplementary material for: Phase I study of the recombinant humanized anti-HER2 monoclonal antibody–MMAE conjugate RC48-ADC in patients with HER2-positive advanced solid tumors
Source: Gastric Cancer. 2021 May 4;24(4):913–25. doi: 10.1007/s10120-021-01168-7 (PMC8205919; doi:10.1007/s10120-021-01168-7)
Supplement: Supplementary file 7 — Supplementary file7 (PDF 132 KB) [file 10120_2021_1168_MOESM7_ESM.pdf]

Supplementary table 2. Efficacy in gastric cancer subgroup according to previous HER2-targeted therapy.

	0.1mg/kg N =1(%)	0.5mg/kg N =1(%)	1.0mg/kg Q2W N=2 (%)	2.0mg/kg Q2W N=26 (%)	2.0mg/kg Q3W N =3(%)	2.5mg/kg Q2W N =11(%)	3.0mg/kg Q2W N =3(%)	In total N=47(%)
Not received HER2-targeting therapies before	1	0	1	16	2	4	3	27
CR	0	0	0	0	0	0	0	0
PR	0	0	0	4(25.0)	1(50.0)	1(25.0)	0	6(22.2)
Unconfirmed PR	0	0	0	0	0	0	1(33.2)	1(3.7)
SD	1(100)	0	0	3(18.75)	1(50.0)	1(25.0)	0	6(22.2)
PD	0	0	1(100)	5(31.25)	0	2(50.0)	1(33.3)	9(33.3)
ORR	0	0	0	4(25.0)	1(50.0)	1(25.0)	1(33.3)	7(25.9)
DCR	1(100)	0	0	7(43.75)	2(100.0)	2(50.0)	1(33.3)	13(48.1)
Received HER2-targeting therapies before	0	1	1	10	1	7	0	20
CR	0	0	0	0	0	0	0	0
PR	0	0	0	0	1(100.0)	1(14.3)	0	2(10.0)
Unconfirmed PR	0	0	0	0	0	1(14.3)	0	1(5.0)
SD	0	0	0	4(40.0)	0	2(28.6)	0	6(30.0)
PD	0	1(100)	1(100)	5(50.0)	0	3(42.9)	0	10(50.0)
ORR	0	0	0	0	1(100.0)	2(28.6)	0	3(15.0)
DCR	0	0	0	4(40.0)	1(100.0)	4(57.1)	0	9(45.0)
