# Supplementary material for: Phase I study of the recombinant humanized anti-HER2 monoclonal antibody–MMAE conjugate RC48-ADC in patients with HER2-positive advanced solid tumors
Source: Gastric Cancer. 2021 May 4;24(4):913–25. doi: 10.1007/s10120-021-01168-7 (PMC8205919; doi:10.1007/s10120-021-01168-7)
Supplement: Supplementary file 8 — Supplementary file8 (DOCX 287 KB) [file 10120_2021_1168_MOESM8_ESM.docx]

**A Phase I, Open-Label, Dose-Escalation** **Clinical Trial to Assess Safety, Tolerability and Pharmacokinetics of**

**Recombinant Humanized Anti-HER2 Monoclonal Antibody-MMAE Conjugate for Injection (Abbreviated as RC48-ADC) in Treatment of**

**HER2-Positive Advanced Malignant Solid Tumors**

**Protocol of Clinical Trial**

**Confidentiality Statement**

**All data and information in this document are proprietary to RemeGen., Ltd. and shall not be disclosed unless such disclosure is required by current laws or regulations. These materials shall not be disclosed to anyone other than involved investigators, who are allowed to review the data and information on the premise of ensuring confidentiality.**

| **Sponsor:** | **RemeGen., Ltd.** |
| --- | --- |
| **Responsible Research Organization:** | **Beijing Cancer Hospital** |
| **Protocol Designer:** | **Shen Lin** |
| **Protocol Development Date:** | **July 4, 2017** |
| **Version:** | **6.0** |
| **Protocol Number:** | **C002 CANCER** |

**Statement and Signature**

Investigational drug: Recombinant Humanized Anti-HER2 Monoclonal Antibody-MMAE Conjugate for Injection

Study title: A Phase I, Open-Label, Dose-Escalation Clinical Trial to Assess Safety, Tolerability and Pharmacokinetics of Recombinant Humanized Anti-HER2 Monoclonal Antibody-MMAE Conjugate for Injection (Abbreviated as RC48-ADC) in Treatment of HER2-Positive Advanced Malignant Solid Tumors

Protocol Number: **C002 CANCER**

**Statement of Investigator**

I have read and understood the content of this protocol. I promise to fulfill the responsibilities of an investigator in compliance with Good Clinical Practice (GCP), carry out this clinical trial in accordance with this protocol, and provide details about related data, regulations and responsibilities to the personnel participating in this study.

I will strictly abide by the Declaration of Helsinki and explain to the subjects the details of this clinical study approved by the ethics committee, and obtain informed consents. I will be responsible for making medical decisions related to this clinical study to ensure that subjects can receive appropriate treatment when adverse events occur during the trial. When any serious adverse events occur during the trial, I will immediately take appropriate therapeutic measures to protect the safety of the subject and report to relevant institutions specified in the GCP.

I agree to accept monitoring and audits by the monitors or auditors dispatched by the sponsor, as well as inspections by drug regulatory agencies to ensure the quality of the clinical trial.

I agree to keep all relevant data received or obtained in this study confidential.

﹎﹎﹎﹎﹎﹎﹎﹎﹎﹎﹎﹎﹎﹎﹎﹎﹎﹎﹎﹎﹎﹎﹎﹎﹎﹎﹎﹎﹎﹎﹎﹎﹎﹎﹎﹎﹎

Responsible Research Organization: Beijing Cancer Hospital

Principal Investigator (Signature): Date:

﹎﹎﹎﹎﹎﹎﹎﹎﹎﹎﹎﹎﹎﹎﹎﹎﹎﹎﹎﹎﹎﹎﹎﹎﹎﹎﹎﹎﹎﹎﹎﹎﹎﹎﹎﹎﹎

**Statement and Signature**

Investigational drug: Recombinant Humanized Anti-HER2 Monoclonal Antibody-MMAE Conjugate for Injection

Study title: A Phase I, Open-Label, Dose-Escalation Clinical Trial to Assess Safety, Tolerability and Pharmacokinetics of Recombinant Humanized Anti-HER2 Monoclonal Antibody-MMAE Conjugate for Injection (Abbreviated as RC48-ADC) in Treatment of HER2-Positive Advanced Malignant Solid Tumors

Protocol Number: **C002 CANCER**

**Statement of Investigator**

I have read and understood the content of this protocol. I promise to fulfill the responsibilities of an investigator in compliance with Good Clinical Practice (GCP), carry out this clinical trial in accordance with this protocol, and provide details about related data, regulations and responsibilities to the personnel participating in this study.

I will strictly abide by the Declaration of Helsinki and explain to the subjects the details of this clinical study approved by the ethics committee, and obtain informed consents. I will be responsible for making medical decisions related to this clinical study to ensure that subjects can receive appropriate treatment when adverse events occur during the trial. When any serious adverse events occur during the trial, I will immediately take appropriate therapeutic measures to protect the safety of the subject and report to relevant institutions specified in the GCP.

I agree to accept monitoring and audits by the monitors or auditors dispatched by the sponsor, as well as inspections by drug regulatory agencies to ensure the quality of the clinical trial.

I agree to keep all relevant data received or obtained in this study confidential.

﹎﹎﹎﹎﹎﹎﹎﹎﹎﹎﹎﹎﹎﹎﹎﹎﹎﹎﹎﹎﹎﹎﹎﹎﹎﹎﹎﹎﹎﹎﹎﹎﹎﹎﹎﹎﹎

Participating Research Organization:

Principal Investigator of the Site (Signature): Date:

﹎﹎﹎﹎﹎﹎﹎﹎﹎﹎﹎﹎﹎﹎﹎﹎﹎﹎﹎﹎﹎﹎﹎﹎﹎﹎﹎﹎﹎﹎﹎﹎﹎﹎﹎﹎﹎

**Statement and Signature**

Investigational drug: Recombinant Humanized Anti-HER2 Monoclonal Antibody-MMAE Conjugate for Injection

Study title: A Phase I, Open-Label, Dose-Escalation Clinical Trial to Assess Safety, Tolerability and Pharmacokinetics of Recombinant Humanized Anti-HER2 Monoclonal Antibody-MMAE Conjugate for Injection (Abbreviated as RC48-ADC) in Treatment of HER2-Positive Advanced Malignant Solid Tumors

Protocol Number: **C002 CANCER**

**Statement of Investigator**

I have read and understood the content of this protocol. I promise to fulfill the responsibilities of an investigator in compliance with Good Clinical Practice (GCP), carry out this clinical trial in accordance with this protocol, and provide details about related data, regulations and responsibilities to the personnel participating in this study.

I will strictly abide by the Declaration of Helsinki and explain to the subjects the details of this clinical study approved by the ethics committee, and obtain informed consents. I will be responsible for making medical decisions related to this clinical study to ensure that subjects can receive appropriate treatment when adverse events occur during the trial. When any serious adverse events occur during the trial, I will immediately take appropriate therapeutic measures to protect the safety of the subject and report to relevant institutions specified in the GCP.

I agree to accept monitoring and audits by the monitors or auditors dispatched by the sponsor, as well as inspections by drug regulatory agencies to ensure the quality of the clinical trial.

I agree to keep all relevant data received or obtained in this study confidential.

﹎﹎﹎﹎﹎﹎﹎﹎﹎﹎﹎﹎﹎﹎﹎﹎﹎﹎﹎﹎﹎﹎﹎﹎﹎﹎﹎﹎﹎﹎﹎﹎﹎﹎﹎﹎﹎

Participating Research Organization:

Principal Investigator of the Site (Signature): Date:

﹎﹎﹎﹎﹎﹎﹎﹎﹎﹎﹎﹎﹎﹎﹎﹎﹎﹎﹎﹎﹎﹎﹎﹎﹎﹎﹎﹎﹎﹎﹎﹎﹎﹎﹎﹎﹎

**Statement and Signature**

Investigational drug: Recombinant Humanized Anti-HER2 Monoclonal Antibody-MMAE Conjugate for Injection

Study title: A Phase I, Open-Label, Dose-Escalation Clinical Trial to Assess Safety, Tolerability and Pharmacokinetics of Recombinant Humanized Anti-HER2 Monoclonal Antibody-MMAE Conjugate for Injection (Abbreviated as RC48-ADC) in Treatment of HER2-Positive Advanced Malignant Solid Tumors

Protocol Number: **C002 CANCER**

**Statement of Investigator**

I have read and understood the content of this protocol. I promise to fulfill the responsibilities of an investigator in compliance with Good Clinical Practice (GCP), carry out this clinical trial in accordance with this protocol, and provide details about related data, regulations and responsibilities to the personnel participating in this study.

I will strictly abide by the Declaration of Helsinki and explain to the subjects the details of this clinical study approved by the ethics committee, and obtain informed consents. I will be responsible for making medical decisions related to this clinical study to ensure that subjects can receive appropriate treatment when adverse events occur during the trial. When any serious adverse events occur during the trial, I will immediately take appropriate therapeutic measures to protect the safety of the subject and report to relevant institutions specified in the GCP.

I agree to accept monitoring and audits by the monitors or auditors dispatched by the sponsor, as well as inspections by drug regulatory agencies to ensure the quality of the clinical trial.

I agree to keep all relevant data received or obtained in this study confidential.

﹎﹎﹎﹎﹎﹎﹎﹎﹎﹎﹎﹎﹎﹎﹎﹎﹎﹎﹎﹎﹎﹎﹎﹎﹎﹎﹎﹎﹎﹎﹎﹎﹎﹎﹎﹎﹎

Participating Research Organization:

Principal Investigator of the Site (Signature): Date:

﹎﹎﹎﹎﹎﹎﹎﹎﹎﹎﹎﹎﹎﹎﹎﹎﹎﹎﹎﹎﹎﹎﹎﹎﹎﹎﹎﹎﹎﹎﹎﹎﹎﹎﹎﹎﹎**Statement of Sponsor**

Our company will be responsible for initiating, applying, organizing, monitoring, auditing and funding this clinical trial in compliance with Good Clinical Practice (GCP). In particular, our company will provide subjects who have suffered trial-related damage or death with compensation for treatment, and provide the investigators with legal and economic guarantees.

﹎﹎﹎﹎﹎﹎﹎﹎﹎﹎﹎﹎﹎﹎﹎﹎﹎﹎﹎﹎﹎﹎﹎﹎﹎﹎﹎﹎﹎﹎﹎﹎﹎﹎﹎﹎﹎

RemeGen., Ltd.

Responsible Person (Signature): Date:

﹎﹎﹎﹎﹎﹎﹎﹎﹎﹎﹎﹎﹎﹎﹎﹎﹎﹎﹎﹎﹎﹎﹎﹎﹎﹎﹎﹎﹎﹎﹎﹎﹎﹎﹎﹎﹎

**Statement and Signature**

Investigational drug: Recombinant Humanized Anti-HER2 Monoclonal Antibody-MMAE Conjugate for Injection

Study title: A Phase I, Open-Label, Dose-Escalation Clinical Trial to Assess Safety, Tolerability and Pharmacokinetics of Recombinant Humanized Anti-HER2 Monoclonal Antibody-MMAE Conjugate for Injection (Abbreviated as RC48-ADC) in Treatment of HER2-Positive Advanced Malignant Solid Tumors

Protocol Number: **C002 CANCER**

**Statement**

I have read and confirmed this protocol (Protocol No.: C002 CANCER; Version: 6.0; Version Date: July 4, 2017). I agree to perform the related duties in accordance with relevant Chinese laws, the Declaration of Helsinki, the CFDA GCP and this study protocol.

I agree to keep all relevant data received or obtained in this study confidential.

﹎﹎﹎﹎﹎﹎﹎﹎﹎﹎﹎﹎﹎﹎﹎﹎﹎﹎﹎﹎﹎﹎﹎﹎﹎﹎﹎﹎﹎﹎﹎﹎﹎﹎﹎﹎﹎

Organization Responsible for Pharmacokinetic Study: United-Power Pharma Tech Co., Ltd

Principal Investigator (Signature): Date:

﹎﹎﹎﹎﹎﹎﹎﹎﹎﹎﹎﹎﹎﹎﹎﹎﹎﹎﹎﹎﹎﹎﹎﹎﹎﹎﹎﹎﹎﹎﹎﹎﹎﹎﹎﹎﹎**Statement and Signature**

Investigational drug: Recombinant Humanized Anti-HER2 Monoclonal Antibody-MMAE Conjugate for Injection

Study title: A Phase I, Open-Label, Dose-Escalation Clinical Trial to Assess Safety, Tolerability and Pharmacokinetics of Recombinant Humanized Anti-HER2 Monoclonal Antibody-MMAE Conjugate for Injection (Abbreviated as RC48-ADC) in Treatment of HER2-Positive Advanced Malignant Solid Tumors

Protocol Number: **C002 CANCER**

**Statement**

I have read and confirmed this protocol (Protocol No.: C002 CANCER; Version: 6.0; Version Date: July 4, 2017). I agree to perform the related duties in accordance with relevant Chinese laws, the Declaration of Helsinki, the CFDA GCP and this study protocol.

I agree to keep all relevant data received or obtained in this study confidential.

﹎﹎﹎﹎﹎﹎﹎﹎﹎﹎﹎﹎﹎﹎﹎﹎﹎﹎﹎﹎﹎﹎﹎﹎﹎﹎﹎﹎﹎﹎﹎﹎﹎﹎﹎﹎﹎

Organizations Responsible for Data Management and Statistical Analysis: Beijing Bozhiyin Technology Co., Ltd

Center for Drug Clinical Research, Shanghai University of Traditional Chinese Medicine

Principal Statistical Analyst (Signature): Date:

﹎﹎﹎﹎﹎﹎﹎﹎﹎﹎﹎﹎﹎﹎﹎﹎﹎﹎﹎﹎﹎﹎﹎﹎﹎﹎﹎﹎﹎﹎﹎﹎﹎﹎﹎﹎﹎

**Table of Contents**

[4 Criteria for Subject Selection 32](#_Toc515033766)

[4.1 Inclusion Criteria 32](#_Toc515033767)

[4.2 Exclusion Criteria 33](#_Toc515033768)

[4.3 Withdrawal Criteria 34](#_Toc515033769)

[4.4 Criteria for Termination of Trial 34](#_Toc515033770)

[5. Investigational Drug 35](#_Toc515033771)

[6. Blood Collection Times and Outcome Measures 40](#_Toc515033772)

[7. Outcome Measures 42](#_Toc515033773)

[8. Study Procedure 45](#_Toc515033774)

[8.1 Screening Period 45](#_Toc515033775)

[8.2 Trial Period 46](#_Toc515033776)

[8.3 Extended Trial 47](#_Toc515033777)

[8.4 Extended Trial 48](#_Toc515033778)

[8.5 Conclusion/Withdrawal 49](#_Toc515033779)

[9 Clinical Observation 49](#_Toc515033780)

[10 Concomitant Medication 50](#_Toc515033781)

[10.1 Unavailable Drugs During Study 50](#_Toc515033782)

[10.2 Drugs may be Conditionally Used During Study 50](#_Toc515033783)

[11 Recording, Identification and Handling of Adverse Effects 52](#_Toc515033784)

[11.1 Recording of Adverse Effects 53](#_Toc515033785)

[11.2 Identification of Adverse Events 54](#_Toc515033786)

[11.3 Possible Adverse Events and Rescue Measures 55](#_Toc515033787)

[12. End of Study 56](#_Toc515033788)

[13. Observation and Records 56](#_Toc515033789)

[13.1 Recording of Data 56](#_Toc515033790)

[13.2 Data Monitoring 57](#_Toc515033791)

[13.3 Data Archiving 57](#_Toc515033792)

[14. Data Management 57](#_Toc515033793)

[15. Statistical Analysis 59](#_Toc515033794)

[15.1 Analytical Dataset 59](#_Toc515033795)

[15.2 Safety Analysis 59](#_Toc515033796)

[15.3 Pharmacokinetic Analysis 60](#_Toc515033797)

[15.4 Efficacy Analysis 60](#_Toc515033798)

[15.5 Analysis Software and General Requirements 61](#_Toc515033799)

[16. Ethical Requirements 61](#_Toc515033800)

[16.1 Study documents should be approved by the ethics committee in advance 61](#_Toc515033801)

[16.2 Subjects must go through the informed consent process and sign informed consent forms before the clinical trials 61](#_Toc515033802)

[16.3 Occurrence of any AEs in the trial should be able to be effectively treated and followed up 62](#_Toc515033803)

[17. Usage and Publication of Information 62](#_Toc515033804)

[18. Content Added During the Trial 62](#_Toc515033805)

[19. References 64](#_Toc515033806)

[20 History of Revision 64](#_Toc515033807)

[Annex 1. ECOG Scale 65](#_Toc515033808)

[Appendix 2. Trial Flow Chart of Dose Escalation Phase - Q2W Administration 66](#_Toc515033809)

[Appendix 3. Trial Flow Chart of Dose Escalation Phase - Q3W Administration 67](#_Toc515033810)

[Appendix 4. Flow Chart of Extended Trial - Q2W Administration 68](#_Toc515033811)

[Appendix 5. Trial Flow Chart of Dose Escalation Phase - QW Administration 69](#_Toc515033812)

**Abbreviations**

| **Abbreviations** | **Description** |
| --- | --- |
| **ALB** | **Albumin** |
| **ALT** | **Alanine Aminotransferase** |
| **AST** | **Aspartate Aminotransferase** |
| **ALP** | **Alkaline Phosphatase** |
| **CK-MB** | **Myocardial Zymogram** |
| **Cr** | **Creatinine** |
| **CR** | **Complete Response** |
| **CRF** | **Case Report Form** |
| **CTCAE** | **NCI Common Toxicity Criteria for Adverse Events** |
| **BUN** | **Blood Urea Nitrogen** |
| **DBIL** | **Direct Bilirubin** |
| **dL** | **Deciliter** |
| **DLT** | **Dose-Limiting Toxicity** |
| **EC** | **Ethics Committee** |
| **FISH** | **Fluorescence in situ Hybridization** |
| **GCP** | **Good Clinical Practice** |
| **Glu** | **Glucose** |
| **GGT** | **Gamma Glutamyltransferase** |
| **d** | **Day** |
| **h** | **Hour** |
| **Hb** | **Hemoglobin** |
| **HR** | **Heart Rate** |
| **IB** | **Investigator's Brochure** |
| **IBIL** | **Indirect Bilirubin** |
| **INR** | **International Normalized Ratio** |
| **ITT** | **Intent-to-Treat** |
| **IU** | **International Unit** |
| **IV** | **Intravenous Injection (Drip)** |
| **kg** | **Kilogram** |
| **LDH** | **Lactate Dehydrogenase** |
| **MTD** | **Maximum Tolerated Dose** |
| **ng** | **Nanogram** |
| **ORR** | **Objective Response Rate** |
| **PFS** | **Progression-Free Survival** |
| **PLT** | **Platelet** |
| **PR** | **Partial Response** |
| **RBC** | **Red Blood Cell** |
| **SAE** | **Serious Adverse Event** |
| **SAP** | **Statistical Analysis Plan** |
| **SBP** | **Systolic Blood Pressure** |
| **TAb** | **Total Antibody** |
| **TBIL** | **Total Bilirubin** |
| **TP** | **Total Protein** |
| **ULN** | **Upper Limit of Normal** |
| **WBC** | **White Blood Cell** |
| **RC48-ADC or RC48** | **Recombinant Humanized Anti-HER2 Monoclonal Antibody-MMAE Conjugate for Injection** |
| **TDM-1** | **Ado-trastuzumab Emtansine** |
| **SGN35** | **Brentuximab Vedotin** |
| **ADC** | **Antibody Drug Conjugate** |
| **MMAE** | **Monomethyl auristatin E** |
| **AUC** | **Area Under Curve** |
| **ADCC** | **Antibody-Dependent Cell-Mediated Cytotoxicity** |

**Study Summary**

| **Sponsor** | RemeGen., Ltd. |
| --- | --- |
| **Protocol Number** | **C002 CANCER** |
| **Study Phase** | Phase I Clinical Trial |
| **Investigational Drug** | RC48-ADC |
| **Study Site** | Beijing Cancer Hospital |
| **Study Design** | An open-label, multi-center, dose-escalation design |
| **Intended Number of**  **Enrolled Cases** | 24-50 |
| **Principal Investigator** | Shen Lin |
| **Study Objectives** | **Primary objectives:** To determine the maximum tolerated dose (MTD) and safety of RC48-ADC in subjects with HER2-positive advanced malignant solid tumors, and to determine the recommended dose in the Phase II clinical trial.  **Secondary objectives:** To investigate the pharmacokinetic (PK) characteristics of RC48-ADC in subjects with HER2-positive advanced malignant solid tumors, and to initially observe the clinical efficacy of RC48-ADC in the treatment of HER2-positive advanced malignant solid tumors.  **Exploratory objectives:** To analyze the drug-to-antibody ratio (DAR) distribution of RC48-ADC in patients with HER2-positive advanced malignant solid tumors. |
| **Inclusion Criteria** | Enrolled subjects must meet the following criteria:  1. Have signed informed consent forms voluntarily;  2. 18-75 years old;  3. Having an ECOG performance status score of 0 or 1;  4. With an expected survival of more than 12 weeks;  5. Diagnosed histologically or cytologically with local advanced or metastatic HER2-positive malignant solid cancer, and under one of following situations: standard treatment-refractory (disease progression or no response), treatment-resistant, unable to receive treatment, or the standard treatment is unavailable;  6. "HER2-positive" refers to IHC 2+ or 3+ (subjects should go through an FISH assay in extended trial phase, and obtain an HER2 expression of IHC 2+/FISH-, IHC 2+/FISH+, or IHC 3+);  7. Having measurable or evaluable lesions according to RECIST 1.1;  8. Having sufficient bone marrow, liver and kidney functions (based on the normal value of the clinical trial site):  Absolute neutrophil count (ANC) ≥ 1.5×10^9^/L,  Platelets ≥ 100×10^9^/L,  Total serum bilirubin ≤ 1.5×upper limit of normal (ULN),  Without liver metastases, ALT, AST or ALP ≤ 2.5×ULN; with liver metastases, ALT, AST or ALP ≤ 5×ULN,  Normal serum creatinine levels,  International Normalized Ratio (INR) ≤ 1.5×ULN, APTT ≤ 1.5×ULN;  9. Male or female patients of childbearing potential must agree to use effective methods of contraception (such as double-barrier contraceptive methods, condoms, oral or injectable contraceptives and intrauterine devices) during the study period and within 30 days after the last dosing;  10. Echocardiographic LVEF (left ventricular ejection fraction) ≥ 50%. |
| **Exclusion Criteria** | If any one of the following conditions is met, the patients must be excluded from this study:  1. Pregnant (positive pregnancy test prior to dosing) or breast-feeding women;  2. Patients with active Hepatitis B and/or Hepatitis C;  3. Patients who underwent major surgeries within 4 weeks prior to dosing and not fully recovered;  4. Patients who underwent palliative radiotherapy for bone metastasis within 2 weeks before the initiation of the trial;  5. The toxicity of previous anti-cancer therapy has not returned to 0 or 1 level as specified in CTCAE v4.0 (except for hair loss);  6. Patients who received anti-cancer treatment with any other clinical trial drug within 28 days before the first dosing;  7. Patients with clinically significant active infection based on the investigator's judgment;  8. Patients with a history of immunodeficiency, including those are HIV-positive, or patients with other acquired or congenital immune deficiency, or a history of organ transplantation;  9. Patients with severe complications such as active gastrointestinal bleeding, intestinal obstruction, intestinal paralysis, interstitial pneumonia, pulmonary fibrosis, renal failure, glaucoma and uncontrolled diabetes;  10. Patients with a history of acute myocardial infarction, unstable angina pectoris, stroke or transient ischemic attack within 6 months prior to the enrollment, or with NYHA Class 2 or higher congestive heart failure;  11. Patient who has been determined to have insufficient adherence to this clinical study;  12. Patients who has undergone chemotherapy or HER2-targeted therapy within 21 days before the first administration;  13. Patients who underwent long-term treatment with systemic steroids (note: short-term users may be enrolled 2 weeks after drug withdrawal);  14. Patients with uncontrolled primary or metastatic brain tumors;  15. Patients with Grade 2 or higher peripheral neuropathies;  16. Patients with a history of uncontrollable mental illness. |
| **Withdrawal Criteria** | Subjects can withdraw from the study at any time without any reason. Subjects must withdraw from the study when the any of the following conditions occur:  Investigators consider it necessary to stop the trial from the perspective of medical ethics;  Subjects are not suitable to continue with the trial due to severe adverse event(s);  Subjects are poorly compliant and no longer receiving medications or testing before completing all the trials, or receive other anti-cancer therapies before completion of the trials and fail to adhere to the planned completion of trials;  Subjects ask for withdrawal from the trial and withdraw their informed consent forms. |
| **Termination Criteria** | Termination of a trial means that all trials have been discontinued prior to the scheduled completion specified in the protocol. Termination of the trial is mainly to protect the rights of subjects, to ensure the quality of the trial and to avoid unnecessary economic losses. Early termination of clinical trials should be promptly notified to all parties involved in the study.  Major errors are found during the trial so that it is difficult to evaluate the safety of the drug;  The sponsor requires to terminate the trial (e.g, for reasons of funding and management, etc.);  The CFDA or the Ethics Committee order the termination of the trial for some reason. |
| **Dose-Limiting Toxicity (DLT)** | **DLT** refers to the following toxic reactions observed in the DLT evaluation window (observation period of Day 1-21 after the first dose) that are considered related to RC48-ADC treatment by the investigators or sponsor, wherein the grading is based on the 5-level system of the CTCAE v4.0:   - Grade 4 neutropenia. - Neutropenic fever (defined as absolute neutrophil count [ANC] < 1000/mm^3^ accompanied by a fever higher than 38.3°C or a fever above 38°C that persists for more than 1 h). - Grade 3 neutropenia with confirmed infections. - Grade 3 thrombocytopenia with bleeding tendency. - Grade 4 thrombocytopenia. - Non-hematologic toxicity of Grade 3 or higher after supportive care. Except for nausea, vomiting and hair loss. - Renal toxicity ≥ Grade 3. - Neurotoxicity ≥ Grade 2, with no response before the next medication. - Cardiac toxicity ≥ Grade 2. - Hepatic transaminases reach grade 2 at baseline, with a level ≥10×ULN.   If DLT appears in the subjects, treatment of RC48-ADC should be discontinued first, and adverse reactions should be dealt with actively until the toxicity returns to ≤ Grade 1. Thereafter, medication should proceed at a decreased dose level (specific doses will be determined by the investigators).  **On July 4, 2017, the investigators and sponsor decided to modify the DLT definition based on the current study results:**  DLT refers to the following toxic reactions observed in the DLT evaluation window (observation period of Day 1-21 after the first dose) that are considered related to RC48-ADC treatment by the investigators or sponsor, wherein the grading is based on the 5-level system of the CTCAE v4.0:  • Grade 4 neutropenia lasting more than 3 days after symptomatic treatment; or grade 4 neutropenia recurring after recovery to a normal level through symptomatic treatment.  • Neutropenic fever (defined as absolute neutrophil count [ANC] < 1000/mm^3^ accompanied by a fever higher than 38.3°C or a fever above 38°C that persists for more than 1 h).  • Grade 3 neutropenia with confirmed infections.  • Grade 3 thrombocytopenia with bleeding tendency.  • Grade 4 thrombocytopenia.  • Non-hematologic toxicity of Grade 3 or higher after supportive care. Except for nausea, vomiting and hair loss.  • Renal toxicity ≥ Grade 3.  • Neurotoxicity ≥ Grade 2, with no response before the next medication.  • Cardiac toxicity ≥ Grade 2.  • Hepatic transaminases reach grade 2 at baseline, with a level ≥10×ULN.  If DLT appears in the subjects, treatment of RC48-ADC should be discontinued first, and adverse reactions should be dealt with actively until the toxicity returns to ≤ Grade 1. Thereafter, medication should proceed at a decreased dose level (specific doses will be determined by the investigators). |
| **Maximum Tolerated Dose (MTD)** | The maximum tolerated dose (MTD) refers to the lower dosage next to a dose with which DLT occurs in 2 or more out of 6 patients during the DLT evaluation window. |
| **Dose-Escalation Phase** | The doses are proposed to be escalated to 0.1 mg/kg, 0.5 mg/kg, 1.0 mg/kg, 1.5 mg/kg, 2.0 mg/kg, 2.5 mg/kg, 3.0 mg/kg, 3.5 mg/kg and 4.0 mg/kg (if the last dose has not yet reached the MTD, it should continue to increase by 0.5 mg/kg).  On Oct. 18, 2016, the investigators and sponsor decided to add a dose-escalation trial with Q3W administration in the dose-escalation phase. Starting from the 2.0 mg/kg dose group, 2-3 patients are enrolled. Pharmacokinetic data from Q3W administration are collected repeatedly. The dose next to 2.0 mg/kg is determined based on the results of the dose escalation study with Q2W administration. The extended trial is started with the 2.0 mg/kg dose group.  On July 4, 2017, the investigators and sponsor decided that: 1) Symptomatic treatment is allowed during the DLT evaluation period. Dose escalation trials are performed in subjects according to the traditional 3+3 mode, wherein doses are proposed to be escalated from 2.5 mg/kg to 3.0 mg/kg, 3.5 mg/kg and 4.0 mg/kg with Q2W administration until MTD is reached, or the sponsor and investigators decide to terminate the escalation study. 2) The original dose-escalation study starting with 2.0 mg/kg Q3W is stopped. Dose escalation trial is carried out with an initial dose of 1.25mg/kg, followed by 1.5mg/kg, in the traditional 3+3 mode with QW administration. Symptomatic supportive treatment is allowed in the DLT evaluation period until MTD is reached. If the 1.5 mg/kg dose group does not reach MTD, the sponsor and investigators will jointly decide on the next escalating doses or whether to terminate the dose escalation trial. |
| **Extended Trial Phase** | The investigators and sponsor decided to start the extended trial from the 2.0 mg/kg dose group at a meeting on Oct. 18, 2016. Pharmacokinetic data from Q2W administration are collected repeatedly. The dose next to 2.0 mg/kg is determined by the investigators. Q2W dosing means 1 treatment cycle includes 2 doses. The efficacy evaluation is performed once every 2 treatment cycles.  On July 4, 2017, the investigators and sponsor decided to let 3-5 study sites participate in the extended trial phase based on the current study results. At least 10 patients with HER2 ICH 2+/FISH- and at least 20 patients with HER ICH 2+/FISH+ or ICH 3+ with advanced solid tumors (gastric cancer is preferable) are enrolled and administered at 2.0 mg/kg Q2W until disease progression or intolerable adverse reactions. |
| **Safety Evaluation** | Any adverse events occurring during the clinical trial in all subjects, including abnormal clinical symptoms and vital signs and abnormalities in laboratory tests, should be carefully observed and recorded for clinical performance characteristics, severity, time of occurrence, duration, treatment methods and prognosis, and determined for their relevance to the investigational drug. The safety of the drug is evaluated according to NCI CTCAE v4.0. |
| **Efficacy Evaluation** | The RECIST 1.1 standard is used to evaluate the efficacy as CR, PR, SD or PD. |
| **End of Study** | The study ends when the last subject has been dosed for 2 consecutive cycles or in case the disease progresses or the toxicity becomes intolerable, whichever occurs first. Patients entered the extended trial phase continue with the study in accordance with the protocol. |
| **PK Study** | According to the results of non-clinical pharmacokinetics and clinical trial literature of TDM-1^[4-6]^, the following times are tentatively set for blood collection. The first dose group uses the following blood collection points. Specific times of collection may be adjusted, added or subtracted based on the characteristics of RC48-ADC during the actual trial. The tentative blood collection times are as follows.  **Blood Collection for PK During Dose-Escalation Trial:**  Blood collection time points for Q2W administration:  The blood collection points for the first dose (11 blood collection points): before administration (0 h), 0.25 h, at the end of infusion, 1.5 h, 12 h, 24 h, 48 h, 72 h, 120 h, 168 h and 240 h.  For the second dose (1 blood collection point): before administration (336 h).  For the third dose (1 blood collection point): before administration (672 h).  For the fourth dose (12 blood collection points): before administration (1008 h), 0.25 h (1008.25 h), at the end of infusion, 1.5 h (1009.5 h), 12 h (1020 h), 24 h (1032 h), 48 h (1056 h), 72 h (1080 h), 120 h (1128 h), 168 h (1176 h), 240 h (1248 h) and 336 h (1344 h). |
|  | Blood samples are collected at these 25 time points to detect serum ADC, TAb and free MMAE, with a collection volume of 2 mL each time.  Blood collection points for immunogenicity (ADA) assays: Serum anti-ADC antibodies are detected before the first, second, third and fourth doses, and 336 hours after the fourth dose (**5 collection points**), with 1 mL of blood volume for each sample. |
|  | Blood collection time points for Q3W administration:  The blood collection points for the first dose (11 blood collection points): before administration (0 h), 0.25 h, at the end of infusion, 1.5 h, 12 h, 24 h, 48 h, 72 h, 120 h, 168 h and 240 h.  For the second dose (1 blood collection point): before administration (504 h).  For the third dose (12 blood collection points): before administration (1008 h), 0.25 h (1008.25 h), at the end of infusion, 1.5 h (1009.5 h), 12 h (1020 h), 24 h (1032 h), 48 h (1056 h), 72 h (1080 h), 120 h (1128 h), 168 h (1176 h), 336 h (1344 h) and 504 h (1512 h).  Blood samples are collected at these 24 time points to detect serum ADC, TAb and free MMAE, with a collection volume of 2 mL each time.  Blood collection points for immunogenicity (ADA) assays: Serum anti-ADC antibodies were detected before the first, second and third doses, and 504 h after the third dose (4 collection points), with 1 mL of blood volume for each sample.  Blood collection time points for QW administration:  The blood collection points for the first dose (9 blood collection points): before administration (0 h), 0.25 h, at the end of infusion, 1.5 h, 12 h, 24 h, 48 h, 72 h and 120 h.  For the second dose (1 blood collection point): before administration (168 h).  For the third dose (1 blood collection point): before administration (336 h).  For the fourth dose (10 blood collection points): before administration (504 h), 0.25 h (504.25 h), at the end of infusion, 1.5 h (505.5 h), 12 h (516 h), 24 h (528 h), 48 h (552 h), 72 h (576 h), 120 h (624 h) and 168 h (672 h).  Blood collection points for immunogenicity (ADA) assays: Serum anti-ADC antibodies are detected before the first, second, third and fourth doses, and 336 hours after the fourth dose (**5 collection points**), with 1 mL of blood volume for each sample. |
|  | **Blood Collection for PK During Extended Trial:**  Blood collection time points for Q2W administration:  The blood collection points for the first dose (11 blood collection points): before administration (0 h), 0.25 h, at the end of infusion, 1.5 h, 12 h, 24 h, 48 h, 72 h, 120 h, 168 h and 240 h.  For the second dose (1 blood collection point): before administration (336 h).  For the third dose (1 blood collection point): before administration (672 h).  For the fourth dose (12 blood collection points): before administration (1008 h), 0.25 h (1008.25 h), at the end of infusion, 1.5 h (1009.5 h), 12 h (1020 h), 24 h (1032 h), 48 h (1056 h), 72 h (1080 h), 120 h (1128 h), 168 h (1176 h), 240 h (1248 h) and 336 h (1344 h).  Blood samples are collected at these 25 time points to detect serum ADC, TAb and free MMAE, with a collection volume of 2 mL each time.  Blood collection points for immunogenicity (ADA) assays: Serum anti-ADC antibodies are detected before the first, second, third and fourth doses, and 336 hours after the fourth dose, with 1 mL of blood volume for each sample.  The above blood samples will also be used for exploratory purposes. |
| **Statistical Methods** | After the first efficacy evaluation, 3 mL of blood is collected before each administration to detect serum ADC, TAb, free MMAE and serum anti-ADC antibodies.  **Unexpected Blood Collection for PK:** A blood sample of 2 mL is taken for PK analysis immediately no later than 72 hours after SAEs, DLT or dosing suspension.  Pharmacokinetics:  Pharmacokinetic parameters will be calculated using the log-linear trapezoidal rule based on non-compartmental model in Phoenix WinNonlin 6.3 (Pharsight Corp., Mountain View, CA, USA). Descriptive statistics (arithmetic mean, standard deviation, coefficient of variation, minimum, median, and maximum values) for pharmacokinetic parameters will be given in a list according to the trial drugs.  Safety:  The incidence of all adverse events (AEs) should be tabulated by treatment groups. Record the absolute values of the vital signs and mark the out-of-bounds values outside the normal range. The adverse events will be statistically described using the investigators' and MedDRA's terminology records with respect to the list of changes in baseline values. All statistical analyses will be performed using SAS® 9.3. |

**1 Background**

Recombinant Humanized Anti-HER2 Monoclonal Antibody-MMAE Conjugate for Injection (RC48-ADC) is a type of innovative new drug of biological product developed by RemeGen, Ltd. for the treatment of patients with HER2 overexpressing solid malignancies. This clinical trial was conducted after review and approval by China Food and Drug Administration (Acceptance Number: CXSL1400087Lu, Clinical Approval Number: 2015L02291).

**1.1 Pharmacological Efficacy**

The recombinant humanized anti-HER2 monoclonal antibody-MMAE conjugate for injection is a new type of HER2 antibody-drug conjugate (ADC) which is a recombinant humanized anti-HER2 monoclonal antibody through a linker to coupled with the microtubule inhibitor monomethyl auristatin E (MMAE) through a linker, including the HER2 antibody part (also known as RC48 nude antibody), the linker and the cytotoxic pentapeptide MMAE part. The antibody is humanized anti-HER2 IgG1, while the cytotoxic pentapeptide small-molecule MMAE is a kind of microtubule inhibitor. The antibody part binds with high affinity to the HER2 ECD on the cell surface, then the ADC complex is endocytosed and transported to lysosomes. In the acidic lysosomal environment (low pH), the linker of this product is specifically digested by an activated lysosomal enzyme (Cathepsins) and releases cytotoxic pentapeptide small molecules, i.e., tubulin-depolymerizing agent MMAE, which is covalently bonded to the linker. MMAE molecules released in cells bind to microtubules or tubulins to disrupt the intracellular microtubule network, leading to mitosis termination and cell apoptosis. RC48-ADC exerts antitumor effects by 2 major pathways: one is to interfere with cell transcription, growth and proliferation by inhibiting the HER2-activated downstream signaling pathway (e.g., PI3K/AKT); the other is to disturb microtubule formation by small-molecule MMAE, mainly manifested as microtubule depolymerization, so as to induce cell cycle arrest at G2/M phase. In addition, in vitro studies have demonstrated that RC48-ADC has ADCC (antibody-dependent cell-mediated cytotoxicity) effects on cancer cells with HER2 overexpression to suppress tumor growth, as shown in Figure 1.


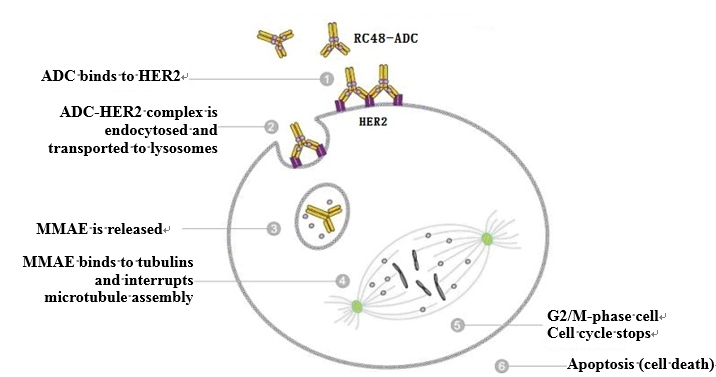


**Figure 1. Action Mechanism of RC48-ADC.**

**1.2 Pharmacodynamic Results**

Both in vitro and in vivo studies have demonstrated that RC48-ADC has significant antitumor activity and is effective against HER2 gene amplification or overexpression in breast, gastric, and ovarian cancers.

The pharmacodynamic studies of RC48-ADC include three parts: the first is the in vitro study of anti-tumor activity and mechanism of RC48-ADC; the second part is the PD/PK study of RC48-ADC on transplanted tumor of breast cancer BT-474/T721 in nude mice resistant to Herceptin; the third part is the in vivo study of anti-tumor effect of RC48-ADC. The mechanism of action and efficacy are summarized according to the results of the three parts of the pharmacodynamic studies.

Pharmacodynamics studies of the three parts showed that the antibody-drug conjugate RC48-ADC could selectively inhibit proliferation of HER2-positive tumor cells, induce cell cycle arrest and apoptosis; had significant therapeutic effect on subcutaneously implanted human tumors with HER2 positive expression in nude mice. Specifically, it was effective for Herceptin- and lapatinib-resistant breast cancers and was more effective than Herceptin and lapatinib. It's important that the above effect of RC48-ADC was greater than the equivalent dose of the reference drug Kadcyla. The in vivo anti-HER2 positive tumor effect of RC48-ADC was significantly greater than that of the same dose of nude antibody combined with equal amount of MMAE. Increase in number of doses could increase its anti-tumor efficacy; RC48-ADC was injected intravenously at 0.5-5mg/kg×Q7D×1-3 doses. A dose-dependent, significant effect was demonstrated in transplanted HER2 positive human tumors in nude mice. This effect was related to the concentration of free MMAE in tumor tissue, and the doses were well tolerated by tumor-bearing mice.These preclinical animal pharmacodynamic studies provide evidence for the pharmacodynamics and potential application aspects of recombinant humanized anti-HER2 monoclonal antibody-MMAE conjugate for injection (code: RC48-ADC) for clinical use in the treatment of HER2-expressing cancers.

**1.3 Animal Pharmacokinetic Studies**

The pharmacokinetics of SD rats and crab-eating macaques after a single intravenous injection or IV infusion of RC48-ADC was assayed by ELISA and LC/MS/MS; ^125^I isotopic labeling assay of RC48-ADC was used to study the tissue distribution and excretion of RC48-ADC in nude mice bearing tumors. The ^125^I isotopic labeling assay of RC48-ADC was also used to perform biliary excretion studies and plasma binding rates in SD rats. The free MMAE was determined by LC-MS/MS and the stability of RC48-ADC incubated in vitro with different species (human, monkey, dog, and rat) at 37°C for 21 days was observed.

**1.3.1 Pharmacokinetics in Rats**

(1) ADC and TAb showed linear pharmacokinetics in rats within the experimental dose range (4, 8 and 16 mg/kg); MMAE showed linearity in the dose range of 4 and 8 mg/kg and non-linear pharmacokinetics when the dose was increased to 16 mg/kg.

(2) After a single intravenous injection of RC48-ADC in rats, serum concentration curves of ADC and TAb of the same animal were gradually separated over time. The levels of TAb and ADC were similar at the early stage of drug administration, but the concentration difference increased gradually over time between the two drugs.

(3) Compared with the same dose of RC48-ADC intravenously injected in rats, the peak concentration of Tab and the exposure level were similar for the combined MMAE and nude antibody group with a consistent half-life, and there was no statistical difference in pharmacokinetic parameters.

(4) The exposure level of MMAEin the ADC group is far lower than that of the combined MMAE and nude antibody at the same dose, which is about one thousandth.

**1.3.2 Pharmacokinetics of Crab-Eating Macaques**

(1) ADC, TAb, and MMAE showed nonlinear pharmacokinetics in crab-eating macaques within the experimental dose range (2, 4 and 8 mg/kg), and the systemic clearance gradually decreased with increasing doses.

(2) After a single intravenous infusion at different doses of RC48-ADC in cynomolgus monkeys, serum concentration and pharmacokinetic parameters of ADC and TAb were similar, with no statistical difference.

(3) The TAb concentration of the nude antibody group was higher than that of the RC48-ADC group

For 4 mg/kg RC48-ADC and 4 mg/kg RC48 nude antibody + 0.08 mg/kg MMAE control group, all pharmacokinetic parameters regarding total antibody except MRT were not statistically different. The plasma concentration of the nude antibody group was higher than that of the RC48-ADC group.

(4) The MMAE exposure level of RC48-ADC was much lower than that of the combined MMAE and nude antibody group at the same dose. The systemic exposure and maximum toxic concentration of RC48-ADC were about 1/5 (18.18%) and 1/310 (0.32%) of those of the latter respectively, suggesting that the free MMAEs detached from RC48-ADC were maintained at a relatively low exposure level in crab-eating macaques' blood.

After a single intravenous injection of combined RC48 nude antibody + MMAE (4+0.08 mg/kg) in the control group, the plasma concentration peaked instantaneously. The peak concentration and plasma exposure of free MMAE after 4 mg/kg RC48-ADC administration were approximately 0.32% and 18.18% of those of the control group at the same dose. This result suggested that the cytotoxic pentapeptide MMAE conjugated to RC48-ADC was stable in the blood, and the systemic exposure of free MMAE within 14 days after a single intravenous infusion in crab-eating macaques was less than 20% of the amount of its conjugate. The maximum toxic concentration was reduced by more than 300 folds, theoretically reducing the systemic toxicity caused by MMAE.

**1.3.3 Tissue Distribution Characteristics of HER2-Positive Tumor-Bearing Mice Presents with Targeting to Tumor Tissues**

Tissue distribution characteristics in HER2-positive tumor-bearing mice have demonstrated the drug's targeting ability to tumor tissues and inability to cross the blood brain barrier. After intravenous injection of 125I-RC48-ADC (12 mg/kg), the exposure level (AUC) of TCA precipitable fraction in tissues was ranked in descending order as follows: serum, tumor, liver, adrenal gland, kidney, lung, urine, submandibular gland, gonads, lymph nodes, spleen, heart, bladder, small intestine, fat, muscle, pancreas, eyeballs, feces, intestinal contents, brain and bone marrow.

**1.3.4 The drug is excreted predominantly via urine with a small amount excreted via feces in HER2-positive tumor-bearing mice**

**Excretion characteristics:** Predominantly excreted via urine, with a small amount excreted via feces and bile. After intravenous injection of ^125^I-RC48-ADC (12 mg/kg) in tumor-bearing mice, slow radioactive excretion rate was observed, with 75.64% ± 2.80% and 0.90% ± 0.13% of injected radioactive dose being excreted in urine and feces at 336 h. The radioactive excretion in urine and feces totaled 76.53% ± 2.80% of the administered radioactive dose. SEC-HPLC assay showed that metabolites in urine were small molecular degradation products without parent drug. Cumulative excretion ratio in urine accounted for 0.17%±0.06% of administered radioactive dose 24 h after intravenous injection of ^125^I-RC48-ADC (8 mg/kg) in rats.

**1.3.5 Inability to bind to plasma proteins in tumor-bearing mice**

The chromatographic analysis of blood samples after administration in tumor-bearing mice revealed that the drug was predominantly present in the form of ^125^I-RC48-ADC, not binding to plasma proteins.

**1.3.6 Stability test of RC48-ADC in plasma in vitro**

Results showed that: (1) RC48-ADC was stable in plasma of various animal species, with less than 1.8% of free MMAE present after 21-day storage at 37°C. That was comparable to SGN-35, whose MMAE dissociation rate was approximately 2% under the same condition. (2) RC48-ADC was stable in the plasma of different animal species with a descending stability order as follows: monkey > dog > human > rat. This result was consistent with the stability of SGN-35 in plasma.

**1.4 Safety Evaluation Study in Animals**

According to regulations and guidelines concerning drug evaluation in China, following studies were carried out on RC48-ADC under GLP conditions: tissue-binding study with normal tissues in humans, cynomolgus monkeys and SD rats; general pharmacological study in cynomolgus monkeys and SD rats; acute toxicity study with single intravenous infusions in cynomolgus monkeys and SD rats; chronic toxicity study with repeated intravenous infusions for 12 weeks followed by a recovery period of 6 weeks in SD rats and cynomolgus monkeys; special toxicity (hemolysis) study; active systemic anaphylaxis test in guinea pigs; and immunotoxicity and/or immunogenicity study, local irritation study and toxicokinetic study in combination with chronic toxicity tests. Major results are as follows:

**Tissue cross-reactivity study** showed that the RC48-ADC and its nude antibody exhibited similar cross reactivity with normal human and cynomolgus monkey tissues. Both of them binded specifically to normal tissues of bladder, skin, mammary gland, pancreas, placenta, kidney, prostate, ureter, fallopian tube and stomach in humans and normal tissues of bladder, mammary gland, pancreas, liver, lung, kidney, prostate and ureter in cynomolgus monkeys. RC48-ADC showed no specific binding to normal tissues in SD rat, while the control RC48 nude antibody bound specifically to normal gastric tissues in SD rats. It can be seen from the tissue cross-reactivity study that cynomolgus monkeys, rather than rats, are the relevant animal for RC48-ADC administration in humans.

**General pharmacological study** demonstrated that RC48-ADC and its constituents (RC48 nude antibody and MMAE) have no significant effects on mental and nervous system of SD rats, and RC48-ADC and MMAE have no significant effects on cardiovascular system and respiration of awake monkeys monitored remotely.

**Acute toxicity study** showed that MTD was measured to be 24 mg/kg, 40 mg/kg and 0.4 mg/kg in SD rats and 10 mg/kg, 27 mg/kg and 0.12 mg/kg in cynomolgus monkeys after single intravenous injections of RC48-ADC, RC48 nude antibody and MMAE, respectively.

**Chronic toxicity study** showed that MTD was measured to be 12 mg/kg, 24 mg/kg (Q2W×4) and 0.2 mg/kg in SD rats and 10 mg/kg, 12 mg/kg and 0.12 mg/kg in cynomolgus monkeys, after repeated intravenous infusions Q2W of RC48-ADC, RC48 nude antibody and MMAE for 7 times in total, respectively. Major toxic reactions included immunosuppression and hematopoietic inhibition in both rats and monkeys, and digestive tract injuries and testicular and epididymis atrophies were observed in rats. RC48-ADC and MMAE have basically the same toxic reactions.

**Allergy study** showed positive active systemic anaphylaxis for RC48-ADC and RC48 nude antibody, and negative anaphylaxis for the pentapeptide constituent MMAE in guinea pigs.

**Immunotoxicity and/or immunogenicity study** demonstrated the immunosuppression of RC48-ADC. After repeated intravenous infusions, incidence of immunogenicity (ADA) increased with dose in SD rats, and decreased with dose in cynomolgus monkeys. This may be due to the immunosuppression in cynomolgus monkeys.

**Special toxicity study** demonstrated by local irritation tests that no irritative reaction was found at injection sites after repeated Q2W intravenous infusions of RC48-ADC in cynomolgus monkeys at 0.6-2.4 mg/mL and rats at 1.2-4.8 mg/mL. In vitro hemolysis tests showed no hemolysis or agglutination in response to the administration of RC48-ADC at 1.8mg/mL.

Results of safety evaluation tests suggested that cynomolgus monkeys are the relevant animal for simulate administration of RC48-ADC in humans. RC48-ADC and MMAE induced basically the same toxic reactions in cynomolgus monkeys and SD rats, wherein the major toxic reactions included immunosuppression and hematopoietic inhibition. No administration-related irritative reaction was found at injection sites. RC48-ADC group exhibited a less severe toxic reaction than MMAE group with an equal dose of MMAE. No significant toxic reactions were found in RC48 nude antibody group.

**1.5 Preliminary Study Results**

As of July 1, 2017, a total of 25 valid subjects have been enrolled in the study. Dose escalation has reached 2.5 mg/kg, with DLT of Grade 4 neutropenia occurring in 2 cases at the dose of 2.5 mg/kg. Drug-related adverse events (≥5%) included leukopenia (14%), neutropenia (9%), decreased hemoglobin (7%) and numbness (6%). Adverse events that led to changes in medication (including dose reduction, dosing suspension and medication termination) were leukopenia, neutropenia and numbness of hands and feet. All symptoms were resolved after dose discontinuation or symptomatic treatment.

As of July 1, 2017, efficacy evaluation has been conducted on 19 subjects, among which 3 were evaluated as partial response (PR), 8 were stable disease (SD), and 8 were progressive disease (PD). The duration time for administration is up to 238 days.

Pharmacokinetic study showed that half-lives of RC48-ADC in the groups of 1.0 mg/kg, 2.0mg/kg and 2.5mg/kg were 16.2±9.5 h, 34.4±13.7 h and 49.0±27.4 h, respectively. Exposure level AUC_0-∞_ increased with dose. No anti-drug antibody was detected so far.

**2 Study Objectives**

**Primary objectives:** To determine the maximum tolerated dose (MTD) and safety of RC48-ADC in patients with HER2-positive advanced malignant solid tumors, and to determine the recommended dose in the Phase II clinical trial.

**Secondary objectives:** To investigate the pharmacokinetic (PK) characteristics of RC48-ADC in patients with HER2-positive advanced malignant solid tumors, and to initially observe the clinical efficacy of RC48-ADC in the treatment of HER2-positive advanced malignant solid tumors.

**Exploratory objectives:** To analyze the drug-to-antibody ratio (DAR) of RC48-ADC over time in patients with advanced HER2-positive malignant solid tumors.

**3 Study Design**

It is expected to set up 9 dose groups to conduct an open-label, dose-escalation study. Patients with HER2-positive solid tumors are enrolled.

The doses are proposed to be escalated to 0.1 mg/kg, 0.5 mg/kg, 1.0 mg/kg, 1.5 mg/kg, 2.0 mg/kg, 2.5 mg/kg, 3.0 mg/kg, 3.5 mg/kg and 4.0 mg/kg.

If a dose has passed the safety observations in another Phase I clinical trial (at the Cancer Hospital, Chinese Academy of Medical Sciences), investigators and sponsors may meet to discuss and adjust the dose increment in dose escalation trial, or skip this dose and proceed directly to the next dose according to the corresponding results, in order to reduce the patient's exposure to ineffective doses.

During the trial, the dose increment and the maximum dose can be adjusted based on actual results after consultation between the investigators and sponsor.

The first dose (0.1 mg/kg) is given to 1 subject to collect pharmacokinetic data of a single dose. After safety observations, the patient can continue with trials of the following dose levels on a voluntary basis without additional collection of pharmacokinetic data (this scheme is suitable to all subsequent subjects to ensure their benefits).

The second dose (0.5 mg/kg) group enrolls another subject. Pharmacokinetic data of a single dose are collected for this patient after first dosing. Dosing interval (Q1W, Q2W or Q3W) will be determined by the investigators and sponsor based on the data obtained from the 2 patients. Q2W was tentatively selected at a meeting on Jan. 26, 2016. Q2W dosing means 1 treatment cycle includes 2 doses.

Repeated doses are given to subjects from the second dose (0.5 mg/kg) forward to collect pharmacokinetic data of repeated doses. Pharmacokinetic data are collected before and after each of 4 doses for one patient, no matter what dosing interval is used.

Subjects are enrolled in the traditional 3+3 mode from the third dose (1.0 mg/kg) forward. At first, 3 qualified subjects are enrolled into the 1.0 mg/kg group. If no DLT is observed in the 3 subjects during the DLT evaluation period, the dose is escalated to the next level; if DLT occurs in 1/3 of the subjects (1 case) in the 1.0 mg/kg group, another 3 subjects should be added. If no DLT is observed in the additional 3 subjects, trials at the next dose level may be started; if DLT occurs in 1 or more of the 3 additional subjects, or in 2 or more of the 6 subjects in total, the dose escalation should be discontinued. Trials in the same manner are performed with dose escalation until the maximum tolerated dose (MTD) and recommended phase Ⅱ dose (RP2D) are determined.


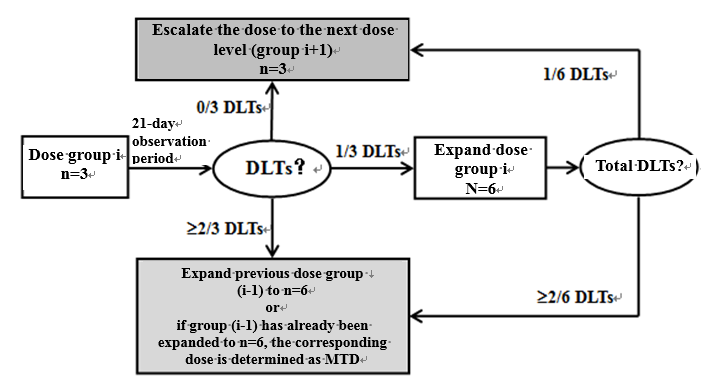


All subjects are evaluated as CR, PR or SD after the 4th administration. If the patients are willing to do so, and the investigator determines that continued administration will be of benefit to the patients, patients can enter the extended test in which treatment is continued until the disease progresses, the toxicity become intolerable, the patients withdraw consent or the investigator determines that the medication must be discontinued.

**Dose-limiting toxicity (DLT)** refers to the following toxic reactions observed in the DLT evaluation window (observation period of Day 1-21 after the first dose) that are considered related to RC48-ADC treatment by the investigators or sponsor, wherein the grading is based on the 5-level system of the CTCAE v4.0:

- Grade 4 neutropenia.
- Neutropenic fever (defined as absolute neutrophil count ^[^ANC] < 1000/mm3 accompanied by a fever higher than 38.3 °C or a fever above 38 °C that persists for more than 1 h).
- Grade 3 neutropenia with confirmed infections.
- Grade 3 thrombocytopenia with bleeding tendency.
- Grade 4 thrombocytopenia.
- Non-hematologic toxicity of Grade 3 or higher after supportive care. Except for nausea, vomiting and hair loss.
- Renal toxicity ≥ Grade 3.
- Neurological toxicities ≥ Level 2 that persist until the next administration.
- Cardiac toxicity ≥ Grade 2.
- Hepatic transaminases reach grade 2 at baseline, with a level ≥10×ULN.

If DLT appears in the subjects, treatment of RC48-ADC should be discontinued first, and adverse reactions should be dealt with actively until the toxicity returns to ≤ Grade 1. Thereafter, medication should proceed at a decreased dose level (specific doses will be determined by the investigators).

**Maximum Tolerated Dose (MTD)**: The lower dosage next to a dose with which DLT occurs in 2 or more out of 6 patients during the DLT evaluation window.

Dose groups of 3 to 6 subjects were formed starting with the lowest dose set by the protocol:

(1) If none of the 3 subjects develop DLT within the 21-day observation period after the first dose, the next (escalated) dose trial can be performed.

(2) If DLT occurs in 1 of the 3 subjects, the number of subjects in this dose group should be increased to 6: (a) if DLT occurs in 2 or more of these 6 subjects, the next (escalated) dose trial should be terminated; (b) if DLT occurrs in no more than 1 of the 6 patients, the next (escalated) dose trial can be performed.

**On July 4, 2017, the investigators and sponsor decided to modify the DLT definition based on the current study results:**

**DLT** refers to the following toxic reactions observed in the DLT evaluation window (observation period of Day 1-21 after the first dose) that are considered related to RC48-ADC treatment by the investigators or sponsor, wherein the grading is based on the 5-level system of the CTCAE v4.0:

- Grade 4 neutropenia lasting more than 3 days after symptomatic treatment; or grade 4 neutropenia recurring after recovery to a normal level through symptomatic treatment.
- Neutropenic fever (defined as absolute neutrophil count ^[^ANC] < 1000/mm3 accompanied by a fever higher than 38.3 °C or a fever above 38 °C that persists for more than 1 h).
- Grade 3 neutropenia with confirmed infections.
- Grade 3 thrombocytopenia with bleeding tendency.
- Grade 4 thrombocytopenia.
- Non-hematologic toxicity of Grade 3 or higher after supportive care. Except for nausea, vomiting and hair loss.
- Renal toxicity ≥ Grade 3.
- Neurological toxicities ≥ Level 2 that persist until the next administration.
- Cardiac toxicity ≥ Grade 2.
- Hepatic transaminases reach grade 2 at baseline, with a level ≥10×ULN.

If DLT appears in the subjects, treatment of RC48-ADC should be discontinued first, and adverse reactions should be dealt with actively until the toxicity returns to ≤ Grade 1. Thereafter, medication should proceed at a decreased dose level (specific doses will be determined by the investigators).

**Maximum Tolerated Dose (MTD)**: The lower dosage next to a dose with which DLT occurs in 2 or more out of 6 patients during the DLT evaluation window.

**Extended Trial**

The investigators and sponsor decided to start the extended trial from the 2.0 mg/kg dose group at a meeting on Oct. 18, 2016. Pharmacokinetic data from Q2W administration are collected repeatedly. The dose next to 2.0 mg/kg is determined by the investigators. Q2W dosing means 1 treatment cycle includes 2 doses. The efficacy evaluation is performed once every 2 treatment cycles.

On July 4, 2017, the investigators and sponsor decided to let 3-5 study sites participate in the extended trial phase based on the current study results. At least 10 patients with HER2 ICH 2+/FISH- and at least 20 patients with HER ICH 2+/FISH+ or ICH 3+ with advanced solid tumors (gastric cancer is preferable) are enrolled and administered at 2.0 mg/kg Q2W until disease progression or intolerable adverse reactions.

# 4 Criteria for Subject Selection

## 4.1 Inclusion Criteria

1. Have signed informed consent forms voluntarily;

2. 18-75 years old;

3. Having an ECOG performance status score of 0 or 1;

4. With an expected survival of more than 12 weeks;

5. Diagnosed histologically or cytologically with local advanced or metastatic HER2-positive malignant solid cancer, and under one of following situations: standard treatment-refractory (disease progression or no response), treatment-resistant, unable to receive treatment, or the standard treatment is unavailable;

6. "HER2-positive" refers to IHC 2+ or 3+ (subjects should go through an FISH assay in extended trial phase, and obtain an HER2 expression of IHC 2+/FISH-, IHC 2+/FISH+, or IHC 3+);

7. Having measurable or evaluable lesions according to RECIST 1.1;

8. Having sufficient bone marrow, liver and kidney functions (based on the normal value of the clinical trial site):

Absolute neutrophil count (ANC) ≥ 1.5×109/L,

Platelets ≥ 100×109/L,

Total serum bilirubin ≤ 1.5×upper limit of normal (ULN),

Without liver metastases, ALT, AST or ALP ≤ 2.5×ULN; with liver metastases, ALT, AST or ALP ≤ 5×ULN, with normal serum creatinine levels and INR ≤ 1.5×ULN, APTT ≤ 1.5×ULN;

9. Male or female patients of childbearing potential must agree to use effective methods of contraception (such as double-barrier contraceptive methods, condoms, oral or injectable contraceptives and intrauterine devices) during the study period and within 30 days after the last dosing;

10. Echocardiographic LVEF (left ventricular ejection fraction) ≥ 50%.

## 4.2 Exclusion Criteria

If any one of the following conditions is met, the patients must be excluded from this study:

1. Pregnant (positive pregnancy test prior to dosing) or breast-feeding women;

2. Patients with active Hepatitis B and/or Hepatitis C;

3. Patients who underwent major surgeries within 4 weeks prior to dosing and not fully recovered;

4. Patients who underwent palliative radiotherapy for bone metastasis within 2 weeks before the initiation of the trial;

5. The toxicity of previous anti-cancer therapy has not returned to 0 or 1 level as specified in CTCAE v4.0 (except for hair loss);

6. Patients who received anti-cancer treatment with any other clinical trial drug within 28 days before the first dosing;

7. Patients with clinically significant active infection based on the investigator's judgment;

8. Patients with a history of immunodeficiency, including those are HIV-positive, or patients with other acquired or congenital immune deficiency, or a history of organ transplantation;

9. Patients with serious complications such as gastrointestinal bleeding, intestinal obstruction, intestinal paralysis, interstitial pneumonia, pulmonary fibrosis, renal failure, glaucoma and uncontrolled diabetes;

10. Patients with a history of acute myocardial infarction, unstable angina pectoris, stroke or transient ischemic attack within 6 months prior to the enrollment, or with NYHA Class 2 or higher congestive heart failure;

11. Patient who has been determined to have insufficient adherence to this clinical study;

12. Patients who has undergone chemotherapy or HER2 targeted therapy within 21 days before the first administration;

13. Patients who underwent long-term treatment with systemic steroids (note: short-term users may be enrolled 2 weeks after drug withdrawal);

14. Patients with uncontrolled primary or metastatic brain tumors;

15. Patients with Grade 2 or higher peripheral neuropathies;

16. Patients with a history of uncontrollable mental illness.

## 4.3 Withdrawal Criteria

Subjects can withdraw from the study at any time without any reason. Subjects must withdraw from the study when the any of the following conditions occur:

Investigators consider it necessary to stop the trial from the perspective of medical ethics;

Subjects are not suitable to continue with the trial due to severe adverse event(s);

Subjects are poorly compliant and no longer receiving medications or testing before completing all the trials, or receive other anti-cancer therapies before completion of the trials and fail to adhere to the planned completion of trials;

Subjects ask for withdrawal from the trial and withdraw their informed consent forms.

## 4.4 Criteria for Termination of Trial

Termination of a trial means that all trials have been discontinued prior to the scheduled completion specified in the protocol. Termination of the trial is mainly to protect the rights of subjects, to ensure the quality of the trial and to avoid unnecessary economic losses. Early termination of clinical trials should be promptly notified to all parties involved in the study.

Major errors are found during the trial so that it is difficult to evaluate the safety of the drug;

The sponsor requires to terminate the trial (e.g, for reasons of funding and management, etc.);

The CFDA or the Ethics Committee order the termination of the trial for some reason.

# 5. Investigational Drug

(1) Test Product: omitted

(2) Intended Route and Method of Clinical Administration: Intravenous infusion, not to be given by intravenous push or bolus. Measure 60 mg of this product and dissolve with 6.0 mL of sterile water for injection. After dissolution, dilute with 5% glucose injection to about 250 mL for intravenous infusion. The diluted RC48-ADC infusion solution should be used immediately. Otherwise, the solution can be stored in a refrigerator at 2 °C to 8 °C for 24 h. Do not shake.

Administer by intravenous drip for at least 30 min.

Patient should be observed during the first infusion and at least 90 minutes after start of the administration for fever or chills.

Subsequent infusion: If the previous infusions are well tolerated, the patient should be observed during the infusion and at least 30 minutes after the infusion.

Physical signs and symptoms should be monitored during and after infusion. If serious infusion-related reactions or hypersensitivity reactions occur, slow down or suspend the infusion and give appropriate medical treatment. Terminate permenantly in the event of life-threatening reactions to infusion.

For detailed drug formulation, refer to the "Operation Manual of RC48-ADC Investigational Drug Formulation".

RC48-ADC local irritation tests showed that in cynomolgus monkeys and SD rats each given repeated intravenous infusions Q2W at respective concentrations of 0.6-2.4 mg/mL and 1.2-4.8 mg/mL, no irritation was seen in response to drug administration. In vitro hemolysis testing found that RC48-ADC at 1.8 mg/mL did not exhibit hemolytic or agglutinating effects. It can be concluded that a concentration of infused RC48-ADC for human should be safely tolerated within 1.8 mg/mL.

(3) Specification: 60 mg/piece;

(4) Shelf life: 18 months before retest;

(5) Storage condition: 2-8 °C refrigerated storage and transportation;

(6) Administration: Intravenous infusion.

The initial dose of this study is determined 0.1 mg/kg and the maximum dose is 4.0 mg/kg. A total of 9 groups are required. According to animal experimental results, the possible MTD of this product is predicted to be between 2-3.5 mg/kg. The number of cases in each group is shown in Table 1.

**Table 1. Dosage escalation grouping.**

| **Dose** | **Number of cases** |
| --- | --- |
| 0.1 mg/kg | 1 |
| 0.5 mg/kg | 1 |
| 1.0 mg/kg | 3-6 |
| 1.5 mg/kg | 3-6 |
| 2.0 mg/kg | 3-6 |
| 2.5 mg/kg | 3-6 |
| 3.0 mg/kg | 3-6 |
| 3.5 mg/kg | 3-6 |
| 4.0 mg/kg | 3-6 |

Subjects are enrolled in the traditional 3+3 mode from 1.0 mg/kg dose group forward.

If a dose has passed the safety observations in another Phase I clinical trial (at the Cancer Hospital, Chinese Academy of Medical Sciences), investigators and sponsors may adjust the dose increment in dose escalation trial, or skip this dose and proceed directly to the next dose according to the corresponding results, in order to reduce the patient's exposure to ineffective doses.

During the trial, investigators can adjust the dose increment and maximum dosage based on the results of the trial.

On Oct. 18, 2016, investigators and sponsors met and determined an additional dose escalation study for Q3W administration. Starting from the 2.0 mg/kg dose group, 2-3 patients are enrolled. Pharmacokinetic data from Q3W administration are collected repeatedly. The dose next to 2.0 mg/kg is determined based on the results of the dose escalation study with Q2W administration.

2) The original dose-escalation study starting with 2.0 mg/kg Q3W was discontinued. Instead, dose escalation trial is carried out with an initial dose of 1.25 mg/kg, followed by 1.5 mg/kg, in the traditional 3+3 mode with QW administration. Symptomatic supportive treatment is allowed in the DLT evaluation period until MTD is reached. If the 1.5 mg/kg dose group does not reach MTD, the sponsor and investigators will jointly decide on the next escalating doses or whether to terminate the dose escalation trial.

**Extended Trial**

The investigators and sponsor decided to start the extended trial from the 2.0 mg/kg dose group at a meeting on Oct. 18, 2016. Pharmacokinetic data from Q2W administration are collected repeatedly. The dose next to 2.0 mg/kg is determined by the investigators. Q2W dosing means 1 treatment cycle includes 2 doses. The efficacy evaluation is performed once every 2 treatment cycles.

On July 4, 2017, the investigators and sponsor decided to let 3-5 study sites participate in the extended trial phase based on the current study results. At least 10 patients with HER2 ICH 2+/FISH- and at least 20 patients with HER ICH 2+/FISH+ or ICH 3+ with advanced solid tumors (gastric cancer is preferable) are enrolled and administered at 2.0 mg/kg Q2W until disease progression or intolerable adverse reactions.

**Basis for Dosage Interval**

According to the findings of in vitro anti-tumor tests with RC48-ADC, RC48-ADC can inhibit the proliferation of cells with high-expression of HER2. After incubation with breast cancer BT-474 cells (highly expressing HER2) for 1 hour, RC48-ADC (1 μg/mL) and RC48 nude antibody (1 μg/mL) were predominantly localized on the cell membrane. After incubation for 24 hours, they had for the most part entered the cells and were mostly consistent with lysosomes in locations. RC48-ADC and RC48 nude antibodies are known to have similar action characteristics. Therefore, RC48-ADC was predicted to be effective at least 24 h after entering the body.

Mean T1/2 of rat serum ADC after a single intravenous administration of RC48-ADC (4, 8, 16 mg/kg) and SGN-35 (0.5, 5 mg/kg) were 4.9-7.5 days (2.7-62.7 d) and 8.5-14.6 d respectively. There was no dosage-related difference in the half-life and clearance rate within the experimental range, and the exposure was found to increase with the dosage. As different study and measurement methods were applied, it was established that there was no difference in the overview.

The animal trials also showed that RC48-ADC was able to significantly inhibit the phosphorylation of AKT in BT-474/T721 tumor tissues. 4~24 h after RC48-ADC administration, all dosages (1.5 mg/kg~5 mg/kg) showed significant inhibitory effects on AKT phosphorylation and lasted up to 168 h (7 days).

In animal trials, RC48-ADC (1.5 mg/kg and 5 mg/kg) was administered by a single intravenous injection to tumor-bearing nude mice. PK studies found that the level of free MMAE in the serum decreased rapidly, while tumor tissues significantly grew and reached a relatively high level at 24 h. Within the range of 1.5-5 mg/kg, free MMAE in tumor tissues peaked within 1-3 days and remained at a relatively high plateau level within 7 days (refer to Figure 2).

Figure 2. Mean concentration-time curves of free MMAE in serum and tumor tissue after a single IV RC48-ADC injection at 1.5 mg/kg and 5.0 mg/kg to Herceptin-resistant breast cancer BT-474/T721 tumor-bearing nude mice (n=3).

The drug concentration in tumor tissues is in ng/g of tumor weight; and the drug concentration in serum is in ng/mL of serum.

**Basis of Dosage Design**

The initial and maximum doses for this trial were determined based on both data from preclinical animal trials and data from clinical trials of TDM-1 and SGN-35 on the market, combined with the Phase I clinical trial conducted simultaneously.

The following refers to the relevant experimental data of the listed TDM-1 and SGN-35. Refer to Table 2 for details.

**Table 2. Comparison of general information on 3 types of ADC drugs.**

| **ADC Study Code** | **RC48-ADC** | **TDM-1** | **SGN-35** |
| --- | --- | --- | --- |
| **Common Name** | Recombinant Humanized Anti-HER2 Monoclonal Antibody-MMAE Conjugate for Injection | Ado-trastuzumab emtansine | Brentuximab vedotin |
| **Brand Name** | None | KADCYLA | ADCETRIS |
| **Antibody** | HER2 | HER2 | CD30 |
| **Molecule** | MMAE | DM1 | MMAE |
| **Antibody Target** | HER2 | HER2 | CD30 |
| **Tumor-bearing Mice**  **Effective Dose** | 1.5 mg/kg | 5 mg/kg | 1 mg/kg |

MTDs of RC48-ADC (Q2W x 7), SGN-35 (Q3W x 4) and TDM-1 (Q2W x 4) were 10, 3 and 10 mg/kg respectively. MTDs of RC48-ADC and TDM-1 were similar, while SMD-35 had the lowest MTD.

Effective doses of RC48-ADC and TDM-1 in Herceptin-resistant animal models were 1.5 mg/kg and 5 mg/kg respectively, and those in herceptin- and lapatinib-resistant animal models were 5 mg/kg and 15 mg/kg respectively. Hence, the estimated effective dose of RC48-ADC is approximately one-third of TDM-1. The effective dose of SGN-35 in animal in vivo efficacy study was 1 mg/kg, slightly lower than that of RC48-ADC, hence the effective doses of these two drugs are equivalent. Among these three ADC drugs, SGN-35 has the lowest effective dose and MTD, while TDM-1 has the highest effective dose and MTD. The effective dose of RC48-ADC is similar to that of SGN-35, and its safe dose is similar to TDM-1. Therefore, the therapeutic window of RC48-ADC is the largest among the three drugs. The recommended dose of listed SGN-35 is 1.8 mg/kg. Based on the comparison of animal experimental data, the effective dose of RC48-ADC is similar to that of SGN-35, hence the effective dose of RC48-ADC is expected to be greater than 1.8 mg/kg.

The molecular weight of RC48-ADC is 149 kD. Therefore, RC48-ADC is considered to be an intravascularly administered protein with a molecular weight of more than 100 kD and should be measured in mg/kg.

According to the "Guiding Principles for Clinical Trial Techniques of Anti-Tumor Drugs" and "Technical Guidelines for Non-Clinical Studies of Cytotoxic Anti-Cancer Drugs" issued by China, the initial dose of Phase I clinical trials was estimated as follows: the MTD obtained from rat multiple-dose toxicological study was 12 mg/kg, with the equivalent dose for human was 1.92 mg/kg and the safety factor was 10. The calculation of 1/10 gave the initial dose of 0.192 mg/kg. As a related animal, crab-eating macaque was given multiple doses (Q2W x 7) during toxicological study, providing an MTD of 10 mg/kg. The human equivalent dose was 3.2 mg/kg and the calculation of 1/6 gave the initial dose of 0.53 mg/kg.

According to the FDA's "Estimating the Maximum Safe Starting Dose in Initial Clinical Trials for Therapeutics in Adult Healthy Volunteers", the starting dose was calculated to be 0.096 mg/kg, which was approximately equal to 0.1 mg/kg.

**Table 3. Comparison of calculation results from different animal data.**

| species | NOAEL | kmanimal/kmhuman | HED | MRSD |
| --- | --- | --- | --- | --- |
| Rat | 6 mg/kg | ×0.16 | 0.96 mg/kg | 0.096 mg/kg |
| Crab-eating Macaque | 3 mg/kg | ×0.32 | 0.96 mg/kg | 0.096 mg/kg |

Based on the above data, investigators decided to select 0.1 mg/kg as the initial dose and 1 patient to collect a single set of pharmacokinetics. In order to reduce the patient's exposure to an ineffective dosage, the patient do not undergo further administration of this dosage. The 2nd dose is 0.5 mg/kg and 1 patient is selected for the collection of a single set of pharmacokinetics. There is an interval of 21 days after the first administration. Based on the results of the single sets of pharmacokinetics from these 2 patients within 21 days, investigators should decide whether the interval of administration in this trial is Q1W, Q2W or Q3W. Q2W was tentatively selected at a meeting on Jan. 26, 2016. Each treatment cycle includes 2 doses.

Repeated doses are given to subjects from the second dose (0.5 mg/kg) forward to collect pharmacokinetic data of repeated doses. Pharmacokinetic data are collected before and after each of 4 doses for one patient, no matter what dosing interval is used.

# 6. Blood Collection Times and Outcome Measures

According to the results of non-clinical pharmacokinetics and clinical trial literature of TDM-1[4-6], the following times are tentatively set for blood collection. The first dose group uses the following blood collection points. Specific times of collection may be adjusted, added or subtracted based on the characteristics of RC48-ADC during the actual trial. The tentative blood collection times are as follows.

**Blood Collection for PK During Dose-Escalation Trial:**

Blood collection time points for Q2W administration:

The blood collection points for the first dose (11 blood collection points): before administration (0 h), 0.25 h, at the end of infusion, 1.5 h, 12 h, 24 h, 48 h, 72 h, 120 h, 168 h and 240 h.

For the second dose (1 blood collection point): before administration (336 h).

For the third dose (1 blood collection point): before administration (672 h).

For the fourth dose (12 blood collection points): before administration (1008 h), 0.25 h (1008.25 h), at the end of infusion, 1.5 h (1009.5 h), 12 h (1020 h), 24 h (1032 h), 48 h (1056 h), 72 h (1080 h), 120 h (1128 h), 168 h (1176 h), 240 h (1248 h) and 336 h (1344 h).

Blood samples are collected at these 25 time points to detect serum ADC, TAb and free MMAE, with a collection volume of 2 mL each time.

Blood collection points for immunogenicity (ADA) assays: Serum anti-ADC antibodies are detected before the first, second, third and fourth doses, and 336 hours after the fourth dose (5 collection points), with 1 mL of blood volume for each sample.

Blood collection time points for Q3W administration:

The blood collection points for the first dose (11 blood collection points): before administration (0 h), 0.25 h, at the end of infusion, 1.5 h, 12 h, 24 h, 48 h, 72 h, 120 h, 168 h and 240 h.

For the second dose (1 blood collection point): before administration (504 h).

For the third dose (12 blood collection points): before administration (1008 h), 0.25 h (1008.25 h), at the end of infusion, 1.5 h (1009.5 h), 12 h (1020 h), 24 h (1032 h), 48 h (1056 h), 72 h (1080 h), 120 h (1128 h), 168 h (1176 h), 336 h (1344 h) and 504 h (1512 h).

Blood samples are collected at these 24 time points to detect serum ADC, TAb and free MMAE, with a collection volume of 2 mL each time.

Blood collection points for immunogenicity (ADA) assays: Serum anti-ADC antibodies were detected before the first, second and third doses, and 504 h after the third dose (4 collection points), with 1 mL of blood volume for each sample.

Blood collection time points for QW administration:

The blood collection points for the first dose (9 blood collection points): before administration (0 h), 0.25 h, at the end of infusion, 1.5 h, 12 h, 24 h, 48 h, 72 h and 120 h.

For the second dose (1 blood collection point): before administration (168 h).

For the third dose (1 blood collection point): before administration (336 h).

For the fourth dose (10 blood collection points): before administration (504 h), 0.25 h (504.25 h), at the end of infusion, 1.5 h (505.5 h), 12 h (516 h), 24 h (528 h), 48 h (552 h), 72 h (576 h), 120 h (624 h) and 168 h (672 h).

Blood collection points for immunogenicity (ADA) assays: Serum anti-ADC antibodies are detected before the first, second, third and fourth doses, and 336 hours after the fourth dose (5 collection points), with 1 mL of blood volume for each sample.

**Blood Collection for PK During Extended Trial:**

Blood collection time points for Q2W administration:

The blood collection points for the first dose (11 blood collection points): before administration (0 h), 0.25 h, at the end of infusion, 1.5 h, 12 h, 24 h, 48 h, 72 h, 120 h, 168 h and 240 h.

For the second dose (1 blood collection point): before administration (336 h).

For the third dose (1 blood collection point): before administration (672 h).

For the fourth dose (12 blood collection points): before administration (1008 h), 0.25 h (1008.25 h), at the end of infusion, 1.5 h (1009.5 h), 12 h (1020 h), 24 h (1032 h), 48 h (1056 h), 72 h (1080 h), 120 h (1128 h), 168 h (1176 h), 240 h (1248 h) and 336 h (1344 h).

Blood samples are collected at these 25 time points to detect serum ADC, TAb and free MMAE, with a collection volume of 2 mL each time.

Blood collection points for immunogenicity (ADA) assays: Serum anti-ADC antibodies are detected before the first, second, third and fourth doses, with 1 mL of blood volume for each sample.

After the first efficacy evaluation, 3 mL of blood is collected before each administration to detect serum ADC, TAb, free MMAE and serum anti-ADC antibodies.

**Method of Blood Collection**: Please refer to RC48-ADC Blood Sampling Procedure for specific details.

**Unexpected Blood Collection for PK:** A blood sample of 2 mL is taken for PK analysis immediately no later than 72 hours after SAEs, DLT or dosing suspension.

The above blood samples will also be used for exploratory purposes.

In consideration of rationality and operability, deviations in PK blood collection time points are permitted, as detailed below:

Prior to medication through 6 h postdose: Scheduled time point±3 min.

6 h postdose through 24 h postdose: Scheduled time point±10 min.

24 h postdose through 21 days postdose: Scheduled time point±30 min.

On July 4, 2017, blood collection time frame was adjusted in consideration of accuracy of PK analysis and clinical practice, as detailed below:

Prior to medication: In 30 min.

0.25 h after medication starts through end of drip: ±3 min.

1.5 h after medication starts: ±5 min.

12 h after medication starts: ±30 min.

24 h after medication starts: 1 h Prior to the next dose: ±2 h.

# 7. Outcome Measures

(1) Demographic data: Age, sex, height, and body weight.

(2) Vital signs: Respiration, sitting blood pressure, sitting pulse, and body temperature.

(3) Physical examination: Nervous system, mental system, respiratory system, cardiovascular system, gastroenteric system, and skin. ECOG evaluation;

(4) Laboratory test

Table 4 lists items of laboratory test to be performed during the study. For specific test time points, please see Appendix 2 Trial Flow Chart.

**Table 4. Laboratory test items.**

| **Item Name** | **Examination Including** |
| --- | --- |
| Virology test | 5 liver function tests, HCV, and HIV |
| Routine blood test | RBC, Hb, WBC, NEUT, EO, BA, LY, and PLT |
| Blood chemistry | Myocardial zymogram (CK-MB), cTn, GGT, ALT, AST, ALP, TP, ALB,  ALB/GLO, TBIL, IBIL, ^DBIL,^ BUN, Cr, Glu, K+, Na^+^, Cl^－^, LDH, TC, TG, HDL-C, and LDL-C |
| Blood coagulation test | International Normalized Ratio (INR), prothrombin time (PT), and Activated partial thromboplastin time (APTT) |
| Routine urinalysis + urine pregnancy test | SG, pH, U-PRO, U-GLU, U-RBC, U-WBC, and HCG pregnancy test (for females during child-bearing period) |
| PK★ | Total antibody in serum, ADC and free MMAE Plasma concentration |
| Immunogenicity★ | Serum anti-ADC antibody (ADA, anti-RC48-ADC antibody) |
| Tumor markers | Specific test items will be determined by the investigators depending on the tumor type |

★ Blood samples for PK and immunogenicity assays shall be handled as per blood sample handling procedure, and then sent by the sponsor to a designated laboratory for assay.

(5) Imaging Examination

At baseline, all patients with solid tumors must undergo CT/MRI scans of the chest, abdomen and pelvis according to the RECIST v1.1. For osteopathy patients, confirmatory bone scan shall be carried out at the time of baseline. Tumor assessment will be performed by using CT/MRI/radiography.

(6) ECG: ECG (12-lead) examination will be conducted 3 times with an interval of 5 min prior to each dose.

ECG monitoring will be carried out during medication.

ECG (12-lead) examination will be conducted 3 times with an interval of 5 min within 1-2 h postdose.

(7) Efficacy Evaluation

Objective Response Rate (ORR) refers to a percentage of patients whose tumors have shrunk to a given volume and maintained for a period, it includes PR+CR (as per RECIST 1.1).

At the dose-escalation trial phase, efficacy in each patient will be evaluated after every 2 treatment cycles, wherein one treatment cycle consists of 2 doses. For patients dosed Q3W, efficacy evaluation will be conducted after every 3 treatment cycles, wherein one treatment cycle consists of 1 dose.

At the extended trial phase, efficacy evaluation will be carried out for each subject according to the following evaluation criteria every 2 treatment cycles. ORR will be calculated on the basis of result of this evaluation.

For subjects rated as CR or PR at the initial evaluation, their efficacy should be verified 4 weeks later.

Improvement or worsening of general conditions will be expressed by variation in ECOG score before and after treatment.

**Evaluation Criteria**

In accordance with RECIST 1.1, objective evaluation criteria for antineoplastic drugs are established as follows:

**Complete Response (CR)** All target lesions disappear, and minor diameter of any pathological lymph node (including target and non-target nodes) shall be decrease by < 10 mm.

**Partial Response (PR)** Sum of diameters of target lesions is less than baseline level at least by 30%.

**Stable Disease (SD)** Target lesions neither shrink to a level of PR nor enlarge to a level of PD, and the minimum of sum of their diameters can serve as a reference value for the study.

**Progressive Disease (PD)** With the minimum of sum of diameters of all measured target lesions throughout the study as a reference, relative increase in sum of diameters is at least 20% (a baseline measurement will serve as the reference if it is the smallest value); in addition, absolute value of sum of diameters has to increase by at least 5 mm (occurrence of one or more new lesions is deemed PD, too).

(8) Safety and Tolerance Assessment

Body weight of each patient is to be measured prior to each treatment, which will be used in PK calculation.

When being subjected to blood pressure and pulse measurement, a subject must rest in sitting position for at least 3 min. Each measurement of blood pressure shall be taken on the same arm.

Result of 12-lead ECG will be recorded during the trial. The records include ECG date and time, heart rate, PR interval, QT interval, QTcB duration, and QRS duration. In overall evaluation, whether or not any clinically significant abnormality is found shall be defined. Clinically significant abnormalities, if any, have to be further defined. Signed original ECGs will be archived in the study site.

If laboratory test results of a patient during the screening period are out of a range specified in the protocol, then they can be reviewed once prior to enrollment. If the review result is still out of specification range, then this patient cannot be included in the trial.

If the result of a laboratory test of a patient during the screening period is out of normal range but this test is not specified in the protocol, then it will be judged by the investigators whether it is clinically significant or not. This test may be repeated once as soon as possible in a bid to eliminate laboratory error.

If there occurs a QTcF interval > 500 ms or increasing by > 60 ms relative of the baseline or grade III atrioventricular block during trial, then the trial medication should be suspended. The investigators will decide whether or not to continue medication after defining its cause.

In a non-DLT observation cycle, if any clinically significant AE occurring in one subject is judged by the investigators to be potentially, probably or definitely relevant to the study drug, and this AE is likely to continue or be potentially aggravated in the next scheduled dosing, then the investigators may postpone dosing or stop one dose of the study drug, the delay may last for up to 21 days or dose level may be lowered by 1 or 2.

The following provisions apply to toxic reaction occurring beyond DLT evaluation window.

Non-Hematological Toxicity

If any grade 4 non-hematological toxicity occurs in a patient postdose, or there occurs any important injury of organs such as lungs, cardiovascular system, liver and kidneys, then treatment of the patient should be immediately terminated.

If a patient gets any clinical benefit after treatment, but exhibits over grade 3 non-hematological toxicity (except hair loss, nausea, and emesis) and can be recovered to normal, grade 1 or baseline level in 14 days after the optimal supportive therapy, then one dose reduction is allowed. The specific dose shall be determined by the investigators and the sponsor through mutual negotiation (it may be a previous dose subjected to safety assessment or an arbitrary dose previously assessed). If the subject fails to be recovered to normal, grade 1 or baseline level in 14 days, then the investigators and the sponsor will discuss and decide whether or not to terminate the study treatment. If a toxic reaction occurs at the first dose level, then the dose can be reduced to 50%. Subsequent dose escalation trial will be adjusted by the investigators and the sponsor through negotiation.

Hematological Toxicity

If a patient gets any clinical benefits after treatment but exhibits grade 4 neutropenia, neutropenic fever, thrombocytopenia, anemia or grade 3 thrombocytopenia with bleeding tendency, then trial medication should be suspended until the toxic reactions are recovered to normal level or Grade 1. If toxicity is recovered to normal or grade 1 level in 14 days, then one dose reduction is allowed to continue treatment; if toxicity fails to be recovered to normal or grade 1 level in 14 days, then the investigators and the sponsor will discuss and decide whether or not to continue the treatment, one dose reduction will be conducted if continued patient treatment is allowed.

Toxicity Relapse

If a patient exhibits again the same grade 3 or above non-hematological toxicity or grade 4 hematological toxicity after a dose reduction, then treatment of the patient shall be stopped.

# 8. Study Procedure

Before the study begins, the patient must read and sign a current informed consent form approved by the Ethics Committee (EC). All study steps have to be carried out within time frames designated in the study plan.

## 8.1 Screening Period

Unless otherwise specified, the following steps have to be completed in 28 days before starting the treatment with the study drug:

- Case history and demographic data acquisition, and recording (by the investigators) identity card, correspondence address, and phone number of every patient, though such information will not be recorded in a CRF;
- Detailed inquiry of case history, and detailed comprehensive physical examination: ECOG score, height, body weight, vital signs, and checkup of various organs;
- Routine blood test, and routine urinalysis;
- Blood chemistry (myocardial zymogram, cardiac troponin, GGT, ALT, AST, ALP, TP, ALB, ALB/GLO, TBIL, IBIL, DBIL, BUN, Cr, Glu, K^+^, Na^+^, Cl^－^, LDH, TC, TG, HDL-C, and LDL-C);
- Blood coagulation tests (INR, APTT, and PT);
- HER2 protein expression and gene amplification assays: immunohistochemistry (ICH), and fluorescence in situ hybridization (FISH);
- Tumor markers (specific test items will be determined by the investigators depending on the tumor type);
- Virology test (HIV, five HBV tests, and HCV);
- Electrocardiographic examination for 3 times at intervals of 5 minutes, particularly observing QT, QTc and P-R intervals (within 7 days before the initiation of the trial);
- Echocardiographic examination to observe LVEF (within 28 days before the initiation of the trial);
- CT or MRI scans of the chest, abdomen, pelvis, brain and other relevant lesions (within 28 days before the initiation of the trial);
- Urine HCG tests for female patients of childbearing age to exclude pregnant subjects;
- Assessment of current concomitant medications and treatments;
- Assessment of current symptoms.

Written informed consent forms must be obtained before any of the study-related medical procedures. CT/MRI scan results obtained prior to the signing of informed consent forms can be used for the tumor assessments during screening (examination must be made within 28 days before the initiation of the trial).

## 8.2 Trial Period

The following procedures should be completed in accordance with the trial schedule:

- Perform physical examination and ECOG evaluation for every cycle;
- Examine patients for vital signs every medication cycle, measure body weight before each administration, and monitor vital signs at the point of pharmacokinetic blood collection during the initial administration;
- Examine the following items before each administration: routine blood test, routine urinalysis, blood chemistry (Troponin, GGT, ALT, AST, ALP, TP, ALB, ALB/GLO, TBIL, IBIL,DBIL, BUN, Cr, Glu, K+, Na+, Cl－, LDH, TC, TG, HDL-C, and LDL-C);
- Test blood coagulation function (INR, APTT and PT) once every treatment cycle;
- Tumor markers (specific test items will be determined by the investigators depending on the tumor type);
- Perform ECG examination 3 times at intervals of 5 minutes before each administration, monitor ECG during medication, and perform ECG examination 3 times at intervals of 5 minutes within 1-2 h after administration. If the subject experiences precordial chest pain, palpitations or other symptoms, or has an abnormal ECG, CK-MB test and echocardiography may be added;
- Perform tumor imaging examination, including CT or MRI scans of the chest, abdomen, pelvis, brain and other related lesions:
  - wherein, for subjects rated as CR or PR at the initial evaluation, their efficacy should be verified 4 weeks later, and tumor assessment after verification should follow the previously established examination time points;
  - when the treatment ends or subject withdraws (if no tumor assessment has been performed within the preceding 4 weeks).
- Record adverse events: from Day 1 of treatment to at least 21 days after the last dosing, or until all severe or drug-related toxic reactions are safely eliminated or returned to Grade 1 as specified in NCI CTCAE 4.0. In addition, observe and record clinical manifestations during medication;
- Record concomitant medications or treatments: from 4 weeks before the initiation of the study to at least 21 days after the last dosing.

## 8.3 Extended Trial

Patients evaluated as CR, PR and SD in the first efficacy evaluation during dose escalation phase can enter the extended trial on a voluntary basis, provided that investigators estimate that continuous medication would be beneficial to the patient. A time frame of ±3 days is allowed for all tests (except imaging examination) for each visit. An extended trial should include the following tests:

- ECOG evaluation;
- Body weight measurement, physical examination and vital sign examination for 1 time;
- Examine the following items before each administration: routine blood test, routine urinalysis, blood chemistry (Troponin, GGT, ALT, AST, ALP, TP, ALB, ALB/GLO, TBIL, IBIL,DBIL, BUN, Cr, Glu, K+, Na+, Cl－, LDH, TC, TG, HDL-C, and LDL-C);

On July 4, 2017, the investigators and sponsor decided to discontinue the original dose-escalation study starting with 2.0 mg/kg Q3W. Instead, a dose-escalation study with an initial dose of 1.25 mg/kg QW was added. Subjects participating in the extended trial should undergo routine blood test, routine urinalysis, blood biochemical examination before the initial administration of each treatment cycle;

- Tumor markers (specific test items will be determined by the investigators depending on the tumor type);
- ECG examination before each administration; if the subject experiences precordial chest pain, palpitations or other symptoms, or has an abnormal ECG, CK-MB test and echocardiography may be added;

On July 4, 2017, the investigators and sponsor decided to discontinue the original dose-escalation study starting with 2.0 mg/kg Q3W. Instead, a dose-escalation study with an initial dose of 1.25 mg/kg QW was added. Subjects participating in the extended trial should undergo ECG examination before the initial administration of each treatment cycle. If the subject experiences precordial chest pain, palpitations or other symptoms, or has an abnormal ECG, CK-MB test and echocardiography may be added;

- Tumor imaging examination, including CT or MRI scans of the chest, abdomen, pelvis, brain and other related lesions. For subjects rated as CR or PR at the initial evaluation, their efficacy should be verified 4 weeks later. A time frame of ±7 days is allowed. Specific evaluation time points are as follows:
  - once every 2 treatment cycles;
  - when the treatment ends or subject withdraws.

A 3 mL fasting blood sample is collected before each administration on the day of tumor evaluation (on the day of imaging examination) during the 3rd-8th treatment cycles. Samples are used for the detection of serum TAb, ADC, plasma free MMAE concentration, as well as serum anti-ADC antibodies (ADA, anti-RC48-ADC antibodies) to investigate PK/PD correlation and evaluate the relationship between trough concentration and efficacy.

- Record adverse events: until at least 21 days after the last dosing;
- Record concomitant medications or treatments: until at least 21 days after the last dosing.

## 8.4 Extended Trial

On July 4, 2017, the investigators and sponsor decided to let 3-5 study sites participate in the extended trial phase based on the current study results. At least 10 patients with HER2 ICH 2+/FISH- and at least 20 patients with HER ICH 2+/FISH+ or ICH 3+ with advanced solid tumors (gastric cancer is preferable) are enrolled and administered at 2.0 mg/kg Q2W until disease progression or intolerable adverse reactions. A treatment cycle consists of 2 doses. Efficacy is evaluated once every 2 cycles. Specific items are shown in Appendix 4 Flow Chart of Extended Trial.

## 8.5 Conclusion/Withdrawal

At the end of the treatment or in case of withdrawal, the following items should be tested if the patient has not undergone the examinations within 21 days before the study ends:

- Comprehensive physical examination: ECOG performance status, body weight and vital signs;
- Routine blood test, and routine urinalysis;
- Liver function, renal function, blood glucose, blood lipids and blood electrolytes;
- Urine HCG of female subjects of childbearing age;
- 12-lead electrocardiogram, echocardiogram;
- CT or MRI scans of the chest, abdomen, pelvis, brain and other relevant lesions (if has not been assessed within the preceding 3 weeks);
- Assessment of adverse events;
- Assessment of concomitant medications/treatments.

# 9 Clinical Observation

Enrolled subjects are assigned to wards and specialized personnel. Subjects are hospitalized throughout the pharmacokinetic data collection period. Tolerability, adverse events and general conditions experienced by subjects are observed under clinical supervision. Any serious adverse events should be dealt with appropriate emergency measures and treatments.

Any adverse events occurring during the clinical trial in all subjects, including abnormal clinical symptoms and vital signs and abnormalities in laboratory tests, should be carefully observed and recorded for clinical performance characteristics, severity, time of occurrence, duration, treatment methods and prognosis, and determined for their relevance to the investigational drug. The safety of the drug is evaluated according to NCI CTCAE v4.0.

During a non-DLT observation period, if a subject experiences a clinically significant adverse event for which investigators determine there is a possible, highly prossible or definite relation to the drug, and such adverse events may continue or worsen during the next planned administration, investigators may postpone or stop one administration. The delay time should not exceed 3 weeks.

For subjects experienced serious adverse events within 8 weeks after completion of follow-up assessments, additional follow-ups should be conducted and recorded. When no serious adverse events occur, this trial ends and investigators will not record subjects' subsequent clinical changes.

Each subject is assigned a unique serial number made up of the study site code (e.g. BZ for Beijing Cancer Hospital) and the subject's code. The subject's code is a 3-digit serial number assigned based on the order in which subjects signed the informed consent form. For example, the first patient who signed the informed consent form is designated as BZ001, and so forth. Assigned subject codes will not be reused. In addition, the study site should keep a copy of the source documents which record the subject codes and corresponding personal information.

# 10 Concomitant Medication

If investigators believe that the patient's condition need a particular drug, this should be recorded in the concomitant medication log in detail.

Modification in previous drug dose should also be recorded in the concomitant medication log.

## 10.1 Unavailable Drugs During Study

Anticancer drugs, and assistant drugs used in cancer therapy such as anticancer traditional Chinese medicines and immunological agents should not be used during the trial.

## 10.2 Drugs may be Conditionally Used During Study

When non-DLT adverse events occur during the DLT observation period of the dose escalation phase, no medical treatment (including but not limited to dosing suspension and symptomatic treatment) should be provided in principle in order to observe possible adverse reactions (including severity and reversibility) to the investigational drug. Once any DLT toxic reactions specified in the protocol occur, medication must be discontinued immediately and active treatment must be provided. Medications should be recorded on CRF. Adverse events occurring during the non-DLT observation period should be dealt according to "(8) Safety and Tolerability Evaluation" of outcome measures described in Protocol 7.

On July 4, 2017, the investigators and sponsor determined that during the dose escalation phase starting with 2.5 mg/kg Q2W and dose-escalation trial with an initial dose of 1.25 mg/kg QW, adverse events occurred within the DLT observation period may be dealt with symptomatic treatments as necessary upon investigators' judgment; G-CSF treatment is allowed for Grade 4 neutropenia but prohibited for preventive application. Once any DLT toxic reactions specified in the protocol occur, medication must be discontinued immediately and active treatment must be provided. Medications should be recorded on CRF.

In principle, medication should not be discontinued when a subject experiences a toxic reaction during the extended trial. Doctors may administer appropriate treatment according to the specific situation based on the following principles:

Doses are escalated in an order of 2 mg/kg, 1.5 mg/kg, 1 mg/kg and 0.5 mg/kg, with 0.5 mg/kg as the lowest dose.

Myelosuppression: In case of myelosuppression, investigators may perform symptomatic treatments based on clinical manifestations. If Grade III or higher neutropenia occurs, GSF treatment should be provided. Specific dose modification is shown in Table 5.

**Table 5. Dose modification criteria for myelosuppression.**

| **Myelosuppression** | **Treatment Adjustment** | **Dose Modification for Next Course of Treatment** |
| --- | --- | --- |
| Grade I and II | Maintain original dose | Maintain original dose |
| Grade III neutropenia/Grade III thrombocytopenia | | |
| First occurrence | Delay medication until recovered to ≤ Grade I | Maintain original dose |
| Second occurrence | Delay medication until recovered to ≤ Grade I | Decrease by 1 dose level |
| Grade IV neutropenia/Grade III neutropenia with fever/Grade IV thrombocytopenia | | |
| First occurrence | Delay medication until recovered to ≤ Grade I | Decrease by 1 dose level |
| Second occurrence | Discontinue medication |  |

Impaired liver function: When the patient experiences liver function damage, treatment of liver protection may be administered and preventative measures can be provided in subsequent treatments. Specific dose modification is shown in Table 6.

Neurotoxicity: When patients develop neurotoxicity, preventative medication may be administered in subsequent treatments. Specific dose modification is shown in Table 6.

Diarrhea: When the patient experiences loose stools or diarrhea, antidiarrheal medication is recommended. Specific dose modification is shown in Table 6.

**Table 6. Dose modification criteria for impaired liver function, neurotoxicity and diarrhea.**

| **ALT and AST abnormalities**  **\Neurotoxicity\Diarrhea** | **Treatment Adjustment** | **Dose Modification for Next Course of Treatment** |
| --- | --- | --- |
| Grade I and II | Maintain original dose | Maintain original dose |
| ≥ Grade III | | |
| First occurrence | Delay medication until recovered to ≤ Grade I | Maintain original dose |
| Second occurrence | Delay medication until recovered to ≤ Grade I | Decrease by 1 dose level |
| Third occurrence | Discontinue medication |  |

Rashes: Symptomatic treatments may be applied when rashes occur. Specific dose modification is shown in Table 7.

**Table 7. Dose modification criteria for rashes.**

| **Rashes** | **Treatment Adjustment** | **Dose Modification for Next Course of Treatment** |
| --- | --- | --- |
| Grade I and II | Maintain original dose | Maintain original dose |
| ≥ Grade III | | |
| First occurrence | Delay medication until recovered to ≤ Grade II | Decrease by 1 dose level |
| Second occurrence | Delay medication until recovered to ≤ Grade II | Decrease by 2 dose level |
| Third occurrence | Delay medication until recovered to ≤ Grade II | Lowest dose of 0.5 mg/kg |
| Fourth occurrence | Discontinue medication |  |

Gastrointestinal reaction: Anti-vomiting drugs are not recommended for prevention purpose. Antiemetic therapy may be given when vomiting occurs. When vomiting is ≥ Grade II, prophylactic antiemetics may be given during subsequent administration. Medication is not required to delay.

Hair loss: Medication is not required to delay.

Other toxicities: Investigators should treat other toxic reactions based on clinical manifestations. If the AEs are still ≥ Grade II after treatment, it is necessary to delay medication until recovered to ≤ Grade I. Specific dose modification is shown in Table 8.

Drugs used in the above treatments are recorded on the CRF and the delays in administration will not affect the time points for tumor evaluation.

**Table 8. Dose modification criteria for other toxic reactions.**

| **NCI CTCAE (4.0)** | **Treatment Adjustment** | **Dose Modification for Next Course of Treatment** |
| --- | --- | --- |
| Grade I | Maintain original dose | Maintain original dose |
| Grade II | | |
| First occurrence | Delay medication until recovered to ≤ Grade I | Decrease by 1 dose level |
| Second occurrence | Delay medication until recovered to ≤ Grade I | Decrease by 2 dose level |
| Third occurrence | Discontinue medication |  |
| Grade III | | |
| First occurrence | Delay medication until recovered to ≤ Grade I | Decrease by 1 dose level |
| Second occurrence | Discontinue medication |  |
| Grade IV | | |
| First occurrence | Discontinue medication |  |

**The following drugs should be applied with caution during the study:**

It would be best to avoid combined application with inhibitors or similar products that can affect the metabolism of CYP isozymes (CYP3A4 and CYP3A5). If there is a need for the combined application with inhibitors or similar products that can affect the metabolism of CYP isoenzymes (CYP3A4, CYP3A5), adverse reactions in subjects must be closely monitored during the trial.

The action mechanism of RC48-ADC is: MMAE is carried to target organs by antibodies, free MMAE is released by enzymic action and affects the mitosis of cancer cells to promot apoptosis. This leads to a low level of free MMAE in blood. Increment or reduction in MMAE exposure levels caused by the combined application with CYP3A4 inhibitors or inducers is relatively small, therefore there is no need to adjust the dosage in such a combined application. However, RC48-ADC administration may be delayed if serious adverse events occur.

Studies using SGN-35 as a reference standard suggested that MMAE, a substrate of CYP3A4 and/or P-glycoprotein, is predominantly metabolized by CYP3A. Strong CYP3A4 inhibitors such as ketoconazole, itraconazole, clarithromycin, atazanavir, indinavir, nefazodone, nelfinavir, ritonavir, saquinavir, telithromycin and voriconazole should not be used during the trial medication.

# 11 Recording, Identification and Handling of Adverse Effects

## 11.1 Recording of Adverse Effects

All adverse events that occur during the study should be recorded in detail, wherein accurate information of the occurrence time, severity, duration, response measures and outcome should be included. Adverse events occurring during the study should be analyzed and identified carefully. Appropriate measures should be taken as necessary. Follow-ups should be conducted for any abnormal symptoms, signs, laboratory test results or other special findings observed during the study.

Adverse events include all occurrences or exacerbations of adverse signs, symptoms or medical conditions associated with or unrelated to the investigational drug during the trial medication. Medical conditions/diseases that occur prior to the trial treatment should be recorded as adverse events only if they deteriorate after the initiation of the trial medication. Abnormal laboratory results are considered as adverse events only when they lead to clinical symptoms or signs, have clinical significance, or require medical intervention.

A serious adverse event is an adverse event that causes significant harm to the subject. It includes but not limited to:

a. AE leading to death

b. Life-threatening AE

c. AE causing permanent or temporary damage to organs and functions

d. AE resulting in hospitalization or prolonged hospitalization. Inpatients with the following conditions are not determined as having serious adverse events:

● Hospitalization for routine treatment or trials, unrelated to adverse events

● Hospitalization for follow-up treatments after disease progression

● Hospitalization for elective or pre-planned treatments of pre-existing diseases unrelated to the trial; the pre-existing diseases have not worsened after the initiation of trial medication

● Admission to hospital for social reasons or rehabilitation; no deterioration of subject's general condition

e. AE causing congenital anomalies or birth defects

The occurrence, development and outcome of all adverse events should be recorded in the adverse event observation table and followed up until normal levels (based on clinical determination) or other outcomes are obtained. Drugs used during the treatment of adverse events must be detailed in the concomitant medication log.

When a serious adverse event occurs, the subject should stop receiving the investigational drug and receive appropriate treatment immediately. The reason of the SAE should be clarified.

**(1) Reporting method**: If any serious adverse events occur during the trial, immediately discontinue medication and report to the sponsor, ethics committee of the study site, provincial and municipal food and drug administration departments, National Health and Family Planning Commission of the People's Republic of China, and CFDA Division of Drug Research Supervision (within 24 hours).

**Table 9. Organizations for serious adverse event reporting.**

| **Reporting Organization** | **Contact No.** | **Contact Person** |
| --- | --- | --- |
| Beijing Cancer Hospital | 010-88196391 |  |
| RemeGen., Ltd. | 010-58075561 | Li Xiaofeng |
| Department of Drug Registration, China Food and Drug Administration | 010-88363228 |  |
| Bureau of Medical Administration, National Health and Family Planning Commission of the People's Republic of China | 010-68792734 |  |
| Center for Drug Evaluation, Beijing Food and Drug Administration | 010-83560723 |  |

(2) Treatment measures: Emergencies in patients should be dealt with according to the medication and clinical manifestations. Clinical research associates (CRA) should be notified of the treatment outcome. Investigators should record details including date, treatment process and outcome on the Case Report Form (CRF) and sign.

**(3) Follow-up**: To ensure their safety, subjects who underwent adverse events should be followed up within 1 month (if possible) after the AEs are treated, and should be regularly examined until recovery to normal levels or investigators determine that the condition is stable. If investigators determine that follow-up is unnecessary or in the case of loss to follow-up at the end of the study, an explanation should be recorded in the study documentation.

## 11.2 Identification of Adverse Events

A causal relationship between adverse events and drugs can be analyzed based on the following aspects: (1). Is there a reasonable sequential relationship between the time of first dosing and occurrence of suspected adverse events? (2). Does the suspected adverse event belong to any known adverse events of this drug? (3). Can the suspected adverse event be attributed to effects of concomitant medication, patient's clinical status or other therapies? (4) Is the suspected adverse event eliminated or mitigated after termination or reduction of medication? (5). Are there any similar reactions after re-exposure to the suspected drug? The following 5-tier classification criteria should be used to assess the relationship between adverse events and drugs: 1) definitely related; 2) possibly related; 3) definitely unrelated; 4) possibly unrelated; 5) unable to determine.

**Table 10. Classification criteria for adverse events.**

|  | (1) | (2) | (3) | (4) | (5) |
| --- | --- | --- | --- | --- | --- |
| Definitely related | **+** | **+** | **-** | **+** | **+** |
| Possibly related | **+** | **+** | **-** | **+** | **?** |
| Unable to determine | **+** | **+** | **±** | **±** | **?** |
| Possibly unrelated | **+** | **-** | **±** | **±** | **?** |
| Definitely unrelated | **-** | **-** | **+** | **-** | **-** |

Notes: + Yes; - No; ± Undetermined or no; ? Unknown.

The relationship between adverse events and test drugs was assessed according to the five-level classification criteria. The details of the progression, degree, treatment and outcome of adverse events are recorded on the observation form.

## 11.3 Possible Adverse Events and Rescue Measures

Preclinical test results: No obvious abnormal reaction was seen in the nude antibody control group during the toxicity test in rats and cynomolgus monkeys, and the toxic reactions to RC48-ADC were basically the same as those to MMAE control. It is therefore expected that the adverse effects of RC48-ADC are mainly related to the cytotoxic effects of MMAE.

The specific findings of the animal safety study were as follows:

Myelosuppression and Immunosuppression: During the administration period, the blood cell counts of the animals in each administration group showed a significant decrease in granulocytieand erythrocyte and platelet counts. Histopathological examination revealed reduction of sternal bone marrow hematopoietic cells, thymus atrophy, submandibular, mesentery and inguinal lymph node atrophy and white pulp spleen atrophy. The most obvious changes were in rats in the MMAE control group and the 24 mg/kg trial drug group, probably due to the inhibitory effects of MMAE on proliferation of bone marrow hematopoietic and immune system cells.

Digestive Tract Injury: Perianal contamination symptoms were observed in individual rats of the MMAE control group and the 24 mg/kg trial drug group during the test period. Pathological examination revealed abnormal necrosis, erosion, hemorrhage, hyperplasia, etc. in the digestive tract mucosa of RC48-ADC animals and individual euthanized animals. Some animals also had bacterial infections but recovered by the end of the 6-week recovery period, possibly due to damage to the digestive tract epithelial cells related to MMAE. No obvious reaction was observed in cynomolgus monkeys.

Hepatotoxicity: During the test period, TP and A/G were reduced in the animals administered with the trial drug. Pathological examination revealed focal necrosis of the liver in the MMAE group and the trial drug group. Although their blood chemistry did not show any increase in liver-related parameters such as ALT or AST during the test period, the pathology findings indicated that MMAE and the trial drug produced a toxic reaction in the liver.

Reproductive Toxicity: In the repeated dose toxicity trial in rats, varying degrees of testicular and epididymal atrophy were seen in both dead and euthanized animals in the MMAE and trial drug groups. By the end of the recovery period, some animals had not yet recovered. There were no significant changes in the cynomolgus monkey experiment.

Dermal Toxicity: In repeated dose toxicity experiments in rats, the MMAE and 24 mg/kg trial drug groups showed scattered scab sites in individual animals. Considered along with acute toxicological findings in rats, it is possible the lesions are caused by dermal toxicity related to MMAE.

In reference to the SGN-35 clinical trial literature, the most common adverse reactions are peripheral sensory neuropathy, fatigue, nausea, diarrhea, joint pain, fever, loss of appetite, muscle pain and upper respiratory infections. The severity of most adverse events is mild to moderate (I or II) [10].

Inclusion criteria are defined with some particular restrictions in reference to the above findings. For example, peripheral sensory neuropathy is also one of the expected adverse effects of MMAE (antimicrotubule drug). Therefore, the fifteenth item of the exclusion criteria for patients is: Patients with peripheral neuropathy of grade ≥ 2 are ineligible. The dosage of patients who experienced novel or exacerbated peripheral sensory neuropathy during the trial was reduced 1-2 levels. If the peripheral sensory neuropathy could not be resolved, the trial was stopped.

Treatment: Adverse reactions generally do not require treatment, but disappear after administration is discontinued.

Rescue medications: 0.1% epinephrine, promethazine, dexamethasone, hydrocortisone, nikethamide, and 5%~10% glucose saline.

Rescue equipment: Aspirator, tracheal intubation, ventilator, EKG monitor, defibrillator, etc.

# 12. End of Study

The study ends when the last subject has been dosed for 2 consecutive cycles or in case the disease progresses or the toxicity becomes intolerable, whichever occurs first. Patients entered the extended trial phase continue with the study in accordance with the protocol.

# 13. Observation and Records

## 13.1 Recording of Data

Charts for the subjects' physical examination screenings, blood concentration measurements and observation records must be timely, accurate, complete, standardized and authentic. When making any substantiated corrections to recorded observations, they may only be crossed out, not erased or overwritten. Corrections are written in the margins and must be signed and dated by the investigator. There are raw data records when analyzing serum samples.

## 13.2 Data Monitoring

CRAs review the study records and confirm that the data records are accurate, standardized, complete, and authentic. CRAs write a "Clinical Trial Audit Report" after each visit.

## 13.3 Data Archiving

After the conclusion of the trial, the original study data will be archived at the National Institute of Drug Clinical Trials, Beijing Cancer Hospital.

# 14. Data Management

**14.1 Digital Data Management**

1. An electronic data management system (DAS for EDC) was used for this study.

2. Study medical record design: Data collection forms were designed and the study process, titles of data forms, and data items were defined according to the protocol. Corresponding data collection guidelines were also be developed. The design was finalized after being reviewed and approved by the sponsor.

3. Electronic Case Report Form (eCRF) Construction: The data administrator constructed the eCRF based on the study protocol and study medical records.

4. The EDC system utilizes dual control of role permissions. All users who access the EDC must fill in the user account application form. After confirmation and approval by the sponsor, the system administrator creates the project administrator account and grants permissions. The project administrator applies to create accounts for investigators, CRCs, CRAs, auditors and data administrator, and grant different permissions to access the EDC. For example, investigators can only access the content from their own study site and have permission to amend the data; the sponsor is limited to browsing the EDC; CRAs can access the EDC data from various sites without authority to revise the data, but they can submit queries.

5. Data entry: The primary investigator assigns a CRC, and the CRC promptly and accurately enters the study’s case records into the eCRF.

6. Data Verification: As data is being entered, the EDC performs logical verification of data entry and issues system queries in real time. In addition to system queries, the data administrator conducts manual verification of data and submits queries if there is an issue.

7. Source Data On-Site Verification (SDV): The CRA logs into DAS for EDC at each study site to verify the 100% consistency of source data such as eCRF data and study medical records, and can submit queries online at any time problems are discovered.

8. Response to Queries: Investigators can answer questions online in real time, or download a list of queries, which are answered offline, then recorded in the EDC by the CRC. Data administrators and CRAs review the investigators’ responses and, if necessary, reissue the queries until the data is “clean”.

9. Data locking and exporting: After all subjects have completed the trial, all medical records are entered into the system, and the data is reviewed and confirmed by the principal investigator, sponsor, statisticians, and data managers, the data administrator lock the data. After all the data is locked, the data administrator imports it into the designated database to be analyzed by the statistician. The data cannot be edited once locked. Problems found after data locking can be corrected in the statistical analysis program after they are confirmed. Once the data is locked, if there is definite evidence demonstrating it must be unlocked, the investigator and sponsor must sign the relevant documents.

10. eCRF archive: At the conclusion of the trial, an eCRF for each subject is generated in the format of PDF and record onto a disc. These files should be retained at corresponding responsible research organization up to 5 years after the drug is marketed.

11. EDC deactivation: After the trial is over, the data administrator puts forward the application for EDC deactivation and cancels all account access after obtaining the permission of the sponsor. After the data is entirely backed up, the EDC is shut down (ie offline). For 5 years after completion of the trial, the data management center is able to access the EDC system after making an appointment.

**14.2 Data Management Plan**

1. The data management plan was written by the data administrator.

2. The data management plan serves as a guide for the entire data management process, and all subsequent processes should follow the time and method defined therein.

3. The contents of the data management plan include:

(1) Administration of data management plans, such as ownership, cover design, table of contents, etc.

(2) The general situation of the study, such as its purpose, overall design, etc.

(3) Data management work schedule. The schedule should reflect the start and completion time of each step in the process. The schedule should also be coordinated with the overall timetable of the entire clinical trial.

(4) Allocation of users and permissions: Includes EDC system administrators (Admin), data managers (DMs), investigators, clinical research coordinators (CRCs), clinical research associates (CRAs), etc.

(5) Data management design includes database design and logical verification design.

(6) Data processing regulations

(7) Data quality control

(8) EDC Deactivation

(9) Data security measures

(10) EDC system emergency plan

# 15. Statistical Analysis

## 15.1 Analytical Dataset

Safety set (SS): Actual data obtained from all subjects who were randomized and received 1 dose of study drug, and recorded with a security indicator. Data lacking safety values cannot be carried forward. The incidence of adverse reactions is expressed per number of cases in the SS.

Full Analysis Set (FAS): All subjects who were randomized and received 1 dose of study drug, and recorded with an efficacy indicator.

PK concentration data set: All subjects who were randomized and received 1 dose of study drug, and had at least 1 piece of blood concentration data of the study drug during the trial.

PK parameter analysis set: All subjects who were randomized and received 1 dose of study drug, and had at least 1 valid PK parameter of the study drug during the trial.

## 15.2 Safety Analysis

- Safety analysis is conducted using the SS.
- Due to the small number of subjects and the mainly descriptive statistics, professional analysis should be performed on the results.
- Qualitative indicators are described by frequency tables, percentages, or composition ratios; quantitative indicators are described by averages, standard deviations, or maximum, minimum and median.
- The incidence of adverse events and adverse reactions is calculated.
- The rate and frequency of adverse events and adverse reactions are listed by systems to calculate the percentage.
- Various adverse events are listed in detail.
- Various adverse reactions are listed in detail.
- The number of cases and rate of "conversion to abnormal" or "abnormality exacerbation" in laboratory indicators, ECG and physical examinations after the trial are recorded.
- Laboratory indicators, ECG, abnormal physical examinations and clinical interpretations are listed.
- The total incidence of adverse reactions in each group and the incidence and severity of adverse reactions in each system are investigated.
- Exploratory analysis is performed on the relationship of PK parameters with SAE and DLT.

## 15.3 Pharmacokinetic Analysis

A c-t curve is plotted for the blood concentration (c) vs time (t) for each subject listed in the PK concentration data set. In addition, a chart is made of the mean and standard deviation of the drug concentrations at various time points, and an average blood concentration curve is plotted with a standard deviation.

The noncompartmental method is used to analyze data in the PK parameter analysis set. Pharmacokinetic parameters of each subject and the mean and standard deviation of these parameters are calculated. The pharmacokinetic parameters are compared between the first and last drug administration and between different dose groups.

Pharmacokinetic properties are analyzed. The dose proportionality of C_max_ and AUC_0-∞_ after a single dose is analyzed by a power function model to evaluate the linear relationship of multiple doses.

Cmax The maximum plasma concentration observed

_T_max Time (an observation time point) at which the C_max_ is observed

Clast The last detectable plasma concentration

T_last_ Time (an observation time point) at which the C_last_ is observed

_k_el Elimination rate constant, based on a linear regression of the terminal elimination phase of the plasma concentration-time curve

T_1/2_λz The estimated terminal elimination half-life in plasma, calculated by dividing In2 by the terminal elimination rate constant (0.693/k_el_)

AUC_0-t_ The area under the plasma concentration-time curve between time 0 (before dosing) and the last time point of quantifiable detection (T_last_)

AUC_0-∞_ The area under the plasma concentration-time curve from time 0 (before dosing) extrapolated to infinity

% AUC_0-∞_ Percentage of AUC from 在AUC_0-t_ extrapolated to AUC_0-∞_

Vz/F Apparent volume of distribution in terminal elimination phase

CL/F Apparent clearance after drug administration; CL/F = dosage/AUC0-∞

## 15.4 Efficacy Analysis

FAS data is used for efficacy analysis.

Due to the small number of subjects and the mainly descriptive statistics, professional analysis should be performed on the results.

The number of cases, means, standard deviations, medians, minimums, and maximums are calculated for continuous variables.

Frequency and composition ratio are calculated for count and grade data.

For primary evaluation, the objective responses of subjects are evaluated in reference to the Response Evaluation Criteria in Solid Tumors (RECIST) (version 1.1). The objective response rate (the proportion of patients with confirmed complete response or partial response, according to RECIST) is calculated and described statistically.

Descriptive analysis is performed for other efficacy indicators and exploratory analysis of the relationship between pharmacokinetic parameters and efficacy is conducted.

## 15.5 Analysis Software and General Requirements

Safety analysis is done using SAS 9.3 software.

Pharmacokinetic analysis is done using WinNolin 6.1 and DAS 3.0, the results of which are cross-validated.

Detailed statistical methods are provided in the statistical analysis plan.

# 16. Ethical Requirements

## 16.1 Study documents should be approved by the ethics committee in advance

This clinical trial must be conducted in accordance with the Declaration of Helsinki (1996), the CFDA Good Clinical Practice (GCP) and related regulations.

Before the official start of the clinical trial, a study protocol (i.e., this protocol), which has been discussed, revised and signed by the investigators and sponsor, must be submitted to the ethics committee for approval. If any problems with this protocol occur during the implementation and require revision, the sponsor should be informed and, after consultation with all parties, the protocol should be revised by the responsible research organization, submitted in writing to the sponsor and each participant for endorsement, and re-approved by the ethics committee for implementation. If any problems with important new data concerning study drugs is found, the informed consent must be revised and submitted to the ethics committee for approval before being signed by subjects again.

## 16.2 Subjects must go through the informed consent process and sign informed consent forms before the clinical trials

Before the clinical trial begins, the investigator must provide the subjects with details about the clinical study, including the nature of the study, the purpose of the study, possible benefits and risks, alternative therapy available, and the subjects' rights and obligations according to the Declaration of Helsinki, etc. Clinical trials may only commence when the subject and his/her legal representative has fully understood and signed the informed consent form. Each subject is required to leave detailed contact information, including contact address, phone number and ID card information. Likewise, the doctor must provide his contact information to the patient so that the patient can contact the doctor at any time the patient's condition change. This aids the doctor in staying updated on such changes, and reminding the patient of return visits to avoid loss to follow-up.

## 16.3 Occurrence of any AEs in the trial should be able to be effectively treated and followed up

In case of occurrence of an adverse event during the trial, investigators may decide whether to suspend the trial depending on the situation. In case of a serious adverse event, the investigator will immediately proceed with proper treatment or rescue measures to protect the subject's safety. All adverse events will be followed up until they are properly resolved or stable. Different methods of follow-up, such as hospitalization, outpatient visit, home visit, phone call and communication may be employed based on the severity of adverse events, the distance between the subject's residence and the hospital, and the medical specialty of the center.

# 17. Usage and Publication of Information

All information about the investigational drug and previously unpublished data provided by the sponsor, such as patent applications, drug formulation, manufacturing processes, basic research data, and any information obtained in this trial, are confidential and the property of the sponsor. Investigators should ensure that they maintain confidentiality and only use this information to support this trial. It must not be used for other purposes without the written consent of the sponsor.

The sponsor has the right to publish the data and information of this trial without the permission of the investigator. If the investigator wishes to publish the information in this trial, the draft paper must be provided to the sponsor for review 60 days before its submission or presentation. A quick review of summaries, posters or other materials will be arranged. According to the written request of the sponsor (if any), the investigator promises to delay publication for 60 days, thus allowing time for the sponsor to apply for a patent or other intellectual property protection.

# 18. Content Added During the Trial

On Oct. 18, 2016, the investigators and sponsor decided to add a dose-escalation trial with Q3W administration in the dose-escalation phase. Starting from the 2.0 mg/kg dose group, 2-3 patients are enrolled. Pharmacokinetic data from Q3W administration are collected repeatedly. The dose next to 2.0 mg/kg is determined based on the results of the dose escalation study with Q2W administration. The extended trial is started with the 2.0 mg/kg dose group. Pharmacokinetic data from Q2W administration are collected repeatedly. The dose next to 2.0 mg/kg is determined by the investigators. Q2W dosing means 1 treatment cycle includes 2 doses. The efficacy evaluation is performed once every 2 treatment cycles.

On July 4, 2017, the investigators and sponsor decided that:

(1) Symptomatic supportive treatment is allowed in the DLT evaluation period. Dose escalation trials are performed according to the traditional 3+3 mode, wherein doses are proposed to be escalated from 2.5 mg/kg to 3.0 mg/kg, 3.5 mg/kg and 4.0 mg/kg with Q2W administration until MTD is reached, or the sponsor and investigators decide to terminate the escalation study.

(2) The original dose-escalation study starting with 2.0 mg/kg Q3W was discontinued. Instead, dose escalation trial is carried out with an initial dose of 1.25 mg/kg, followed by 1.5 mg/kg, in the traditional 3+3 mode with QW administration. Symptomatic supportive treatment is allowed in the DLT evaluation period until MTD is reached. If the 1.5 mg/kg dose group does not reach MTD, the sponsor and investigators will jointly decide on the next escalating doses or whether to terminate the dose escalation trial. The DLT for this phase is defined as follows:

DLT refers to the following toxic reactions observed in the DLT evaluation window (observation period of Day 1-21 after the first dose) that are considered related to RC48-ADC treatment by the investigators or sponsor, wherein the grading is based on the 5-level system of the CTCAE v4.0:

- Grade 4 neutropenia lasting more than 3 days after symptomatic treatment; or grade 4 neutropenia recurring after recovery to a normal level through symptomatic treatment.
- Neutropenic fever (defined as absolute neutrophil count [ANC] < 1000/mm3 accompanied by a fever higher than 38.3 °C or a fever above 38 °C that persists for more than 1 h).
- Grade 3 neutropenia with confirmed infections.
- Grade 3 thrombocytopenia with bleeding tendency.
- Grade 4 thrombocytopenia.
- Non-hematologic toxicity of Grade 3 or higher after supportive care. Except for nausea, vomiting and hair loss.
- Renal toxicity ≥ Grade 3.
- Neurological toxicities ≥ Level 2 that persist until the next administration.
- Cardiac toxicity ≥ Grade 2.
- Hepatic transaminases reach grade 2 at baseline, with a level ≥10×ULN.

(3) On July 4, 2017, the investigators and sponsor decided to let 3-5 study sites participate in the extended trial phase based on the current study results. At least 10 patients with HER2 ICH 2+/FISH- and at least 20 patients with HER ICH 2+/FISH+ or ICH 3+ with advanced solid tumors (gastric cancer is preferable) are enrolled and administered at 2.0 mg/kg Q2W until disease progression or intolerable adverse reactions.

# 19. References

1. Investigator's Brochure

2. CFDA, Technical Guidelines for Pharmacokinetic Study of Chemical Drugs in Human, March 2005

3. CFDA, Good Clinical Practice of Pharmaceutical Products

4. Jankowitz RC, Abraham J, Tan AR, et al. [Safety and efficacy of neratinib in combination with weekly paclitaxel and trastuzumab in women with metastatic HER2 positive breast cancer:an NSABP Foundation Research Program phase I study.](http://www.ncbi.nlm.nih.gov/pubmed/24077916) Cancer Chemother Pharmacol. 2013, 72(6):1205-1212

5. Beeram M, Krop IE, Burris HA, et al. [A phase 1 study of weekly dosing of trastuzumab emtansine (TDM-1) in patients with advanced human epidermal growth factor 2-positive breast cancer.](http://www.ncbi.nlm.nih.gov/pubmed/22648179)Cancer. 2012, 118(23):5733-5740

6. Krop IE, Beeram M, Modi S,et al. [Phase I study of trastuzumab-DM1, an HER2 antibody-drug conjugate, given every 3 weeks to patients with HER2-positive metastatic breast cancer.](http://www.ncbi.nlm.nih.gov/pubmed/20421541)J Clin Oncol. 2010, 28(16):2698-2704

7. CFDA, Guiding Principles for Clinical Trial Techniques of Anti-Tumor Drugs, May 2012

8. CFDA, Technical Guidelines for Non-Clinical Studies of Cytotoxic Anti-Cancer Drugs

9. FDA, Estimating the Maximum Safe Starting Dose in Initial Clinical Trials for Therapeutics in Adult Healthy Volunteers

10. Clinical Study Data of SGN-35 for FDA Marketing Application: http://www.accessdata.fda.gov/scripts/cder/drugsatfda/index.cfm?fuseaction=Search.Label_ApprovalHistory#apphist

# 20 History of Revision

Oct. 16, 2015 Revised Version 2.0

Feb. 1, 2016 Revised Version 3.0

Aug. 29, 2016 Revised Version 4.0

Oct. 20, 2016 Revised Version 5.0

July 4, 2017 Revised Version 6.0

Annex 1. ECOG Scale

| **Grade** | **ECOG Performance Status** |
| --- | --- |
| 0 | Fully active, able to carry on all pre-disease performance without restriction. |
| 1 | Restricted in physically strenuous activity but ambulatory and able to carry out work of a light or sedentary nature, e.g., light house work, office work. |
| 2 | Ambulatory and capable of all selfcare but unable to carry out any work activities; up and about more than 50% of waking hours. |
| 3 | Capable of only limited selfcare; confined to bed or chair more than 50% of waking hours. |
| 4 | Completely disabled; cannot carry on any selfcare; totally confined to bed or chair. |
| 5 | Dead. |

# Appendix 2. Trial Flow Chart of Dose Escalation Phase - Q2W Administration

| Visit Time Points | Screening | Cycle 1-2 | | Extended Trial | Conclusion/Withdrawal |
| --- | --- | --- | --- | --- | --- |
|  | -28 | D 1 (±1) of Cycle 1 | D 1 (±1) of Cycle 2 | D 1 (±2) of Cycle 3-n |  |
| Informed Consent | √ |  |  |  |  |
| **General Medical History Collection** |  |  |  |  |  |
| Inclusion/Exclusion Review | √ |  |  |  |  |
| Filling of General Information | √ |  |  |  |  |
| Past medical history | √ |  |  |  |  |
| Combined diseases and treatment history | √ |  |  |  |  |
| **ECOG** Scale | √ | √ |  |  |  |
| Virology test | √ |  |  |  |  |
| **Safety Examination** |  |  |  |  |  |
| Vital signs | √ | √ | √ | √ | √ |
| Routine blood test | √ | **√△** | **√** | √ | √ |
| Routine Urinalysis | √ | **√△** | **√** | √ | √ |
| Blood chemistry | √ | **√△** | **√** | √ | √ |
| Blood coagulation test | √ | **√△** | **√** |  |  |
| Pharmacokinetics and Immunogenicity |  | See Item 6 in the Clinical Trial Protocol | |  |  |
| Electrocardiography | √ | √※ | √※ | √☆ | √ |
| Urine pregnancy test | √ |  |  |  | √ |
| Echocardiography | √ | √★ | | | √ |
| HER2 Detection | √ |  | | |  |
| Tumor Marker ▲ | √ | **√△** |  | √ |  |
| Imaging Examination# | √ | Once every 2 cycles | | |  |
| Efficacy Evaluation |  | Once every 2 cycles | | |  |
| Record the adverse events |  | √ | √ | √ | √ |
| Record concomitant medication | √ | √ | √ | √ | √ |

△ If the interval between the 1st dose and the screening period should be no more than 7 days, no reexamination is required.

※ Perform ECG examination 3 times at intervals of 5 minutes before each dose. Monitor ECG during medication. Perform ECG examination 3 times at intervals of 5 minutes within 1-2 hours after administration.

★ If the subject experiences precordial chest pain, palpitations or other symptoms, or has an abnormal ECG, CK-MB test and echocardiography may be added.

▲ Specific test items will be determined by the investigators depending on the tumor type.

# At baseline, all patients with solid tumors must undergo CT/MRI scans of the chest, abdomen and pelvis according to the RECIST v1.1. For osteopathy patients, confirmatory bone scan shall be carried out at the time of baseline. Tumor assessment will be performed by using CT/MRI/radiography. For subjects rated as CR or PR at the initial evaluation, their efficacy should be verified 4 weeks later. 1 evaluation should be performed every 2 cycles.

☆ ECG is required before each dose.

Note: Each treatment cycle includes 2 doses, and the efficacy should be evaluated every 2 treatment cycles.

# Appendix 3. Trial Flow Chart of Dose Escalation Phase - Q3W Administration

| Visit Time Points | Screening | Trial stage | | | Extended Trial | Conclusion/Withdrawal |
| --- | --- | --- | --- | --- | --- | --- |
|  | -28 | D 1 (±1) of Cycle 1 | D 1 (±1) of Cycle 2 | D 1 (±1) of Cycle 3 | D 1 (±2) of Cycle 3-n |  |
| Informed Consent | √ |  |  |  |  |  |
| **General Medical History Collection** |  |  |  |  |  |  |
| Inclusion/Exclusion Review | √ |  |  |  |  |  |
| Filling of General Information | √ |  |  |  |  |  |
| Past medical history | √ |  |  |  |  |  |
| Combined diseases and treatment history | √ |  |  |  |  |  |
| **ECOG** Scale | √ | √ |  |  |  |  |
| Virology test | √ |  |  |  |  |  |
| **Safety Examination** |  |  |  |  |  |  |
| Vital signs | √ | √ | √ | √ | √ | √ |
| Routine blood test | √ | **√△** | **√** | **√** | √ | √ |
| Routine Urinalysis | √ | **√△** | **√** | **√** | √ | √ |
| Blood chemistry | √ | **√△** | **√** | **√** | √ | √ |
| Blood coagulation test | √ | **√△** | **√** | **√** |  |  |
| Pharmacokinetics and Immunogenicity |  | See Item 6 in the Clinical Trial Protocol | | |  |  |
| Electrocardiography | √ | √※ | √※ | √※ | √☆ | √ |
| Urine pregnancy test | √ |  |  |  |  | √ |
| Echocardiography | √ | √★ |  |  |  | √ |
| HER2 Detection | √ |  |  |  |  |  |
| Tumor Marker ▲ | √ | **√△** |  |  | √ |  |
| Imaging Examination# | √ | Once every 3 cycles | | |  |  |
| Efficacy Evaluation |  | Once every 3 cycles | | |  |  |
| Record the adverse events |  | √ | √ | √ | √ | √ |
| Record concomitant medication | √ | √ | √ | √ | √ | √ |

△ If the interval between the 1st dose and the screening period should be no more than 7 days, no reexamination is required.

※ Perform ECG examination 3 times at intervals of 5 minutes before each dose. Monitor ECG during medication. Perform ECG examination 3 times at intervals of 5 minutes within 1-2 hours after administration.

★ If the subject experiences precordial chest pain, palpitations or other symptoms, or has an abnormal ECG, CK-MB test and echocardiography may be added.

▲ Specific test items will be determined by the investigators depending on the tumor type.

# At baseline, all patients with solid tumors must undergo CT/MRI scans of the chest, abdomen and pelvis according to the RECIST v1.1. For osteopathy patients, confirmatory bone scan shall be carried out at the time of baseline. Tumor assessment will be performed by using CT/MRI/radiography. For subjects rated as CR or PR at the initial evaluation, their efficacy should be verified 4 weeks later. 1 evaluation should be performed every 2 cycles.

☆ ECG is required before each dose.

Note: Each treatment cycle includes 1 dose, and the efficacy should be evaluated every 2 treatment cycles.

# Appendix 4. Flow Chart of Extended Trial - Q2W Administration

| Visit Time Points | -28 | Cycle 1  1d (±1) | Cycle 2  1d (±1) | Cycle 3-n  1d (±2) | Conclusion/Withdrawal |
| --- | --- | --- | --- | --- | --- |
| Informed Consent | √ |  |  |  |  |
| **General Medical History Collection** |  |  |  |  |  |
| Inclusion/Exclusion Review | √ |  |  |  |  |
| Filling of General Information | √ |  |  |  |  |
| Past medical history | √ |  |  |  |  |
| Combined diseases and treatment history | √ |  |  |  |  |
| **ECOG** Scale | √ | √ |  |  |  |
| Virology test | √ |  |  |  |  |
| **Safety Examination** |  |  |  |  |  |
| Vital signs | √ | √ | √ | √ | √ |
| Routine blood test | √ | **√△** | **√** | √ | √ |
| Routine Urinalysis | √ | **√△** | **√** | √ | √ |
| Blood chemistry | √ | **√△** | **√** | √ | √ |
| Blood coagulation test | √ | **√△** | **√** |  |  |
| Pharmacokinetics and Immunogenicity |  | See Item 6 in the Clinical Trial Protocol | | |  |
| Electrocardiography | √ | √※ | √※ | √☆ | √ |
| Urine pregnancy test | √ |  |  |  | √ |
| Echocardiography | √ | √★ | | | √ |
| HER2 Detection | √ |  | | |  |
| Tumor Marker ▲ | √ | **√△** |  | √ |  |
| Imaging Examination# | √ | Once every 2 cycles | | |  |
| Efficacy Evaluation |  | Once every 2 cycles | | |  |
| Record the adverse events |  | √ | √ | √ | √ |
| Record concomitant medication | **√** | **√** | **√** | **√** | **√** |

△ If the interval between the 1st dose and the screening period should be no more than 7 days, no reexamination is required.

※ Perform ECG examination 3 times at intervals of 5 minutes before each dose.

★ If the subject experiences precordial chest pain, palpitations or other symptoms, or has an abnormal ECG, CK-MB test and echocardiography may be added.

▲ Specific test items will be determined by the investigators depending on the tumor type.

# At baseline, all patients with solid tumors must undergo CT/MRI scans of the chest, abdomen and pelvis according to the RECIST v1.1. For osteopathy patients, confirmatory bone scan shall be carried out at the time of baseline. Tumor assessment will be performed by using CT/MRI/radiography. For subjects rated as CR or PR at the initial evaluation, their efficacy should be verified 4 weeks later. 1 evaluation should be performed every 2 cycles.

☆ ECG is required before each dose.

Note: Each treatment cycle includes 2 doses, and the efficacy should be evaluated every 2 treatment cycles.

# Appendix 5. Trial Flow Chart of Dose Escalation Phase - QW Administration

| Visit Time Points | Screening | Cycle 1-2 | | Extended Trial | Conclusion/Withdrawal |
| --- | --- | --- | --- | --- | --- |
|  | -28 | D 1 (±1) of Cycle 1 | D 1 (±1) of Cycle 2 | D 1 (±2) of Cycle 3-n |  |
| Informed Consent | √ |  |  |  |  |
| **General Medical History Collection** |  |  |  |  |  |
| Inclusion/Exclusion Review | √ |  |  |  |  |
| Filling of General Information | √ |  |  |  |  |
| Past medical history | √ |  |  |  |  |
| Combined diseases and treatment history | √ |  |  |  |  |
| **ECOG** Scale | √ | √ |  |  |  |
| Virology test | √ |  |  |  |  |
| **Safety Examination** |  |  |  |  |  |
| Vital signs | √ | √ | √ | √ | √ |
| Routine blood test | √ | **√△** | **√** | √ | √ |
| Routine Urinalysis | √ | **√△** | **√** | √ | √ |
| Blood chemistry | √ | **√△** | **√** | √ | √ |
| Blood coagulation test | √ | **√△** | **√** |  |  |
| Pharmacokinetics and Immunogenicity |  | See Item 6 in the Clinical Trial Protocol | |  |  |
| Electrocardiography | √ | √※ | √※ | √☆ | √ |
| Urine pregnancy test | √ |  |  |  | √ |
| Echocardiography | √ | √★ | | | √ |
| HER2 Detection | √ |  | | |  |
| Tumor Marker ▲ | √ | **√△** |  | √ |  |
| Imaging Examination# | √ | Once every 2 cycles | | |  |
| Efficacy Evaluation |  | Once every 2 cycles | | |  |
| Record the adverse events |  | √ | √ | √ | √ |
| Record concomitant medication | √ | √ | √ | √ | √ |

△ If the interval between the 1st dose and the screening period should be no more than 7 days, no reexamination is required.

※ Perform ECG examination 3 times at intervals of 5 minutes before each dose. Monitor ECG during medication. Perform ECG examination 3 times at intervals of 5 minutes within 1-2 hours after administration.

★ If the subject experiences precordial chest pain, palpitations or other symptoms, or has an abnormal ECG, CK-MB test and echocardiography may be added.

▲ Specific test items will be determined by the investigators depending on the tumor type.

# At baseline, all patients with solid tumors must undergo CT/MRI scans of the chest, abdomen and pelvis according to the RECIST v1.1. For osteopathy patients, confirmatory bone scan shall be carried out at the time of baseline. Tumor assessment will be performed by using CT/MRI/radiography. For subjects rated as CR or PR at the initial evaluation, their efficacy should be verified 4 weeks later. 1 evaluation should be performed every 2 cycles.

☆ ECG is required before each dose.

Note: Each treatment cycle includes 2 doses, and the efficacy should be evaluated every 2 treatment cycles.
